# Defining the Chronic Complexities of hEDS and HSD: A Global Survey of Diagnostic Challenges, Life-Long Comorbidities, and Unmet Needs

**DOI:** 10.3390/jcm14165636

**Published:** 2025-08-09

**Authors:** Victoria Daylor, Molly Griggs, Amy Weintraub, Rebecca Byrd, Taylor Petrucci, Matthew Huff, Kathryn Byerly, Roman Fenner, Sydney Severance, Charlotte Griggs, Amol Sharma, Paldeep Atwal, Steven A. Kautz, Steven Shapiro, Kimberly Youkhana, Mark Lavallee, Allison Wilkerson, Michelle Nichols, Alan Snyder, Josef K. Eichinger, Sunil Patel, Anne Maitland, Cortney Gensemer, Russell A. Norris

**Affiliations:** 1 Department of Regenerative Medicine and Cell Biology, Medical University of South Carolina, Charleston, SC 29407, USA; daylor@musc.edu (V.D.); griggsma@musc.edu (M.G.); weintrau@musc.edu (A.W.); byrdre@musc.edu (R.B.); petrucct@musc.edu (T.P.); huffmat@musc.edu (M.H.); byerlyk@musc.edu (K.B.); fennerro@musc.edu (R.F.); severans@musc.edu (S.S.); griggsch@musc.edu (C.G.); gensemer@musc.edu (C.G.); 2 Department of Neurosurgery, Medical University of South Carolina, Charleston, SC 29407, USA; patels@musc.edu; 3 Division of Gastroenterology, Medical University of South Carolina, Charleston, SC 29407, USA; sharmaam@musc.edu; 4 Atwal Clinic, Palm Beach, FL 33401, USA; dra@atwalclinic.com; 5 Department of Health Science and Research, Medical University of South Carolina, Charlston, SC 29407, USA; kautz@musc.edu (S.A.K.); nicholmg@musc.edu (M.N.); 6 Division of Pediatric Dentistry, Medical University of South Carolina College of Dental Medicine, Charlston, SC 29407, USA; stevenshapiro@comcast.net; 7 Division of Hematology and Oncology, Medical University of South Carolina, Charlston, SC 29407, USA; youkhana@musc.edu; 8 Department of Orthopedics and Sports Medicine, UPMC-Central PA, Harrisburg, PA 17109, USA; marklavallee@icloud.com; 9 Department of Psychiatry and Behavioral Sciences, Medical University of South Carolina, Charlston, SC 29407, USA; wilkersa@musc.edu; 10 Department of Dermatology, Medical University of South Carolina, Charlston, SC 29407, USA; snydeala@musc.edu; 11 Department of Orthopedic Surgery and Physical Medicine, Medical University of South Carolina, Charlston, SC 29407, USA; eichinge@musc.edu; 12 Division of Rheumatology and Immunology, Medical University of South Carolina, Charlston, SC 29407, USA; maitlana@musc.edu

**Keywords:** chronic conditions, Ehlers–Danlos syndrome, hypermobility

## Abstract

**Background:** Hypermobile Ehlers–Danlos syndrome (hEDS) and hypermobility spectrum disorders (HSDs) are prevalent, complex conditions marked by chronic pain, joint instability, and multisystem involvement. Despite affecting an estimated 1 in 500 individuals, these conditions remain poorly understood and inconsistently diagnosed. This study aimed to define their clinical burden through a large-scale, international survey. **Methods:** A cross-sectional, anonymous survey was distributed globally from September 2023 to March 2024. Of 9258 responses, 3906 met inclusion criteria (hEDS: n = 3360; HSD: n = 546). The 418-item questionnaire assessed symptoms, comorbidities, healthcare utilization, and quality of life. The 95% confidence intervals (CIs) were calculated for comparison. **Results:** Participants with hEDS reported a mean of 24 comorbid conditions and an average diagnostic delay of 22.1 years. Common diagnoses included gastrointestinal disorders (84.3% [95% CI: 98.3–99.2%]), dysautonomia (71.4% [95% CI: 69.9–72.9%]), and chronic pain (98.9% [95% CI: 98.3–99.2%]). In contrast, HSD respondents reported a mean of 17 comorbidities, a 17.5-year time to diagnosis, and lower rates of key complications. Triggering events, such as puberty and infections were commonly reported preceding hEDS or HSD symptom onset. Comparison to the All of Us dataset revealed significantly elevated prevalence ratios of neurological, immune, and autonomic diagnoses. **Conclusions:** This global survey highlights the extensive multisystemic burden and diagnostic delays faced by individuals with hEDS and HSDs. The high prevalence of immune, neurological, gastrointestinal, and autonomic dysfunctions challenges the notion of these conditions as isolated connective tissue disorders. These findings highlight the need for updated diagnostic frameworks and mechanistic studies that explore multifactorial etiologies beyond the connective tissue paradigm.

## 1. Introduction

The Ehlers–Danlos syndromes (EDS) are a group of 14 heritable connective tissue disorders (CTDs) characterized by varying phenotypic presentation, often sharing commonalities of joint hypermobility, skin hyperextensibility, and ligament and tissue fragility [1,2]. This group of disorders is heterogeneous with most subtypes arising from mutations affecting collagen or other components of the extracellular matrix (ECM) [2,3]. While the majority of subtypes have clearly defined genetic markers, the most common subtype, hypermobile Ehlers–Danlos syndrome (hEDS), lacks a clear genetic or diagnostic test [2]. As such, hEDS is currently diagnosed clinically based on the 2017 criteria established by the International Consortium on the Ehlers–Danlos Syndromes and Related Disorders [2]. However, these criteria were developed through expert consensus rather than data-driven statistical validation and do not adequately capture the prevalence or clinical spectrum of comorbidities associated with hEDS and HSD. This has led to confusion among practitioners and inconsistency in diagnosis. Patients who exhibit symptomatic joint hypermobility but do not meet the full diagnostic criteria for hEDS are often labeled with hypermobility spectrum disorders (HSD), a classification that remains poorly defined and inconsistently applied [4]. Due to these diagnostic ambiguities, accurate country- or region-specific prevalence estimates are not possible to currently calculate. This underscores the need for more robust, data-driven definitions of these conditions. Although hEDS and HSD have historically been considered rare, recent regional studies challenge that assumption. For example, a national cohort study in Wales (UK) using linked primary care and hospital data reported a diagnosed prevalence of approximately 1 in 500 individuals [5], suggesting these conditions are more common than previously recognized and likely underdiagnosed in many populations.

Clinically, hEDS and HSD often present with overlapping features, including musculoskeletal complaints, extensive systemic involvement, and a range of comorbid conditions [5]. This may include neurological, immunological, cardiovascular, endocrine, genitourinary, dental, and gastrointestinal (GI) components [6,7,8,9]. Symptom onset typically occurs in early adolescence, with patients commonly reporting an increase in injuries, GI disturbances, and joint pain which are often mistaken for growing pains [10,11]. A triggering event, such as environmental, pubertal, or viral factors, is often reported near the onset of symptoms [12]. Patients may also experience overlapping comorbid conditions, including autonomic dysfunction, mast cell activation disease (MCAD) and cardiovascular manifestations such as mitral valve prolapse or aortic root dilatation [13]. Neurological complications like cerebrospinal fluid (CSF) leaks, cranio-cervical instability (CCI), tethered cord syndrome (TCS), and small-fiber neuropathy (SFN) are also reported [14]. Other structural manifestations include pelvic and rectal prolapse, hiatal hernias, Chiari malformation and spinal instabilities [7,8,15,16,17].

While these conditions are increasingly recognized in patients with hEDS and HSD, the full spectrum of associated manifestations remains poorly characterized. Many patients experience a lengthy journey to diagnosis due to the lack of a diagnostic test, barriers within the healthcare system, physician knowledge and attitudes, and the complexity of the clinical presentations [18]. This “diagnostic odyssey” can result in delays in care, reduced quality of life and worsening of symptoms over time without access to proper management [12,19].

Early perspectives helped shape the diagnostic criteria for hEDS and HSD, but these were developed in the absence of large-scale, population-based data and lacked statistical validation. Current clinical insights are primarily derived from small, single center cohorts that often exclude control groups and are limited in geographic representation, sample size, and phenotypic depth [2,20,21,22]. No prior studies have leveraged a large, multinational dataset to assess the multisystemic burden and diagnostic experience of individuals with hEDS and HSD across diverse healthcare systems and cultural contexts. To address this gap, we conducted a global, patient-centered survey to generate a comprehensive, statistically grounded view of these conditions. Our findings provide novel insights into symptom prevalence and comorbidity profiles with the goal of informing diagnostic criteria, advancing mechanistic understanding, and improving care strategies for this complex and underrecognized population.

## 2. Materials and Methods

### 2.1. Ethics Approval

The method of consent was reviewed and approved by the Institutional Review Board (IRB) at the Medical University of South Carolina, which determined the study to be exempt under category 2 (Consent ID: Pro00129825, dated 13 September 2023, with exemption status valid through 13 September 2028). Inclusion criteria were that all participants must be at least 18 years), have a clinical diagnosis of Ehlers–Danlos Syndrome or a hypermobility spectrum disorder, and have given informed consent by completing the survey. No minors were included in the study. As is common in online survey research, where inclusion criteria are used to guide participation, there was no exclusion criteria for survey participation. Instead, inclusion criteria are used to guide participation, and responses are later screened for eligibility. The start of recruitment was 14 September 2023, and recruitment ended 14 March 2024. This study complies with the Declaration of Helsinki in that this observational, non-interventional study posed no physical or psychological risk, and consent was provided through an IRB-approved consent process, appropriate for anonymous, exempt survey research [23]. No identifying information was collected, and no questions solicited protected health information (PHI). All responses were anonymous and reported in aggregate.

### 2.2. Survey Design

This study employed a cross-sectional survey design to assess patients with hEDS or HSD using self-reported data collected on a secure, web-based online research survey platform, REDCap (Research Electronic Data Capture) [24,25]. The survey was developed through an iterative, multidisciplinary collaboration with researchers, medical specialists who manage high volumes of hEDS/HSD patients, and individuals diagnosed with these conditions. Diagnostic sections of the survey were designed by specialists in relevant fields to ensure clinical relevance. The instrument was pilot tested with a cohort of 10 patients with hEDS or HSD, whose feedback informed refinements in clarity, flow, and accessibility. The final survey aimed to capture a comprehensive profile of symptoms, comorbidities, and diagnostic experiences across organ systems.

The survey was conducted anonymously and consisted of 418 items, covering 192 conditions, along with associated symptoms (Supplementary Figures S1 and S2). To reduce misclassification, diagnostic items were repeated using parallel language to distinguish confirmed diagnoses from suspected conditions. To be eligible to participate, respondents had to be at least 18 years old and report a formal diagnosis of EDS or HSD. All responses were later reviewed for eligibility in the final analysis. All survey fields were required, and only entirely completed surveys were analyzed, minimizing missing data. Participants were recruited online through social media, community organizations, and clinicians.

### 2.3. Statistical Analyses

**Figure 1:**
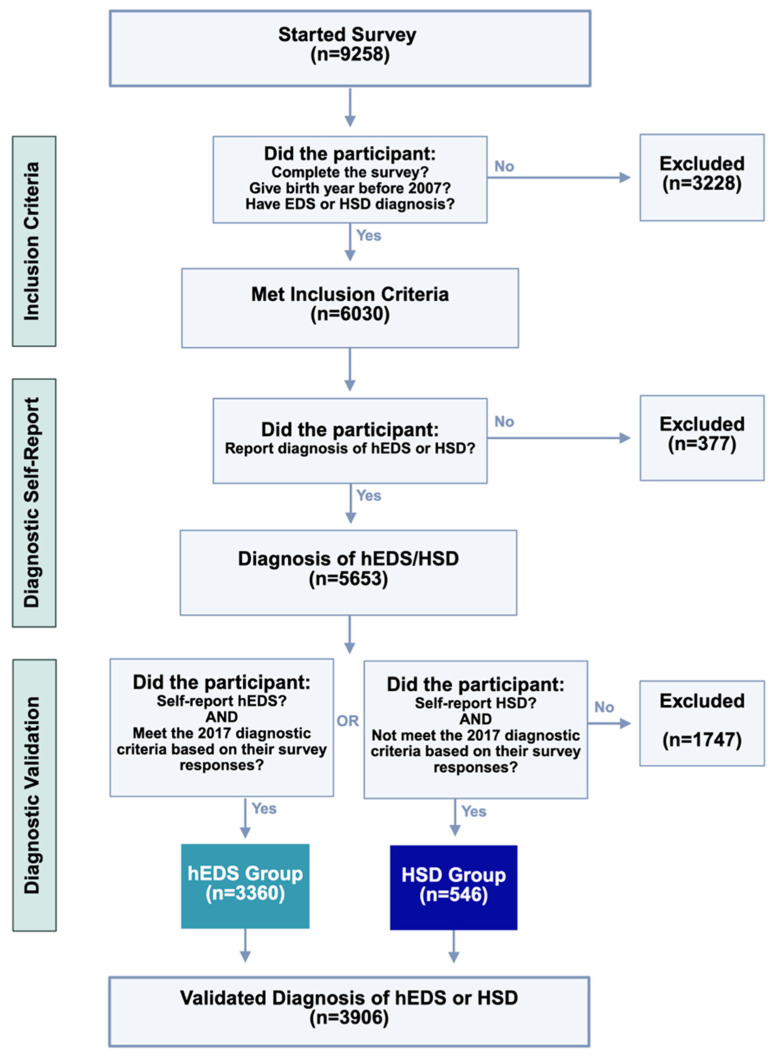
**Criteria and diagnostic validation steps for survey respondents.** Of 9258 individuals who began the survey, participants were progressively excluded for age, incomplete data, or inconsistent diagnoses. Final groups (hEDS: n = 3360; HSD: n = 546) reflect participants who both self-reported and met criteria consistent with diagnostic classification.

Survey results (n = 9258) were directly imported from RedCap using the RedCapTidieR package in R [26]. As shown in Figure 1, we filtered for individuals who reported a diagnosis of hEDS or HSD, were at least 18 years old, and completed the full survey (n = 5653). Their self-reported hEDS and HSD diagnoses were validated using the first two criteria of the 2017 hEDS Diagnostic Criteria [2] through an R script. To mitigate bias in self-reported responses, diagnostic criteria questions were deliberately dispersed throughout the questionnaire to reduce the likelihood that participants would recognize and tailor their answers to the diagnostic framework. For Criterion 1, participants aged 18–50 with a Beighton score of ≥5, as well as those over 50 with a Beighton score of ≥4, were considered to have met the criterion. For Criterion 2, Features A, B, and C were assessed, with participants meeting at least two of these categories considered as satisfying the criterion. Criterion 3 was not applied due to the inability to verify physical examination findings. This process yielded two analysis groups: hEDS patients meeting the 2017 hEDS diagnostic criteria (hEDS group), and HSD patients who did not meet the hEDS criteria (HSD group). Those who reported hEDS but did not meet the criteria (n = 1183) and those reporting HSD who did meet hEDS criteria (n = 564) were excluded from analyses. No duplicates were identified. Due to the prevalence of certain conditions in the dataset, similar answer choices (e.g., “irritable bowel syndrome—Constipation” and “irritable bowel syndrome—Mixed”) were grouped as a single disorder for analysis unless otherwise specified. A subset of survey questions was selected for statistical analysis, with additional exclusions applied to items that had fewer than five responses, following the “Rule of 5” for normal approximation [27]. To compare hEDS and HSD groups, categorical variables were analyzed using Chi-square tests. Contingency tables were constructed, and Bonferroni correction was applied to adjust for multiple comparisons (n = 365), using this conservative approach to minimize false positives given the large number of tests and self-reported nature of the data. Risk ratios and 95% confidence intervals were calculated from contingency tables comparing the proportion of individuals with each diagnosis in the hEDS group to the HSD group. A corrected *p*-value of ≤0.05 was considered statistically significant.

To evaluate the prevalence of comorbidities in the hEDS group relative to the general population, aggregate-level data from the All of Us data browser (n = 354,400) were used. This study used data from the All of Us Research Program’s Controlled Tier Dataset 7, available to authorized users on the Researcher Workbench. Survey items related to formal medical diagnoses were included in this comparison if the hEDS group had ≥5 responses and the All of Us dataset had ≥40 responses. Chi-square analyses were performed with Bonferroni correction applied to 138 comparisons, using a significance threshold of *p* ≤ 0.05. Risk ratios and 95% confidence intervals were calculated from contingency tables comparing the proportion of individuals with each diagnosis in the hEDS group to the All of Us population. For continuous variables, outliers were identified using the ROUT method (Q = 1%) in GraphPad prior to conducting unpaired Welch’s *t*-tests on the cleaned dataset. A *p*-value of ≤0.05 was used to define statistical significance.

To visualize patterns of co-occurring conditions in hEDS patients, a co-morbidity matrix was constructed for conditions that were reported as formal diagnoses. Responses marked “None of the Above” were excluded. The matrix was normalized using the Jaccard Index, which quantified the degree of overlap between each pair of co-morbidities by dividing the number of patients with both conditions by the total number of patients with either condition. For clarity, only the most frequently overlapping conditions were included in the final heatmap by selecting conditions based on the sum of their Jaccard indices, ensuring legibility. An overlap threshold of 13.5 was selected to correspond with the 85th percentile of overlap scores. Self-overlapping conditions and redundant pairings were removed, resulting in a triangular matrix format. The heatmap was generated using the ComplexHeatmaps package [28,29].

## 3. Results

### 3.1. Study Cohort

#### 3.1.1. Eligibility Criteria

A total of 9258 participants initially started the survey. Among these, 5653 participants reported a diagnosis of hEDS or HSD. These participant responses underwent a diagnostic validation process using the 2017 criteria. Ultimately, the final validated sample consisted of 3906 participants who had either a validated diagnosis of hEDS (hEDS group; n = 3360) or HSD (HSD group; n = 546). This process ensured that only individuals meeting the established diagnostic criteria were included in the final dataset.

#### 3.1.2. Demographics

**Figure 2:**
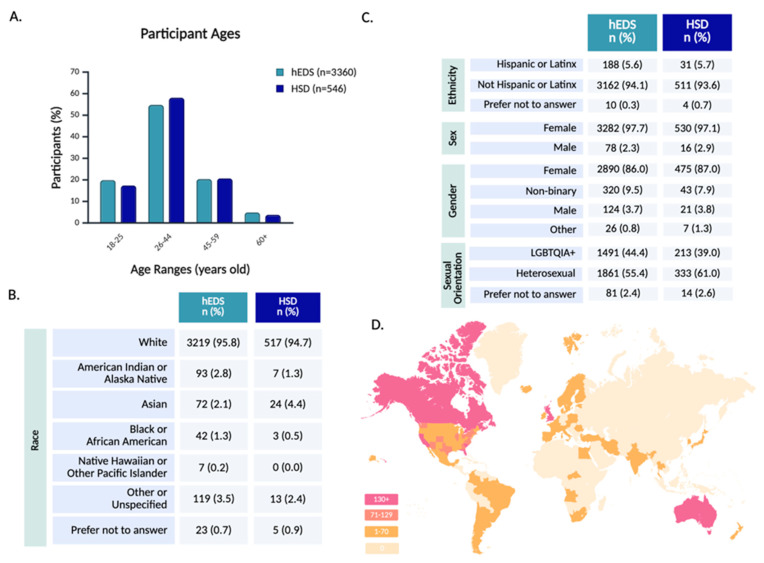
**Participant demographics and global representation.** Participant demographics are shown across age (**A**), race (**B**), and identity-related variables including ethnicity, sex, gender, and sexual orientation (**C**). Panel (**D**) depicts the geographic distribution of participants by country and U.S. state, with darker shading indicating higher regional response counts.

The survey sample population was primarily composed of younger and middle-aged adults, with the largest proportion falling between the ages of 26 and 44 (Figure 2A). Older adults (60+) represented only a small subset of the sample, potentially reflecting barriers to diagnosis or participation in online research among older populations.

Racial demographics indicated a predominantly White sample (Figure 2B), and ethnicity data indicated that a minority of participants identified as Hispanic or Latinx (Figure 2C). This relative lack of racial diversity mirrors broader disparities in EDS and HSD diagnosis, as studies suggest these conditions are underrecognized in non-White populations [30].

The majority of participants were assigned female at birth, aligning with prior research that highlights a diagnostic bias toward females [15,31]. In terms of gender, 86.0% of hEDS and 87.0% of HSD participants identified as female, with a higher rate of non-binary participants than male participants. While the majority of participants were cisgender females, gender diversity within the cohort was notable, with 12.0% of all respondents identifying with a gender different from their sex assigned at birth (Supplementary Table S1). Sexual orientation among respondents was highly diverse, with a large proportion identifying as part of the LGBTQIA+ spectrum (Figure 2C and Supplementary Table S2).

Geographically, 67.5% of all respondents resided in the United States, with representation from every state, including notable participation from California, South Carolina, Florida, and Texas (Figure 2D and Supplementary Table S3). Globally, the survey reached participants on every continent, besides Antarctica, with strong response rates from the United Kingdom, Australia, Canada, New Zealand, and the Netherlands (Supplementary Table S4). This worldwide engagement highlights the survey’s reach across diverse geographic and healthcare landscapes.

### 3.2. Diagnostic Journey

**Figure 3:**
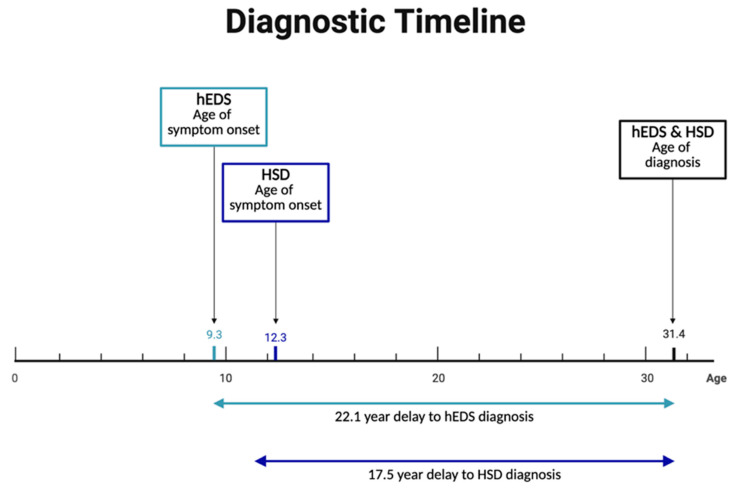
**Prolonged diagnostic delays in hEDS and HSD.** Despite early symptom onset—9.3 years for hEDS and 12.3 years for HSD—diagnoses were typically not made until age 31.4, resulting in average diagnostic delays of 22.1 and 17.5 years, respectively.

On average, participants with hEDS reported their symptom onset at the age of 9.3 but were diagnosed at the age of 31.4, with a delay in diagnosis from time of symptom onset of 22.1 years (Figure 3). Participants with HSD reported significantly later symptom onset but were diagnosed at the same age as hEDS participants, leading to a shorter, though still lengthy, delay from symptom onset to diagnosis. Both hEDS and HSD groups indicated their initial symptoms were musculoskeletal, gastrointestinal, or autonomic (Supplementary Table S5). The first suspicion of hEDS/HSD came most often from medical doctors, followed by physical therapists, and themselves or a friend/family member. Participants with hEDS were significantly more likely to be diagnosed by geneticists than patients with HSD. Rheumatologists were the most common diagnosticians for HSD, and the second most common for hEDS. Prior to diagnosis, more than half of participants report being misdiagnosed with conditions including fibromyalgia, anxiety, and growing pains.

Triggering events were common in this population of patients. 70.1% of individuals with hEDS and 65.0% of those with HSD identified a specific event preceding or heightening their symptoms (Table 1). Puberty and pathogens (viral or bacterial) were the most commonly reported triggers.

**Figure 4:**
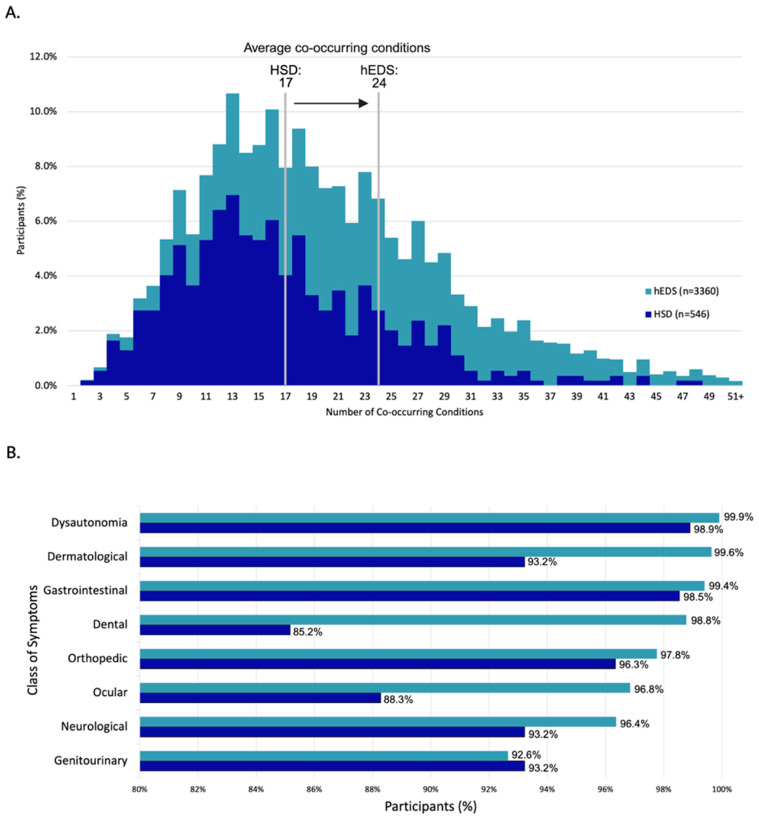
**Burden and distribution of co-occurring conditions in survey participants.** (**A**) Distribution of co-occurring conditions per participant, with group averages of 24 (hEDS) and 17 (HSD). (**B**) Percentage of participants reporting symptoms across eight major organ system categories.

Participants reported a high burden of co-occurring conditions, with hEDS participants averaging 24 and HSD participants 17, based on 192 conditions assessed in the survey (Figure 4A and Supplementary Figure S2). Participants reported experiencing a range of symptoms related to dysautonomia, dermatological issues, and gastrointestinal concerns (Figure 4B). When asked to rank the three most severe symptom categories from a provided list, responses were weighted by rank to reflect overall severity. Chronic pain was most frequently ranked as the most severe symptom by both groups (Table 2, Supplementary Table S6). However, notable differences emerged between groups: hEDS participants more often prioritized gastrointestinal symptoms, while HSD participants more frequently ranked joint-related symptoms as most severe.

To evaluate the impact of hEDS and HSD on daily functioning and productivity, we assessed the time patients spent managing their healthcare. A substantial proportion of individuals reported spending five or more hours per week, on average, coordinating and receiving medical care (Table 3). Participants indicated that they see multiple medical specialists annually, with hEDS participants averaging a higher number than HSD participants in the past year. The most frequently visited specialties included family medicine, cardiology, gastroenterology, neurology/neurosurgery, and emergency medicine.

Inpatient hospitalizations were more common in hEDS participants compared to HSD participants. More hEDS participants reported being admitted to the hospital at least once in the last year compared to HSD participants. When asked the furthest distance participants had traveled to receive medical care pertaining to hEDS/HSD, 31.3% of hEDS and 9.5% of HSD participants report having traveled 300 or more miles, with a subset of participants that have traveled more than 2000 miles. Regarding the financial burden of these diseases, many participants report paying out-of-pocket for medical care from a specialist not covered by insurance.

### 3.3. Multisystemic Clinical Presentation

#### 3.3.1. Pain

Chronic pain is a key feature of symptomatic joint hypermobility, reported in almost all hEDS and HSD participants (Table 4). The most frequently reported pain sites were the neck, lower back, and shoulder(s) in both groups.

**Figure 5:**
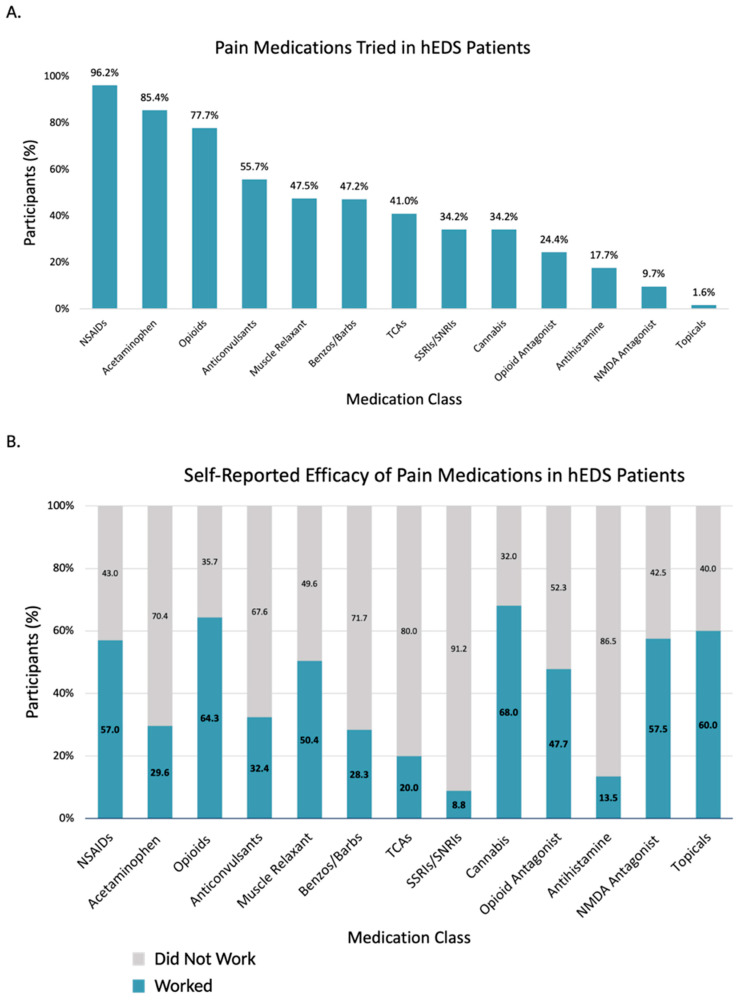
Pain medication use and perceived efficacy in hEDS participants (n = 3360). (**A**) Percentage of hEDS participants reporting use of each medication class. (**B**) Self-reported effectiveness of each medication class, shown as proportion who reported it worked.

Given the pain burden in these populations, pain management is a common concern for patients. Responses of participants with hEDS were analyzed regarding which medications they have tried for their pain, and of those, which medications participants considered effective (Figure 5, Supplementary Table S7). hEDS participants report medical marijuana and ketamine as the most effective for pain. Recently, selective serotonin reuptake inhibitors (SSRIs) and serotonin-norepinephrine reuptake inhibitors (SNRIs) have been increasingly used for pain, but only small proportion of hEDS participants that have tried SS/SNRIs for pain considered them effective. With limited treatment options available, participants report turning to non-traditional practices for their treatment, including massage, meditation, and acupuncture (Table 5). A significant proportion of participants also experienced complications related to local and/or general anesthesia (Table 6). The hEDS group was over two times likelier to experience shortened effect, insufficient pain control, intubation complication, and anaphylaxis.

#### 3.3.2. Orthopedic

Musculoskeletal complications were highly prevalent in both hEDS and HSD. Slipping rib syndrome was more prevalent in hEDS participants compared to HSD participants and the All of Us dataset (Table 7). Additionally, participants with hEDS were over 40 times more likely to report tendon rupture than participants in the All of Us dataset. Of note, hEDS participants are at a greater risk of unexplained fractures than HSD participants (Table 8). Correspondingly, a much smaller proportion of hEDS participants reported having none of these joint issues. The shoulders, hips, and knees were among the most frequently affected joints. Additionally, several anatomical abnormalities were reported in the hEDS group, including missing or extra ribs, sacralization of the L5 vertebrae, and missing or extra vertebrae (Table 9).

#### 3.3.3. Neurological

Migraine was the most frequently reported neurological diagnosis among both groups, while affecting significantly more hEDS participants than HSD participants (Table 10). hEDS participants also reported significantly higher rates of scoliosis, Raynaud’s phenomenon, upper cervical instability, and occipital neuralgia, when compared to both HSD and All of Us datasets. Other neurological abnormalities were more common in hEDS than HSD, particularly tinnitus, herniated disk(s), spinal stenosis, small-fiber neuropathy, Chiari malformation, kyphoscoliosis, and tethered cord syndrome. There was no significant difference in the prevalence of voice disorders between the three groups, while the hEDS group was at slightly lower risk of hearing impairment than the general population. Neurological symptoms were highly prevalent among survey respondents, with differences observed between the hEDS and HSD groups. Weakness was the most reported symptom, followed closely by muscle spasms (Table 11).

#### 3.3.4. Autonomic

Autonomic dysfunction was commonly reported among participants, with 71.4% of hEDS and 40.3% of HSD participants reporting at least one diagnosed autonomic disorder, with a higher prevalence of each type in the hEDS group (Table 12). Postural orthostatic tachycardia syndrome (POTS) was the most frequently diagnosed autonomic disorder, affecting hEDS participants at a higher rate than those with HSD. This analysis revealed an extraordinarily elevated risk of POTS in the hEDS group compared to the general population. Symptoms of dysautonomia were highly prevalent, with fatigue, dizziness, and brain fog being the most highly reported (Table 13). Heart palpitations and thermoregulatory dysfunction were also frequent, suggesting significant autonomic involvement in both groups. Despite these high symptom burdens, many hEDS participants and the majority of HSD participants reported having no formal autonomic diagnosis.

#### 3.3.5. Gastrointestinal

Gastrointestinal (GI) symptoms were common in both groups, with a higher prevalence of at least one GI disorder in the hEDS group compared to HSD (Table 14). Irritable bowel syndrome (IBS) was the most reported GI diagnosis. Among IBS subtypes, the mixed variant was most prevalent. The hEDS group had approximately 1.5 times higher risk of gastroesophageal reflux disease (GERD) compared to both the HSD and All of Us groups. Dysmotility was also more frequently diagnosed in hEDS than HSD, with the gastric type (“gastroparesis”) reported the most frequently in both groups. hEDS participants reported a significantly higher prevalence of 13 of 19 GI disorders that were compared to the All of Us dataset, while obesity, abdominal hernia, cholelithiasis, and liver disease were all significantly higher in the general population than hEDS. Notably, colorectal polyps and ulcerative colitis were not significantly different between the hEDS and All of Us groups.

The most frequently reported symptoms were abdominal pain, nausea, bloating, and constipation (Table 15). Severe GI dysfunction requiring feeding devices was significantly more frequent in hEDS than in HSD, with nasogastric, nasojejunal, and total parenteral nutrition being the most frequently utilized options by hEDS participants (Table 16). Similarly, colostomies and ileostomies, while rare overall, were present in both groups.

#### 3.3.6. Cardiopulmonary

The majority of respondents in both groups reported no diagnosed cardiovascular conditions (Table 17). Of those diagnosed with a cardiac condition, mitral valve defect was the most frequently reported in hEDS, while the most frequently reported issue reported by HSD participants were arrhythmias (other than supraventricular tachycardia and atrial fibrillation). Other structural heart abnormalities, including tricuspid valve defects and aortic valve, were present, but less frequent in both groups. Compared to the All of Us dataset, hEDS had significantly higher prevalences of mitral valve defects, stroke, and tricuspid valve defects. Contrastingly, lung disease and atrial fibrillation had a significantly lower prevalence in hEDS when compared to All of Us data. Many of these cardiopulmonary diseases showed an age-effect of diagnoses, with peak diagnoses in the 26–44-year-old age range (Supplementary Table S8).

#### 3.3.7. Endocrine

Endocrine disorders were reported in a subset of participants, with 26.0% of hEDS and 20.3% of HSD participants reporting one or more endocrine disorders (Table 18). Non-autoimmune hyperthyroidism occurred at over 11 times the rate in hEDS participants compared to the general population. The risk of central adrenal insufficiency was nearly fivefold higher in hEDS. Pineal cysts were over 160 times more likely to be reported in hEDS than in the general population. hEDS participants reported lower rates than the All of Us group for osteoporosis, type 2 diabetes, and hyperparathyroidism.

#### 3.3.8. Hematologic

Most participants reported no formal hematologic diagnoses (Table 19). hEDS participants were at almost 14 times higher risk for pernicious anemia than the general population. The risk of deep vein thrombosis (DVT) was over threefold higher in the hEDS group compared to the HSD group. Easy or severe bruising was reported by the majority of hEDS and HSD participants.

#### 3.3.9. Reproductive

Reproductive health conditions were frequently reported by hEDS participants. The risk of pelvic organ prolapse was nearly four times higher in hEDS compared to HSD (Table 20). Endometriosis was also more common in hEDS than in HSD. Compared to the general population, hEDS participants were at markedly increased risk for multiple reproductive disorders, including polycystic ovary syndrome, vaginismus, and pelvic congestion syndrome. Erectile dysfunction was the most frequently reported reproductive condition in participants assigned male at birth. The most frequently reported reproductive symptoms included pelvic pain, irregular periods, pain during sex, and bleeding during sex (Table 21). Among those assigned female at birth, participants experienced their first menstrual period at ages 12.3 (hEDS) and 12.4 (HSD) on average. Of participants who have been pregnant, the majority from both groups reported pregnancy complications, although hEDS participants reported a significantly higher prevalence. Pregnancy complications included spontaneous abortion, preterm labor, failure to progress, premature rupture of membranes, and stillbirth.

#### 3.3.10. Urological

Urinary disorders were significantly more prevalent in the hEDS group compared to HSD (Table 22). Recurrent urinary tract infections (UTIs) were 1.63 times more common in hEDS participants than in HSD participants. Voiding dysfunction occurred at nearly twice the rate in hEDS compared to HSD, and overactive bladder syndrome showed a similar increase in risk. Relative to the general population, these conditions were markedly more frequent, with recurrent UTIs over 130 times more common and voiding dysfunction nearly 880 times more common in the hEDS group.

#### 3.3.11. Dermatological

The prevalence of having at least one or more dermatological condition was similar between hEDS and HSD (Table 23). Among them, atopic dermatitis was the most frequently reported, followed by hyperhidrosis. While there were no statistical differences between diagnoses in the hEDS and HSD groups, the hEDS group had significantly higher risk of atopic dermatitis when compared to the All of Us group. Skin-related symptoms were highly prevalent among respondents, especially the hEDS group, which may reflect the inclusion of these symptoms in the 2017 hEDS diagnostic criteria (Table 24). Soft, velvety skin, abnormally stretchy skin, and unexplained stretch marks were all more highly prevalent in hEDS compared to HSD.

#### 3.3.12. Allergic and Immunologic

Allergic and immunologic conditions were frequently reported in both groups, with 89.7% of hEDS and 78.0% of HSD participants reporting at least one allergy-related disorder (Table 25). Allergies were present at a higher rate in hEDS participants than HSD participants. Compared to HSD, hEDS participants had increased risk of mast cell activation syndrome, chronic urticaria, and allergies. Relative to the general population, these risks were markedly elevated, with chronic urticaria being over 230 times more common and MCAS over 1000 times more common in hEDS. Additionally, anaphylaxis episodes were reported significantly more frequently in hEDS compared to HSD. In total, 29.9% of hEDS participants and 22.0% of those with HSDs reported at least one of the autoimmune conditions assessed in this study. Reported autoimmune conditions with comparable prevalence between groups included psoriasis, rheumatoid arthritis, and systemic lupus erythematosus.

#### 3.3.13. Ocular

Ophthalmologic disorders were common among both hEDS and HSD groups, with 82.1% of hEDS and 72.7% of HSD participants reporting one or more ocular disorders (Table 26). hEDS had notably higher prevalences, compared to All of Us data, of astigmatism, myopia, hyperopia, macular degeneration, and keratoconus, but a similar prevalence of retinal detachment. Ophthalmologic symptoms were also common, including light sensitivity, visual disturbances, dry eyes, and double vision (Table 27).

#### 3.3.14. Dental

Dental issues were reported in a majority of hEDS participants and many HSD participants (Table 28). Dental disorders were reported at high rates, including temporomandibular joint (TMJ) disorder, enamel defects, and early onset periodontitis. All dental disorders included in this survey were significantly more frequent in the hEDS group than the All of Us group. Symptoms reported included jaw pain, as well as subluxation and/or dislocation of the temporomandibular joint (TMJ) (Table 29).

#### 3.3.15. Mental Health and Sleep

Within these populations, a high prevalence of anxiety, depression, and post-traumatic stress disorder (PTSD) were reported (Table 30). Sleep disturbances were also widely reported, including insomnia, restless legs syndrome, and obstructive sleep apnea. Each mental health and sleep-related disorder included in this survey was reported at a significantly higher rate in hEDS compared to All of Us, except obstructive sleep apnea and substance use disorder, which were significantly lower in hEDS, with bipolar disorder showing no significant difference.

#### 3.3.16. Neurodivergence

Neurodivergent diagnoses were common among respondents, with 49.1% of hEDS and 39.7% of HSD participants reporting at least one neurodiversity-related disorder (Table 31). The most frequently reported diagnoses included attention deficit hyperactivity disorder (ADHD/ADD), autism spectrum disorder (ASD), and obsessive–compulsive disorder (OCD). When hEDS and All of Us data was compared, ADHD, ASD, OCD, and learning disorder were present at higher rates in hEDS than in the general population.

#### 3.3.17. Multimorbidity in hEDS

**Figure 6:**
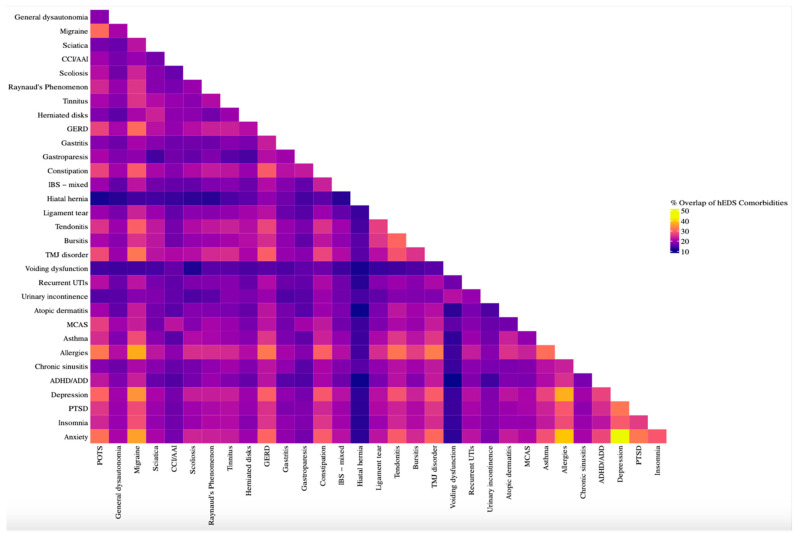
**Co-occurrence matrix of comorbidities in hEDS participants.** Heatmap showing pairwise overlap of formal diagnoses in hEDS participants (n = 3360). Percent overlap was calculated using the Jaccard Index, defined as the proportion of participants who reported both conditions among those who reported either. Conditions with higher overlap included anxiety, allergies, depression, insomnia, GERD, and POTS, highlighting multimorbidity clusters in autonomic, allergic, and psychiatric domains.

To examine the multimorbidity of co-occurring disorders, a matrix was created to illustrate the percentage of participants who reported both conditions among those who reported at least one (Figure 6). A clear pattern emerged among condition pairs with a Jaccard index of 28.0% or greater, revealing frequent overlap between allergies, migraine, anxiety, depression, POTS, GERD, constipation, tendonitis, TMJ disorder, insomnia, PTSD, bursitis, and asthma. Notably, the strongest co-occurrences were observed across psychiatric and immunologic domains, with anxiety–depression (43.3%), anxiety–allergies (39.3%), and depression–allergies (37.2%) ranking highest. Allergies and migraine also demonstrated a high rate of co-occurrence (37.0%).

## 4. Discussion

This study represents the largest global dataset to date characterizing the clinical and systemic burdens of hEDS and HSD. These findings reveal the critical need to appropriately address gaps in the continuum from diagnosis to treatment, as well as uncovering the underlying biological mechanisms of hEDS, HSD, and their associated comorbidities. Patient responses indicate challenges beginning with the diagnostic process, where significant delays, averaging over 20 years, and misdiagnoses are common due to limited awareness among healthcare providers and complex overlap between these multisystemic conditions. The overwhelming healthcare burden associated with hEDS and HSD has been previously reported [32] and is evident in the extensive time and financial resources patients devote to managing their conditions. Many participants in our survey reported spending five or more hours per week coordinating care, seeing an average of six specialists annually, and paying significant out-of-pocket costs for necessary treatments.

### 4.1. Multisystemic Manifestations

Our data support growing evidence of the substantial multisystemic involvement in hEDS and HSD, with early and severe manifestations including chronic pain, gastrointestinal symptoms, autonomic dysfunction, as well as joint instability. Autonomic dysfunction emerged as one of the most burdensome aspects of hEDS and, to a lesser extent HSD, with fatigue, dizziness, and orthostatic intolerance affecting the vast majority of participants. A significant proportion of participants also reported muscle weakness, numbness, tingling, and migraines, aligning with prior research linking hypermobility to small-fiber neuropathy (SFN) and dysautonomia [33,34,35]. Despite their inclusion in the 2017 hEDS diagnostic criteria, the prevalence of structural cardiac abnormalities, like mitral valve prolapse and aortic root dilatation, were relatively low in our dataset. This aligns with literature suggesting that autonomic and vascular dysfunction, rather than structural cardiac abnormalities, may be more clinically relevant for patients with hEDS and HSD [36].

Symptoms often attributed solely to hypermobility-related musculoskeletal issues appear to stem from coexisting conditions rather than hEDS itself. Autonomic dysfunction, MCAD, including MCAS, gastrointestinal dysmotility, and neurological complications can often drive symptom exacerbations, making it essential for healthcare providers to assess the broader clinical picture. GI symptoms were also reported at high rates in both hEDS and HSD groups, with gastroesophageal reflux disease, irritable bowel syndrome, and gastroparesis being particularly prevalent. These trends are consistent with prior research linking hypermobility to gut motility issues and visceral hypersensitivity, including studies demonstrating a higher prevalence of functional gastrointestinal disorders in individuals with hEDS and HSD [37,38].

Chronic pain was nearly universal among participants, with an average of six painful joints per participant. In addition to joint-related pain, participants frequently reported abdominal, neuropathic, headaches, and widespread pain. These findings align with prior literature on the biomechanical vulnerabilities in hypermobility-related disorders and emerging theories on pain sensitization in this population [39]. Despite this pain burden, conventional pharmacologic treatments remain largely ineffective, with patients frequently reporting poor responses to NSAIDs, antidepressants, and anticonvulsants. This may reflect clinical reports suggesting altered drug metabolism or receptor sensitivity in hypermobile individuals [40], underscoring the need for further investigation into pharmacogenetics, absorption differences, and alternative pain management strategies. The high prevalence of sleep disturbances further amplifies pain, fatigue, and cognitive function, highlighting the importance of targeted interventions to improve sleep.

A significant proportion of participants also reported immune-related conditions, including mast cell activation syndrome (MCAS), chronic urticaria, and heightened allergy-like responses. These findings align with prior research suggesting a potential immunological component in hypermobility disorders [40]. The frequent co-occurrence of immune dysregulation, neurological, gastrointestinal, and autonomic symptoms further underscore a complex and interconnected pathophysiology that remains poorly understood.

### 4.2. hEDS, HSD, and Rare Diseases

While some findings align with previous reports, the data also reveals a range of conditions beyond common comorbidities and symptoms, highlighting the overlap between hEDS, HSD, and some rare diseases. Participants in this study exhibited a disproportionately high prevalence of conditions such as Chiari malformation, cerebrospinal fluid leaks, tethered cord syndrome, adrenal insufficiency, gastroparesis, intracranial hypotension, and trigeminal neuralgia compared to the All of Us datasets. It is critical that healthcare providers consider the possibility of coexisting rare conditions in clinical settings. Relying solely on hEDS or HSD as the cause can lead to misdiagnosis and inadequate treatment, worsening patient distress and healthcare disparities.

### 4.3. Challenges of the Current Diagnostic Criteria

A central finding of this study is the significant overlap in symptoms and comorbidities between hEDS and HSD. The distinction between these conditions has been historically ambiguous, leading to diagnostic uncertainty. Strikingly, half of survey respondents with a reported HSD diagnosis met the diagnostic criteria for hEDS, while a quarter of those with an hEDS diagnosis did not meet the criteria. These findings highlight the urgent need for more precise and reliable diagnostic tools if these conditions are to be meaningfully differentiated in clinical practice. Follow-up analyses to further explore this diagnostic discordance are warranted to compare individuals who did or did not meet formal criteria across hEDS or HSD groups. One of the most apparent distinctions between hEDS and HSD is the degree of joint hypermobility measured by Beighton score. Participants with hEDS reported significantly higher hypermobility scores, along with high rates of joint dislocations, subluxations, and orthopedic. However, the Beighton score has recognized limitations, including its narrow focus on a limited number of joints, age and sex variability and inconsistent application by clinicians [41]. Notably, the three most frequently affected joints reported by hEDS participants—shoulders and hips—are not included in the Beighton score. These limitations may contribute to misclassification or underdiagnosis, particularly in individuals with systemic features but lower Beighton scores.

While these findings likely reflect the thresholds set by the 2017 diagnostic criteria for hEDS, they should not be interpreted to mean that HSD is less clinically significant. Symptom severity was not deeply assessed, and many respondents with HSD reported substantial symptom burden and multisystem involvement. Many comorbidities such as autonomic dysfunction, mast cell activation syndrome (MCAS), gastrointestinal dysmotility, and neurological complications were common in both groups, though more frequently reported in hEDS participants. The high symptom burden in both groups compared to the general population make differential diagnosis challenging. Future studies should focus on refining diagnostic criteria to ensure that individuals with disabling symptoms receive appropriate medical attention, regardless of whether they meet strict diagnostic thresholds. Expanding research into the underlying biological mechanisms of these conditions may also help clarify whether hEDS and HSD are truly separate conditions or variations within the same disorder spectrum.

### 4.4. Triggering Events

Over two-thirds of participants reported an event preceding symptom onset or worsening symptom severity, such as puberty, viral infections, physical trauma, or pregnancy. These findings reinforce the hypothesis that environmental factors, in combination with genetic predisposition, may play a role in the onset of hEDS and HSD and/or disease progression. The association between viral infections and worsening symptoms further supports a potential link between hEDS, immune dysregulation, and even post-viral syndromes such as long COVID and ME/CFS. The overlap of dysautonomia, MCAS, and chronic fatigue within these conditions suggest shared mechanisms, underscoring the need for further investigation to determine whether hypermobility-related disorders have a latent component that can be exacerbated by physiological stressors.

### 4.5. Limitations and Future Directions

While this study offers valuable insights, certain limitations are acknowledged. Despite extensive international outreach, the study population was predominantly composed of white, female, and digitally literate individuals. This may reflect a combination of systemic underdiagnosis in non-White populations, gender biases in diagnostic criteria and clinical recognition, and barriers to digital access or research participation in certain regions. As such, the current dataset may underrepresent individuals with limited access to specialized care, lower health literacy, or those from historically marginalized or underserved communities. These demographic imbalances introduce the possibility of selection bias and may limit the generalizability of findings across all racial, socioeconomic, and geographic populations. The reliance on self-reported data may introduce biases such as recall error and diagnostic uncertainty. In addition, self-selection bias may have influenced the study population, favoring participants with greater disease severity, health literacy, or digital access. Additionally, in some cases, missing or incomplete responses were received. Responses with incomplete data were excluded from relevant analyses, and no imputation methods were applied. The survey instrument, while developed iteratively and piloted in a patient cohort, has not undergone formal psychometric validation, which may limit the reliability of certain self-reported outcomes. This is consistent in conditions that are complex and under-characterized such as hEDS and HSD, where few validated instruments exist. In such contexts’, self-reported data are often the only practical means of capturing real-world, patient-centered experiences at scale. This approach is well-established in epidemiology, health services research, and rare disease registries, where it remains a critical tool for advancing understanding and improving care. Use of the 2017 criteria to filter participants may have excluded individuals with hEDS who do not meet current guidelines, and access to specialized care likely skewed responses to those with a formal diagnosis. The anonymous nature of the survey also prevented verification of unique responses. International differences in healthcare access and diagnostic practices may have also influenced how participants understood or reported their diagnoses. Furthermore, access to specialized testing (e.g., upright MRI for cranio-cervical instability) may vary significantly, potentially leading to underdiagnosis of certain conditions. Comparisons with the All of Us dataset should be interpreted with consideration, as demographic differences, particularly in age, sex, and racial composition, may influence observed patterns. While the findings provide important insights into symptom burden and comorbidity patterns, the cross-sectional design and lack of clinical verification preclude any causal inferences between reported symptoms and comorbidities. These limitations highlight the urgent need for large-scale, clinician-verified studies to corroborate these findings and refine diagnostic criteria.

Despite these limitations, the findings of this study highlight the profound, multisystemic, and lifelong impact of hEDS and HSD, encompassing chronic pain, autonomic dysfunction, immune and gastrointestinal involvement, and a wide spectrum of additional comorbidities. Yet, effective treatments remain elusive, leaving many patients to seek non-traditional and unproven therapies.

A major challenge in developing standardized care protocols is the lack of research that clearly defines and distinguishes hEDS from HSD, particularly regarding comorbidities not included in the 2017 diagnostic criteria. While existing studies have reported comorbid conditions in hEDS/HSD, most have been limited in scope, lump the two conditions together, or lacked control populations. The findings of this work define overlapping features and distinctions between hEDS and HSD, and their prevalence compared to the general population, providing a more complete understanding of these conditions. Distinguishing between them remains a challenge, as does understanding whether they exist on a continuous spectrum or if HSD can progress to hEDS over time or with exposure to additional triggers.

### 4.6. Reconsidering Etiology

While hEDS is currently classified as a heritable connective tissue disorder, findings from this study raise the hypothesis that connective tissue symptoms may develop in parallel with, or be influenced by, other systemic physiological processes. The observed high prevalence of immune dysregulation, autonomic dysfunction, and gastrointestinal dysmotility supports the possibility that neuroimmune or inflammatory pathways could contribute to the broader clinical phenotype. Although speculative and beyond the causal scope of this cross-sectional survey, these findings suggest that hEDS may, in some cases, reflect overlapping features with autoimmune or autoinflammatory conditions rather than representing a purely connective tissue–based pathophysiology. This hypothesis warrants further investigation through mechanistic and longitudinal studies. As researchers, clinicians and patient communities advocate for updated diagnostic frameworks, expanded research efforts, and improved treatment pathways, it is imperative that patient experiences remain at the forefront of these advancements. Given the significant multimorbidity associated with hEDS and HSD, future research, particularly incorporating biomarker studies and genetic approaches, may help refine classifications and improve our ability to diagnose and treat these conditions.

## 5. Conclusions

This global survey provides the most comprehensive epidemiological profile to date of individuals with hypermobile Ehlers–Danlos syndrome (hEDS) and hypermobility spectrum disorders (HSDs), revealing a strikingly high burden of multisystemic comorbidities, profound diagnostic delays, and significant unmet clinical needs. Across more than 3900 participants, the study identifies disproportionately high rates of gastrointestinal, autonomic, neurological, immune-mediated, and musculoskeletal complications, often with symptom onset in childhood, disease exacerbation following triggers, and formal diagnosis delayed by over two decades. Compared to both HSD and general population data, individuals with hEDS exhibited elevated risk for manifestations such as postural orthostatic tachycardia syndrome, small-fiber neuropathy, gastrointestinal dysmotility, immune-/mast cell-related conditions, and structural joint and spine abnormalities.

While these findings challenge the traditional view of hEDS and HSDs as isolated connective tissue disorders, we recognize that the study’s cross-sectional and self-reported design precludes causal inference. This study was not intended to establish new diagnostic criteria, but rather to generate a large, global dataset that can serve as a foundational resource for hypothesis generation and future studies. These results are meant to inform and complement emerging research in relation to genetic, proteomic, and biomarker signatures, which will be essential for developing validated, data-driven diagnostic frameworks. By illuminating the scope and heterogeneity of hEDS and HSD, this study highlights critical areas for future mechanistic investigation and reinforces the need for multidisciplinary, personalized approaches to care.

This study serves as a critical call-to-action for the medical community. The staggering delays in diagnosis, the overwhelming reliance on self-advocacy, and the systemic lack of treatment options illustrate a glaring gap in healthcare. The lack of standardized care protocols often leads to misdiagnosis, symptom neglect, and, at times, outright denial of these conditions by healthcare providers. Addressing these issues will require collaboration across specialties, heightened awareness among healthcare providers, and a sustained commitment to addressing the full spectrum of challenges faced by this patient population. Recognizing the full impact of hEDS and HSDs is key to driving meaningful change, breaking down barriers to care, advancing research and ensuring that individuals and families impacted by these conditions receive the medical care and support they deserve.

## Figures and Tables

**Table 1:** Symptom-onset triggers and COVID-related responses in hEDS and HSD.

	hEDS n (%)	HSD n (%)	hEDS vs. HSD RR (95% CI), *p*-Value †
Yes, event preceded symptoms of hEDS/HSD	2355 (70.1)	355 (65)	1.08 (1.01–1.15), ns
No	1005 (29.9)	191 (35)	0.86 (0.75–0.97), ns
Puberty	1009 (30)	118 (21.6)	1.39 (1.17–1.64), 0.0293
Viral or bacterial infection	860 (25.6)	126 (23.1)	1.11 (0.94–1.31), ns
Physical accident	551 (16.4)	75 (13.7)	1.19 (0.95–1.49), ns
Psychological or emotional event	481 (14.3)	86 (15.8)	0.91 (0.74–1.12), ns
Pregnancy	477 (14.2)	50 (9.2)	1.55 (1.18–2.04), ns
Other	470 (14)	68 (12.5)	1.12 (0.89–1.42), ns
Diagnosed with Long COVID	221 (6.6)	34 (6.2)	1.06 (0.74–1.50), ns
Diagnosed with Long COVID after hEDS/HSD	142 (4.2)	17 (3.1)	1.36 (0.83–2.23), ns
Diagnosed with Long COVID prior to hEDS/HSD	79 (2.4)	17 (3.1)	0.76 (0.45–1.27), ns

hEDS n = 3360; HSD n = 546; ns, not significant. † *p* values were calculated using Chi-square tests, with Bonferroni correction applied to adjust for multiple comparisons.

**Table 2:** Most severe symptoms reported by hEDS and HSD participants.

hEDS Most Severe Symptoms	hEDS	HSD Most Severe Symptoms	HSD
Chronic pain	32.6%	Chronic pain	34.2%
Gastrointestinal Symptoms	13.5%	Joint Manifestations	14.1%
Autonomic dysfunction	12.8%	Autonomic dysfunction	10.0%
Joint Manifestations	12.2%	Gastrointestinal Symptoms	9.6%
Neurological Symptoms	7.2%	Neurological Symptoms	7.1%
Mental health	4.1%	Mental health	5.0%
Allergic Symptoms	3.5%	Sleep Issues	4.0%
Sleep Issues	3.3%	Allergic Symptoms	3.1%
Other	2.8%	Other	2.8%
Gynecological Symptoms	1.7%	Gynecological Symptoms	2.2%
Neurodiversity	0.9%	Neurodiversity	1.3%
Urinary Symptoms	0.9%	Urinary Symptoms	1.1%
Dental Manifestations	0.6%	Dental Manifestations	0.9%
Vision Dysfunction	0.5%	Vision Dysfunction	0.7%
Dermatological Symptoms	0.4%	Endocrine Dysfunction	0.7%
Endocrine Dysfunction	0.4%	Dermatological Symptoms	0.5%

Participants ranked their three most severe symptom domains from a predefined list. Percentages reflect weighted rankings based on participant responses. hEDS n = 3360; HSD n = 546.

**Table 3:** Medical care access and burden in hEDS and HSD participants.

Medical Care	hEDS n (%)	HSD n (%)	hEDS vs. HSD RR (95% CI), *p*-Value †
Average hours per week coordinating care			
Less than 5 h	1741 (51.8)	365 (66.8)	0.78 (0.72–0.83), <0.0001
5 to 10 h	1068 (31.8)	136 (24.9)	1.28 (1.09–1.49), ns
11 to 15 h	305 (9.1)	32 (5.9)	1.55 (1.09–2.20), ns
16 to 20 h	121 (3.6)	8 (1.5)	2.46 (1.21–5.00), ns
More than 20 h	125 (3.7)	5 (0.9)	4.06 (1.67–9.89), ns
Care Coordination			
Self	3092 (92)	494 (90.5)	1.02 (0.99–1.05), ns
Medical provider	553 (16.5)	83 (15.2)	1.08 (0.88–1.34), ns
Family member	417 (12.4)	63 (11.5)	1.08 (0.84–1.38), ns
Other	19 (0.6)	1 (0.2)	-
Medical specialists seen in last year ^‡^	6.5 (±3.7)	5.2 (±3.2)	(−1.64 to −1.04), <0.0001
Medical specialists seen in the past year			
Family medicine	2150 (64)	287 (52.6)	1.22 (1.12–1.32), 0.0002
Cardiology	1918 (57.1)	221 (40.5)	1.41 (1.27–1.57), <0.0001
Gastroenterology	1500 (44.6)	155 (28.4)	1.57 (1.37–1.81), <0.0001
Neurology	1418 (42.2)	172 (31.5)	1.34 (1.18–1.53), 0.0012
Emergency medicine	1383 (41.2)	140 (25.6)	1.61 (1.38–1.86), <0.0001
Psychiatry	1287 (38.3)	190 (34.8)	1.10 (0.97–1.24), ns
Rheumatology	1284 (38.2)	205 (37.5)	1.02 (0.91–1.14), ns
Dermatology	1129 (33.6)	139 (25.5)	1.32 (1.13–1.54), ns
Ophthalmology	1091 (32.5)	120 (22)	1.48 (1.25–1.74), 0.0005
Radiology	1089 (32.4)	110 (20.1)	1.61 (1.35–1.91), <0.0001
Internal medicine	956 (28.5)	115 (21.1)	1.35 (1.14–1.60), ns
Sports medicine	870 (25.9)	140 (25.6)	1.01 (0.87–1.18), ns
Immunology	673 (20)	78 (14.3)	1.40 (1.13–1.74), ns
Endocrinology	596 (17.7)	94 (17.2)	1.03 (0.85–1.26), ns
Urology	507 (15.1)	39 (7.1)	2.11 (1.54–2.89), 0.0004
Otolaryngology	241 (7.2)	36 (6.6)	1.09 (0.78–1.53), ns
Nephrology	118 (3.5)	11 (2)	1.74 (0.95–3.21), ns
Hepatology	81 (2.4)	5 (0.9)	2.63 (1.07–6.47), ns
Geriatric	26 (0.8)	1 (0.2)	-
Other	1503 (44.7)	232 (42.5)	1.05 (0.95–1.17), ns
None of the above	71 (2.1)	19 (3.5)	0.61 (0.37–1.00), ns
Admitted inpatient for hEDS/HSD or related condition	1395 (41.5)	108 (19.8)	2.10 (1.76–2.50), <0.0001
Admissions in the last year			
0	581 (17.3)	47 (8.6)	2.01 (1.51–2.67), 0.0002
1	403 (12)	27 (4.9)	2.43 (1.66–3.54), 0.0006
2	185 (5.5)	21 (3.8)	1.43 (0.92–2.23), ns
3+	226 (6.7)	13 (2.4)	2.83 (1.63–4.90), ns
N/A	1965 (58.5)	438 (80.2)	-
Farthest distance traveled for treatment			
Less than 300 miles	2309 (68.7)	458 (83.9)	0.82 (0.78–0.86), <0.0001
300 to 599 miles	503 (15)	46 (8.4)	1.78 (1.33–2.37), 0.0249
600 to 999 miles	219 (6.5)	13 (2.4)	2.74 (1.58–4.75), ns
1000 to 2000 miles	169 (5)	11 (2)	2.50 (1.37–4.56), ns
More than 2000 miles	160 (4.8)	18 (3.3)	1.44 (0.89–2.33), ns
Paid out-of-pocket for medical expenses	2041 (60.7)	245 (44.9)	1.35 (1.23–1.49), 0.0103

Visits, inpatient admissions, travel distance, and out-of-pocket expenses. hEDS n = 3360; HSD n = 546. † *p* values were calculated using Chi-square tests, with Bonferroni correction applied to adjust for multiple comparisons. ‡ Mann–Whitney U test on data cleaned using ROUT (Q = 1%). - indicates the response was excluded from analysis. ns—not significant.

**Table 4:** Prevalence of chronic pain and pain locations in hEDS and HSD.

Chronic Pain	hEDS n (%)	HSD n (%)	hEDS vs. HSD RR (95% CI), *p*-Value †
Experience chronic pain	3318 (98.8)	506 (92.7)	1.07 (1.04–1.09), <0.0001
Chronic Pain Sites			
Neck	2684 (79.9)	367 (67.2)	1.19 (1.12–1.26), <0.0001
Lower back	2616 (77.9)	359 (65.8)	1.18 (1.11–1.26), <0.0001
Shoulder	2578 (76.7)	360 (65.9)	1.16 (1.09–1.24), <0.0001
Hip	2523 (75.1)	342 (62.6)	1.20 (1.12–1.28), <0.0001
Knee	2397 (71.3)	324 (59.3)	1.20 (1.12–1.29), <0.0001
Fingers	2162 (64.3)	276 (50.5)	1.27 (1.17–1.39), <0.0001
Upper back	2132 (63.5)	291 (53.3)	1.19 (1.10–1.29), 0.0030
Wrist	1959 (58.3)	269 (49.3)	1.18 (1.08–1.29), 0.0387
Head	1894 (56.4)	219 (40.1)	1.41 (1.26–1.56), <0.0001
Jaw	1880 (56)	233 (42.7)	1.31 (1.18–1.45), <0.0001
Ankle	1861 (55.4)	245 (44.9)	1.23 (1.12–1.36), 0.0025
Widespread	1842 (54.8)	232 (42.5)	1.29 (1.16–1.43), <0.0001
Abdominal	1774 (52.8)	188 (34.4)	1.53 (1.36–1.73), <0.0001
Ribs	1479 (44)	153 (28)	1.57 (1.37–1.81), <0.0001
Elbow	1149 (34.2)	145 (26.6)	1.29 (1.11–1.49), ns
Toes	1079 (32.1)	136 (24.9)	1.29 (1.11–1.50), ns
Other	283 (8.4)	47 (8.6)	0.98 (0.73–1.31), ns

hEDS n = 3360; HSD n = 546; ns, not significant. † *p* values were calculated using Chi-square tests, with Bonferroni correction applied to adjust for multiple comparisons.

**Table 5:** Alternative medicine modalities tried by hEDS and HSD participants.

Alternative Medicine	hEDS n (%)	HSD n (%)
Tried alternative medicine	2858 (85.1)	445 (81.5)
Alternative medicine type		
Massage	2366 (70.4)	369 (67.6)
Chiropractic	1846 (54.9)	250 (45.8)
Meditation	1553 (46.2)	218 (39.9)
Acupuncture	1491 (44.4)	232 (42.5)
Functional medicine	745 (22.2)	99 (18.1)
Naturopathy	632 (18.8)	77 (14.1)
Homeopathy	596 (17.7)	76 (13.9)
Chinese medicine	423 (12.6)	69 (12.6)
Hypnosis	220 (6.5)	35 (6.4)
Ayurveda	167 (5)	19 (3.5)
Other	239 (7.1)	44 (8.1)

hEDS n = 3360; HSD n = 546.

**Table 6:** Anesthesia-related complications and reactions in hEDS and HSD.

Anesthesia Complications	hEDS n (%)	HSD n (%)	hEDS vs. HSD RR (95% CI), *p*-Value †
Anesthesia Complications	1660 (49.4)	155 (28.4)	1.74 (1.52–2.00), <0.0001
Anesthesia type			
Local anesthesia only	1287 (38.3)	105 (19.2)	1.99 (1.67–2.38), ns
General anesthesia only	1191 (35.4)	95 (17.4)	2.04 (1.69–2.46), ns
Both general and local anesthesia	844 (25.1)	47 (8.6)	2.92 (2.21–3.86), 0.0007
Complication Type			
Shortened effect	1270 (37.8)	103 (18.9)	2.00 (1.67–2.40), ns
Insufficient pain control	1017 (30.3)	62 (11.4)	2.67 (2.10–3.39), 0.0002
Intubation complication	193 (5.7)	15 (2.7)	2.09 (1.25–3.51), ns
Anaphylaxis or allergic reaction	137 (4.1)	8 (1.5)	2.78 (1.37–5.64), ns
Other	400 (11.9)	46 (8.4)	1.41 (1.06–1.89), ns

hEDS n = 3360; HSD n = 546; ns, not significant. † *p* values were calculated using Chi-square tests, with Bonferroni correction applied to adjust for multiple comparisons.

**Table 7:** Prevalence of orthopedic diagnoses in hEDS, HSD, and All of Us.

Orthopedic Disorders	hEDS n (%)	HSD n (%)	hEDS vs. HSD RR (95% CI), *p*-Value †	All of Us n (%)	hEDS vs. All of Us RR (95% CI), *p*-Value †
Tendonitis	1631 (48.5)	215 (39.4)	1.23 (1.10–1.38), 0.0352	23,000 (6.5)	7.48 (7.21–7.76), <0.0001
Bursitis	1199 (35.7)	157 (28.8)	1.24 (1.08–1.43), ns	29,060 (8.2)	4.35 (4.15–4.56), <0.0001
Ligament tear	1090 (32.4)	127 (23.3)	1.39 (1.19–1.64), 0.0091	61,560 (17.4)	1.87 (1.78–1.96), <0.0001
Tendon rupture	543 (16.2)	63 (11.5)	1.40 (1.10–1.79), ns	1320 (0.4)	43.39 (39.50–47.66), <0.0001
Slipping rib syndrome	383 (11.4)	22 (4)	2.83 (1.86–4.31), 0.0001	7040 (2)	5.74 (5.21–6.32), <0.0001
Congenital hip dislocation	161 (4.8)	12 (2.2)	2.18 (1.22–3.89), ns	100 (0)	169.82 (132.62–217.45), <0.0001
Bowing of legs	159 (4.7)	17 (3.1)	1.52 (0.93–2.49), ns	940 (0.3)	17.84 (15.13–21.03), <0.0001
Osgood-Schlatter disease	155 (4.6)	19 (3.5)	1.33 (0.83–2.12), ns	-	-
Pectus excavatum	125 (3.7)	10 (1.8)	2.03 (1.07–3.84), ns	340 (0.1)	38.78 (31.68–47.47), <0.0001
Joint contracture	70 (2.1)	7 (1.3)	1.63 (0.75–3.52), ns	180 (0.1)	41.02 (31.19–53.95), <0.0001
Club foot	70 (2.1)	5 (0.9)	2.28 (0.92–5.61), ns	40 (0)	184.58 (125.35–271.81), <0.0001
Congenital muscle hypotonia	42 (1.3)	3 (0.5)	-	-	-
None of the above	885 (26.3)	205 (37.5)	0.70 (0.62–0.79), <0.0001	-	-

hEDS n = 3360; HSD n = 546; All of Us n = 354,400; ns, not significant. † *p* values were calculated using Chi-square tests, with Bonferroni correction applied to adjust for multiple comparisons. - indicates the response was excluded from analysis.

**Table 8:** Orthopedic symptoms and joint sites impacted in hEDS and HSD.

Orthopedic Symptoms	hEDS n (%)	HSD n (%)	hEDS vs. HSD RR (95% CI), *p*-Value †
Joint subluxations	3254 (96.8)	467 (85.5)	1.13 (1.09–1.17), <0.0001
Joint dislocations	2159 (64.3)	196 (35.9)	1.79 (1.60–2.01), <0.0001
Unexplained fractures	666 (19.8)	52 (9.5)	2.08 (1.59–2.72), <0.0001
None of the above	75 (2.2)	64 (11.7)	0.19 (0.14–0.26), <0.0001
Joints Affected			
Shoulder	2704 (80.5)	328 (60.1)	1.34 (1.25–1.44), <0.0001
Hip	2428 (72.3)	256 (46.9)	1.54 (1.41–1.69), <0.0001
Knee	2345 (69.8)	275 (50.4)	1.39 (1.27–1.51), <0.0001
Fingers	2313 (68.8)	258 (47.3)	1.46 (1.33–1.60), <0.0001
Ribs	2022 (60.2)	198 (36.3)	1.66 (1.48–1.86), <0.0001
Ankle	2003 (59.6)	221 (40.5)	1.47 (1.33–1.64), <0.0001
Wrist	1872 (55.7)	180 (33)	1.69 (1.49–1.91), <0.0001
Toes	1387 (41.3)	124 (22.7)	1.82 (1.55–2.13), <0.0001
Elbow	1315 (39.1)	139 (25.5)	1.54 (1.32–1.79), <0.0001
Spine	1288 (38.3)	129 (23.6)	1.62 (1.39–1.90), <0.0001
Other	454 (13.5)	47 (8.6)	1.57 (1.18–2.09), ns

hEDS n = 3360; HSD n = 546; ns, not significant. † *p* values were calculated using Chi-square tests, with Bonferroni correction applied to adjust for multiple comparisons.

**Table 9:** Reported anatomical abnormalities in hEDS participants.

Anatomical Abnormalities	hEDS n = 3360 n (%)
Uterus	549 (16.3)
Ribs	40 (1.2)
Lumbar/sacral	38 (1.1)
Extra or missing vertebrae	9 (0.3)

**Table 10:** Prevalence of neurological diagnoses in hEDS, HSD, and All of Us.

Neurological Disorders	hEDS n (%)	HSD n (%)	hEDS vs. HSD RR (95% CI), *p*-Value †	All of Us n (%)	hEDS vs. All of Us RR (95% CI), *p*-Value †
Migraine	2162 (64.3)	255 (46.7)	1.38 (1.26–1.51), <0.0001	49,740 (14)	4.58 (4.46–4.71), <0.0001
Scoliosis	1145 (34.1)	114 (20.9)	1.63 (1.38–1.93), <0.0001	11,680 (3.3)	10.34 (9.83–10.87), <0.0001
Raynaud’s phenomenon	1130 (33.6)	104 (19)	1.77 (1.48–2.11), <0.0001	3060 (0.9)	38.95 (36.71–41.32), <0.0001
Tinnitus	1046 (31.1)	121 (22.2)	1.40 (1.19–1.66), 0.0113	26,620 (7.5)	4.14 (3.94–4.36), <0.0001
Other nerve entrapments	910 (27.1)	100 (18.3)	1.48 (1.23–1.78), 0.0076	-	-
Herniated disk(s)	882 (26.3)	97 (17.8)	1.48 (1.22–1.79), 0.0117	37,360 (10.5)	2.49 (2.35–2.64), <0.0001
CCI/AAI	742 (22.1)	41 (7.5)	2.94 (2.18–3.97), <0.0001	180 (0.1)	434.80 (370.78–509.86), <0.0001
ME/CFS	568 (16.9)	78 (14.3)	1.18 (0.95–1.47), ns	12,920 (3.6)	4.64 (4.29–5.01), <0.0001
Spinal stenosis	443 (13.2)	36 (6.6)	2.00 (1.44–2.77), 0.0077	37,140 (10.5)	1.26 (1.15–1.37), <0.0001
Other spinal instability	396 (11.8)	42 (7.7)	1.53 (1.13–2.08), ns	15,640 (4.4)	2.67 (2.43–2.93), <0.0001
Occipital neuralgia	394 (11.7)	23 (4.2)	2.78 (1.85–4.20), <0.0001	3160 (0.9)	13.15 (11.91–14.52), <0.0001
SFN	337 (10)	24 (4.4)	2.28 (1.52–3.42), 0.0148	80 (0)	444.32 (349.03–565.62), <0.0001
Thoracic outlet syndrome	292 (8.7)	30 (5.5)	1.58 (1.10–2.28), ns	160 (0)	192.49 (159.22–232.72), <0.0001
Hearing impairment	291 (8.7)	33 (6)	1.43 (1.01–2.03), ns	52,120 (14.7)	0.59 (0.53–0.66), <0.0001
CRPS	236 (7)	17 (3.1)	2.26 (1.39–3.66), ns	1660 (0.5)	15.00 (13.14–17.11), <0.0001
Trigeminal neuralgia	228 (6.8)	18 (3.3)	2.06 (1.28–3.30), ns	2360 (0.7)	10.19 (8.93–11.62), <0.0001
Chiari malformation	211 (6.3)	5 (0.9)	6.86 (2.84–16.57), 0.0003	360 (0.1)	61.82 (52.34–73.02), <0.0001
Dystonia	208 (6.2)	16 (2.9)	2.11 (1.28–3.48), ns	3080 (0.9)	7.12 (6.22–8.16), <0.0001
Kyphoscoliosis	199 (5.9)	9 (1.6)	3.59 (1.85–6.96), 0.0240	240 (0.1)	87.46 (72.70–105.21), <0.0001
CSF leak	177 (5.3)	13 (2.4)	2.21 (1.27–3.86), ns	60 (0)	311.15 (232.63–416.18), <0.0001
TCS	154 (4.6)	5 (0.9)	5.01 (2.06–12.14), 0.0393	40 (0)	406.08 (287.26–574.05), <0.0001
Voice disorder	134 (4)	6 (1.1)	3.63 (1.61–8.18), ns	11,320 (3.2)	1.25 (1.06–1.48), ns
Intracranial hypotension	126 (3.8)	9 (1.6)	2.28 (1.16–4.45), ns	-	-
Syringomyelia	53 (1.6)	3 (0.5)	-	-	-
Transverse sinus stenosis	24 (0.7)	5 (0.9)	0.78 (0.30–2.04), ns	-	-
Multiple sclerosis	23 (0.7)	0 (0)	-	-	-
Myasthenia gravis	14 (0.4)	0 (0)	-	-	-
None of the above	341 (10.1)	131 (24)	0.42 (0.35–0.51), <0.0001	-	-

hEDS n = 3360; HSD n = 546; All of Us n = 354,400; ns, not significant; CCI, cranio-cervical instability; AAI, atlantoaxial instability; ME/CFS, myalgic encephalomyelitis/chronic fatigue syndrome; SFN, small-fiber neuropathy; CRPS, complex regional pain syndrome; CSF, cerebrospinal fluid; TCS, tethered cord syndrome. † *p* values were calculated using Chi-square tests, with Bonferroni correction applied to adjust for multiple comparisons. - indicates the response was excluded from analysis.

**Table 11:** Prevalence of neurological symptoms in hEDS and HSD.

Neurological Symptoms	hEDS n (%)	HSD n (%)	hEDS vs. HSD RR (95% CI), *p*-Value †
Weakness	2676 (79.6)	391 (71.6)	1.11 (1.05–1.18), 0.0121
Muscle spasm	2632 (78.3)	367 (67.2)	1.17 (1.10–1.24), <0.0001
Tingling	2555 (76)	366 (67)	1.13 (1.07–1.21), 0.0037
Numbness	2365 (70.4)	288 (52.7)	1.33 (1.23–1.45), <0.0001
None of the above	122 (3.6)	37 (6.8)	0.54 (0.38–0.77), ns

hEDS n = 3360; HSD n = 546; ns, not significant. † *p* values were calculated using Chi-square tests, with Bonferroni correction applied to adjust for multiple comparisons.

**Table 12:** Prevalence of autonomic disorders in hEDS, HSD, and All of Us.

Autonomic Disorders	hEDS n (%)	HSD n (%)	hEDS vs. HSD RR (95% CI), *p*-Value †	All of Us n (%)	hEDS vs. All of Us RR (95% CI), *p*-Value †
POTS	1772 (52.7)	142 (26)	2.03 (1.75–2.34), <0.0001	660 (0.2)	283.19 (260.72–307.59), <0.0001
General dysautonomia	906 (27)	80 (14.7)	1.84 (1.49–2.27), <0.0001	5080 (1.4)	18.81 (17.68–20.01), <0.0001
Orthostatic hypotension	545 (16.2)	44 (8.1)	2.01 (1.50–2.70), 0.0004	10,840 (3.1)	5.30 (4.90–5.74), <0.0001
Orthostatic intolerance	512 (15.2)	43 (7.9)	1.93 (1.44–2.61), 0.0028	-	-
Autonomic neuropathy	247 (7.4)	13 (2.4)	3.09 (1.78–5.35), 0.0098	2520 (0.7)	10.34 (9.11–11.73), <0.0001
Hyperadrenergic POTS	178 (5.3)	14 (2.6)	2.07 (1.21–3.53), ns	-	-
Hypovolemic POTS	82 (2.4)	7 (1.3)	1.90 (0.88–4.10), ns	6500 (1.8)	1.33 (1.07–1.65), <0.0001
Pure autonomic failure	14 (0.4)	0 (0)	-	-	-
None of the above	962 (28.6)	326 (59.7)	0.48 (0.44–0.52), <0.0001	-	-

hEDS n = 3360; HSD n = 546; All of Us n = 354,400; ns, not significant; POTS, postural orthostatic tachycardia syndrome. † *p* values were calculated using Chi-square tests, with Bonferroni correction applied to adjust for multiple comparisons. - indicates the response was excluded from analysis.

**Table 13:** Prevalence of autonomic symptoms in hEDS and HSD.

Autonomic Symptoms	hEDS n (%)	HSD n (%)	hEDS vs. HSD RR (95% CI), *p*-Value †
Fatigue	3312 (98.6)	516 (94.5)	1.04 (1.02–1.06), <0.0001
Dizziness	3216 (95.7)	482 (88.3)	1.08 (1.05–1.12), <0.0001
Brain fog	3211 (95.6)	491 (89.9)	1.06 (1.03–1.09), <0.0001
Heart palpitations	3177 (94.6)	468 (85.7)	1.10 (1.06–1.14), <0.0001
Thermoregulatory dysfunction	3090 (92)	440 (80.6)	1.14 (1.09–1.19), <0.0001
Vertigo	2782 (82.8)	371 (67.9)	1.22 (1.15–1.29), <0.0001
Trouble swallowing	2171 (64.6)	251 (46)	1.41 (1.28–1.54), <0.0001
Fainting	2166 (64.5)	239 (43.8)	1.47 (1.33–1.62), <0.0001
None of the above	3 (0.1)	6 (1.1)	-

hEDS n = 3360; HSD n = 546. † *p* values were calculated using Chi-square tests, with Bonferroni correction applied to adjust for multiple comparisons. - indicates the response was excluded from analysis.

**Table 14:** Prevalence of gastrointestinal diagnoses in hEDS, HSD, and All of Us.

Gastrointestinal Disorders	hEDS n (%)	HSD n (%)	hEDS vs. HSD RR (95% CI), *p*-Value †	All of Us n (%)	hEDS vs. All of Us RR (95% CI), *p*-Value †
Irritable bowel syndrome (IBS)	1627 (48.4)	201 (36.8)	1.32 (1.17–1.48), 0.0002	21,220 (6)	8.09 (7.79–8.39), <0.0001
Gastroesophageal reflux disease (GERD)	1601 (47.6)	168 (30.8)	1.55 (1.36–1.76), <0.0001	110,180 (31.1)	1.53 (1.48–1.59), <0.0001
Constipation	1467 (43.7)	153 (28)	1.56 (1.35–1.79), <0.0001	63,800 (18)	2.43 (2.33–2.52), <0.0001
Dysmotility	880 (26.2)	57 (10.4)	2.51 (1.95–3.23), <0.0001	-	-
Gastritis	818 (24.3)	81 (14.8)	1.64 (1.33–2.02), 0.0005	35,320 (10)	2.44 (2.30–2.59), <0.0001
Obesity	730 (21.7)	106 (19.4)	1.12 (0.93–1.34), ns	111,560 (31.5)	0.69 (0.65–0.74), <0.0001
Non-celiac gluten sensitivity	514 (15.3)	60 (11)	1.39 (1.08–1.79), ns	-	-
Hiatal hernia	501 (14.9)	43 (7.9)	1.89 (1.41–2.55), 0.0060	700 (0.2)	75.49 (67.66–84.23), <0.0001
Abdominal hernia	341 (10.1)	18 (3.3)	3.08 (1.93–4.90), 0.0002	48,640 (13.7)	0.74 (0.67–0.82), <0.0001
Gastric ulcer	330 (9.8)	18 (3.3)	2.98 (1.87–4.75), 0.0004	7420 (2.1)	4.69 (4.22–5.21), <0.0001
Appendicitis	322 (9.6)	45 (8.2)	1.16 (0.86–1.57), ns	4200 (1.2)	8.09 (7.26–9.01), <0.0001
Small intestine bacterial overgrowth (SIBO)	312 (9.3)	31 (5.7)	1.64 (1.14–2.34), ns	-	-
Colorectal polyp(s)	283 (8.4)	30 (5.5)	1.53 (1.06–2.21), ns	26,580 (7.5)	1.12 (1.00–1.26), ns
Diverticulosis/diverticulitis	262 (7.8)	18 (3.3)	2.37 (1.48–3.78), ns	9320 (2.6)	2.97 (2.64–3.34), <0.0001
Functional dyspepsia	190 (5.7)	17 (3.1)	1.82 (1.12–2.96), ns	4860 (1.4)	4.12 (3.58–4.75), <0.0001
Celiac disease	150 (4.5)	14 (2.6)	1.74 (1.01–2.99), ns	2760 (0.8)	5.73 (4.88–6.73), <0.0001
Feeding intolerance	125 (3.7)	8 (1.5)	2.54 (1.25–5.16), ns	1360 (0.4)	9.69 (8.10–11.61), <0.0001
Cholelithiasis	118 (3.5)	9 (1.6)	2.13 (1.09–4.17), ns	22,980 (6.5)	0.54 (0.45–0.65), <0.0001
Liver disease	105 (3.1)	10 (1.8)	1.71 (0.90–3.24), ns	60,580 (17.1)	0.18 (0.15–0.22), <0.0001
Eosinophilic esophagitis (EoE)	102 (3)	10 (1.8)	1.66 (0.87–3.15), ns	1300 (0.4)	8.28 (6.78–10.09), <0.0001
Median arcuate ligament syndrome (MALS)	96 (2.9)	4 (0.7)	-	-	-
Duodenal ulcer	78 (2.3)	3 (0.5)	-	-	-
Superior mesenteric artery syndrome (SMAS)	62 (1.8)	2 (0.4)	-	-	-
Ulcerative colitis	59 (1.8)	6 (1.1)	1.60 (0.69–3.68), ns	4060 (1.1)	1.53 (1.19–1.98), ns
Crohn’s disease	43 (1.3)	5 (0.9)	1.40 (0.56–3.51), ns	4040 (1.1)	1.12 (0.83–1.51), <0.0001
Visceroptosis	26 (0.8)	1 (0.2)	-	-	-
Intestinal malrotation	22 (0.7)	1 (0.2)	-	-	-
Gastrointestinal organ rupture	17 (0.5)	1 (0.2)	-	-	-
None of the above	529 (15.7)	169 (31)	0.51 (0.44–0.59), ns	-	-
Types of dysmotility					
Gastric	711 (21.2)	43 (7.9)	2.69 (2.00–3.61), <0.0001	5260 (1.5)	14.26 (13.29–15.30), <0.0001
Esophageal	232 (6.9)	13 (2.4)	2.90 (1.67–5.03), 0.0329	-	-
Colon	230 (6.8)	17 (3.1)	2.20 (1.35–3.57), ns	4000 (1.1)	6.06 (5.33–6.90), <0.0001
Small bowel	202 (6)	8 (1.5)	4.10 (2.04–8.27), 0.0083	-	-
Types of IBS					
Mixed	911 (27.1)	89 (16.3)	1.66 (1.36–2.03), <0.0001	-	-
Constipation	435 (12.9)	46 (8.4)	1.54 (1.15–2.05), ns	-	-
Diarrhea	357 (10.6)	48 (8.8)	1.21 (0.91–1.61), ns	-	-
Undifferentiated	233 (6.9)	42 (7.7)	0.90 (0.66–1.24), ns	-	-

hEDS n = 3360; HSD n = 546; All of Us n = 354,400; ns, not significant; IBS, irritable bowel syndrome; GERD, gastroesophageal reflux disease; SIBO, small intestinal bacterial overgrowth; EoE, eosinophilic esophagitis; MALS, median arcuate ligament syndrome; SMAS, superior mesenteric artery syndrome. † *p* values were calculated using Chi-square tests, with Bonferroni correction applied to adjust for multiple comparisons. - indicates the response was excluded from analysis.

**Table 15:** Prevalence of gastrointestinal symptoms in hEDS and HSD.

Gastrointestinal Symptoms	hEDS n (%)	HSD n (%)	hEDS vs. HSD RR (95% CI), *p*-Value †
Abdominal pain	3120 (92.9)	439 (80.4)	1.15 (1.11–1.20), <0.0001
Nausea	3044 (90.6)	425 (77.8)	1.16 (1.11–1.22), <0.0001
Bloating	3012 (89.6)	439 (80.4)	1.11 (1.07–1.16), <0.0001
Constipation	2925 (87.1)	412 (75.5)	1.15 (1.10–1.21), <0.0001
Diarrhea	2778 (82.7)	391 (71.6)	1.15 (1.09–1.22), <0.0001
Reflux	2640 (78.6)	350 (64.1)	1.23 (1.15–1.31), <0.0001
Heartburn	2488 (74)	337 (61.7)	1.20 (1.12–1.29), <0.0001
Vomiting	1968 (58.6)	211 (38.6)	1.52 (1.36–1.69), <0.0001
Rectal bleeding	1523 (45.3)	166 (30.4)	1.49 (1.31–1.70), <0.0001
None of the above	20 (0.6)	8 (1.5)	0.41 (0.18–0.92), ns

hEDS n = 3360; HSD n = 546; ns, not significant. † *p* values were calculated using Chi-square tests, with Bonferroni correction applied to adjust for multiple comparisons.

**Table 16:** Use of feeding devices and ostomies in hEDS and HSD.

Feeding Devices and Ostomies	hEDS n (%)	HSD n (%)	hEDS vs. HSD RR (95% CI), *p*-Value †
Have used a feeding device	240 (7.1)	11 (2)	3.55 (1.95–6.44), 0.0038
Type of Feeding Device			
Nasogastric (NG) tube	130 (3.9)	4 (0.7)	-
Nasojejunal (NJ) tube	110 (3.3)	4 (0.7)	-
Gastrojejunostomy (GJ) tube	74 (2.2)	3 (0.5)	-
Total parenteral nutrition (TPN)	74 (2.2)	2 (0.4)	-
Jejunostomy (JEJ, PEJ, or RIJ) tube	55 (1.6)	0 (0)	-
Gastric (G) tube	48 (1.4)	3 (0.5)	-
Peripheral parenteral nutrition (PPN)	28 (0.8)	0 (0)	-
Nasoduodenal (ND) tube	26 (0.8)	1 (0.2)	-
Orogastric (OG) tube	3 (0.1)	0 (0)	-
Oroenteric tube	0 (0)	0 (0)	-
Ostomies			
Colostomy	51 (1.5)	10 (1.8)	-
Ileostomy	38 (1.1)	2 (0.4)	-
None of the above	3287 (97.8)	535 (98)	-

hEDS n = 3360; HSD n = 546. † *p* values were calculated using Chi-square tests, with Bonferroni correction applied to adjust for multiple comparisons. - indicates the response was excluded from analysis.

**Table 17:** Prevalence of cardiopulmonary diagnoses in hEDS, HSD, and All of Us.

Cardiopulmonary Disorders	hEDS n (%)	HSD n (%)	hEDS vs. HSD RR (95% CI), *p*-Value †	All of Us n (%)	hEDS vs. All of Us RR (95% CI), *p*-Value †
Mitral valve defect	547 (16.3)	30 (5.5)	2.96 (2.07–4.23), <0.0001	2880 (0.8)	20.03 (18.40–21.81), <0.0001
Other arrhythmia	485 (14.4)	53 (9.7)	1.49 (1.14–1.95), ns	-	-
Supraventricular tachycardia (SVT)	263 (7.8)	20 (3.7)	2.14 (1.37–3.34), ns	32,080 (9.1)	0.86 (0.77–0.97), ns
Tricuspid valve defect	201 (6)	13 (2.4)	2.51 (1.44–4.37), ns	15,340 (4.3)	1.38 (1.21–1.58), 0.0005
Aortic valve defect	111 (3.3)	3 (0.5)	-	-	-
Lung disease	99 (2.9)	11 (2)	1.46 (0.79–2.71), ns	83,600 (23.6)	0.12 (0.10–0.15), <0.0001
Atrial fibrillation	99 (2.9)	7 (1.3)	2.30 (1.07–4.92), ns	26,160 (7.4)	0.40 (0.33–0.48), <0.0001
Stroke	80 (2.4)	5 (0.9)	2.60 (1.06–6.39), ns	160 (0)	52.74 (40.41–68.82), <0.0001
May-Thurner syndrome	62 (1.8)	6 (1.1)	1.68 (0.73–3.86), ns	-	-
Nutcracker syndrome	61 (1.8)	4 (0.7)	-	-	-
Pulmonary valve defect	60 (1.8)	3 (0.5)	-	-	-
Patent foramen ovale (PFO)	51 (1.5)	4 (0.7)	-	-	-
Heart failure	44 (1.3)	2 (0.4)	-	-	-
Coronary artery disease	36 (1.1)	2 (0.4)	-	-	-
Aortic aneurysm	32 (1)	0 (0)	-	-	-
Atrial septal defect (ASD) (not PFO)	24 (0.7)	1 (0.2)	-	-	-
Myocardial infarction	17 (0.5)	2 (0.4)	-	-	-
Hypertrophic cardiomyopathy	15 (0.4)	2 (0.4)	-	-	-
Rheumatic heart disease	5 (0.1)	0 (0)	-	-	-
Spontaneous Coronary Artery Dissection (SCAD)	4 (0.1)	0 (0)	-	-	-
Ebstein’s anomaly	2 (0.1)	1 (0.2)	-	-	-
None of the above	2083 (62)	431 (78.9)	0.79 (0.75–0.83), <0.0001	-	-

hEDS n = 3360; HSD n = 546; All of Us n = 354,400; ns, not significant; SVT, supraventricular tachycardia; PFO, patent foramen ovale; ASD, atrial septal defect; SCAD, spontaneous coronary artery dissection. † *p* values were calculated using Chi-square tests, with Bonferroni correction applied to adjust for multiple comparisons. - indicates the response was excluded from analysis.

**Table 18:** Prevalence of endocrinological diagnoses in hEDS, HSD, and All of Us.

Endocrinological Disorders	hEDS n (%)	HSD n (%)	hEDS vs. HSD RR (95% CI), *p*-Value †	All of Us n (%)	hEDS vs. All of Us RR (95% CI), *p*-Value †
Hypothyroidism (non-autoimmune)	389 (11.6)	49 (9)	1.29 (0.97–1.71), ns	36,920 (10.4)	1.11 (1.01–1.22), ns
Osteopenia	354 (10.5)	42 (7.7)	1.37 (1.01–1.86), ns	3220 (0.9)	11.60 (10.45–12.87), <0.0001
Hashimoto’s disease	231 (6.9)	30 (5.5)	1.25 (0.86–1.81), ns	2880 (0.8)	8.46 (7.43–9.63), <0.0001
Osteoporosis	189 (5.6)	18 (3.3)	1.71 (1.06–2.74), ns	32,160 (9.1)	0.62 (0.54–0.71), <0.0001
Adrenal insufficiency (non-autoimmune)	104 (3.1)	18 (3.3)	0.94 (0.57–1.54), ns	2240 (0.6)	4.90 (4.03–5.94), <0.0001
Hyperthyroidism (non-autoimmune)	83 (2.5)	6 (1.1)	2.25 (0.99–5.12), ns	740 (0.2)	11.83 (9.45–14.81), <0.0001
Diabetes, type 2	82 (2.4)	14 (2.6)	0.95 (0.54–1.67), ns	74,920 (21.1)	0.12 (0.09–0.14), <0.0001
Diabetes, gestational	77 (2.3)	7 (1.3)	1.79 (0.83–3.85), ns	3180 (0.9)	2.55 (2.04–3.19), <0.0001
Pineal cyst	62 (1.8)	5 (0.9)	2.02 (0.81–4.99), ns	40 (0)	163.49 (110.02–242.93), <0.0001
Pituitary tumor/adenoma	56 (1.7)	6 (1.1)	1.52 (0.66–3.50), ns	200 (0.1)	29.53 (22.00–39.64), <0.0001
Grave’s disease	33 (1)	6 (1.1)	0.89 (0.38–2.12), ns	300 (0.1)	11.60 (8.11–16.59), <0.0001
Hyperparathyroidism	27 (0.8)	5 (0.9)	0.88 (0.34–2.27), ns	10,560 (3)	0.27 (0.19–0.39), <0.0001
Diabetes, type 1	23 (0.7)	1 (0.2)	-	-	-
Cushing disease	22 (0.7)	1 (0.2)	-	-	-
Addison’s disease	12 (0.4)	1 (0.2)	-	-	-
Congenital adrenal hyperplasia	9 (0.3)	1 (0.2)	-	-	-
Multiple endocrine neoplasia	2 (0.1)	0 (0)	-	-	-
None of the above	2485 (74)	435 (79.7)	-	-	-

hEDS n = 3360; HSD n = 546; All of Us n = 354,400; ns, not significant. † *p* values were calculated using Chi-square tests, with Bonferroni correction applied to adjust for multiple comparisons. - indicates the response was excluded from analysis.

**Table 19:** Prevalence of hematological diagnoses and bruising in hEDS, HSD, and All of Us.

Hematological Disorders	hEDS n (%)	HSD n (%)	hEDS vs. HSD RR (95% CI), *p*-Value †	All of Us n (%)	hEDS vs. All of Us RR (95% CI), *p*-Value †
Pernicious anemia	155 (4.6)	14 (2.6)	1.80 (1.05–3.09), ns	1180 (0.3)	13.85 (11.76–16.32), <0.0001
Deep vein thrombosis (DVT)	116 (3.5)	6 (1.1)	3.14 (1.39–7.10), ns	11,440 (3.2)	1.07 (0.89–1.28), ns
Pulmonary embolism (PE)	71 (2.1)	8 (1.5)	1.44 (0.70–2.98), ns	9940 (2.8)	0.75 (0.60–0.95), ns
Lymphedema	63 (1.9)	6 (1.1)	1.71 (0.74–3.92), ns	6560 (1.9)	1.01 (0.79–1.30), ns
Thrombocytosis	41 (1.2)	5 (0.9)	1.33 (0.53–3.36), ns	3700 (1)	1.17 (0.86–1.59), ns
Antiphospholipid antibody syndrome (APLS)	37 (1.1)	1 (0.2)	-	-	-
Von Willebrand disease	33 (1)	4 (0.7)	-	-	-
Hemophilia	15 (0.4)	2 (0.4)	-	-	-
Arterial thrombosis	6 (0.2)	0 (0)	-	-	-
None of the above	2971 (88.4)	510 (93.4)	-	-	-
Easy or severe bruising	2696 (80.2)	337 (61.7)	1.30 (1.21–1.39), <0.0001	-	-

hEDS n = 3360; HSD n = 546; All of Us n = 354,400; ns, not significant; DVT, deep vein thrombosis; PE, pulmonary embolism; APLS, antiphospholipid antibody syndrome. † *p* values were calculated using Chi-square tests, with Bonferroni correction applied to adjust for multiple comparisons. - indicates the response was excluded from analysis.

**Table 20:** Prevalence of reproductive health conditions in hEDS, HSD, and All of Us.

Reproductive Disorders	hEDS n (%)	HSD n (%)	hEDS vs. HSD RR (95% CI), *p*-Value †	All of Us n (%)	hEDS vs. All of Us RR (95% CI), *p*-Value †
Endometriosis	622 (18.5)	66 (12.1)	1.53 (1.21–1.94), ns	7620 (2.2)	8.61 (7.99–9.27), <0.0001
Polycystic ovary syndrome (PCOS)	571 (17)	80 (14.7)	1.16 (0.93–1.44), ns	5000 (1.4)	12.05 (11.12–13.04), <0.0001
Pelvic organ prolapse	455 (13.5)	19 (3.5)	3.89 (2.48–6.10), <0.0001	10,020 (2.8)	4.79 (4.39–5.23), <0.0001
Vaginismus	246 (7.3)	28 (5.1)	1.43 (0.98–2.09), ns	320 (0.1)	81.08 (68.91–95.41), <0.0001
Vulvodynia	231 (6.9)	30 (5.5)	1.25 (0.86–1.81), ns	620 (0.2)	39.30 (33.92–45.53), <0.0001
Infertility	198 (5.9)	36 (6.6)	0.89 (0.63–1.26), ns	7340 (2.1)	2.85 (2.48–3.26), <0.0001
Pelvic congestion syndrome	101 (3)	10 (1.8)	1.64 (0.86–3.12), ns	260 (0.1)	40.97 (32.64–51.43), <0.0001
Hypogonadism	11 (0.3)	3 (0.5)	-	-	-
Erectile dysfunction	10 (0.3)	3 (0.5)	-	-	-
Enlarged prostate gland	7 (0.2)	0 (0)	-	-	-
Premature ejaculation	3 (0.1)	2 (0.4)	-	-	-
Peyronie’s disease	1 (0)	0 (0)	-	-	-
Testicular torsion	1 (0)	0 (0)	-	-	-
Penile fracture	1 (0)	0 (0)	-	-	-
Cryptorchidism	0 (0)	1 (0.2)	-	-	-

hEDS n = 3360; HSD n = 546; All of Us n = 354,400; ns, not significant; PCOS, polycystic ovary syndrome. † *p* values were calculated using Chi-square tests, with Bonferroni correction applied to adjust for multiple comparisons. - indicates the response was excluded from analysis.

**Table 21:** Reproductive symptoms, menstruation, and pregnancy in hEDS and HSD.

Reproductive Symptoms, Menstruation, and Pregnancy	hEDS n (%)	HSD n (%)	hEDS vs. HSD RR (95% CI), *p*-Value †
Pelvic pain	2701 (80.4)	361 (66.1)	1.22 (1.14–1.29), <0.0001
Irregular periods	2363 (70.3)	310 (56.8)	1.24 (1.15–1.34), <0.0001
Pain during sex	2130 (63.4)	274 (50.2)	1.26 (1.16–1.38), <0.0001
Bleeding during sex	1285 (38.2)	139 (25.5)	1.50 (1.29–1.75), <0.0001
Genital overstimulation	594 (17.7)	65 (11.9)	1.49 (1.17–1.89), ns
Tight foreskin	17 (0.5)	4 (0.7)	-
None of the above	247 (7.4)	81 (14.8)	0.50 (0.39–0.63), <0.0001
Age of first menstrual period *^‡^	12.3 (±1.7)	12.4 (±1.5)	(−0.11–0.18), ns
Have been pregnant *	1463 (44.5)	186 (35.1)	-
Average number of pregnancies **	2.5 (±1.4)	2.3 (±1.2)	-
Had pregnancy complications **	983 (67.2)	102 (54.8)	1.23 (1.07–1.40), <0.0001
Pregnancy complication types **			
Preterm labor	339 (23.2)	24 (12.9)	1.80 (1.22–2.64), 0.0111
Spontaneous abortion	583 (39.8)	57 (30.6)	1.30 (1.04–1.63), 0.0247
Prelabor membrane rupture	178 (23.2)	20 (10.8)	1.13 (0.73–1.75), ns
Failure to progress in labor	320 (21.9)	38 (20.4)	1.07 (0.79–1.44), <0.0001
Stillbirth	40 (2.7)	4 (2.2)	-
None of the above	480 (32.8)	84 (45.2)	0.73 (0.61–0.87), <0.0001
Average number of spontaneous abortions ***	1.5 (±0.7)	1.4 (±0.7)	(−0.28–0.10), ns

hEDS n = 3360; HSD n = 546; ns, not significant. † *p* values were calculated using Chi-square tests, with Bonferroni correction applied to adjust for multiple comparisons. ‡ Mann–Whitney U test on data cleaned using ROUT (Q = 1%). * Of those assigned female at birth (hEDS n = 3282; HSD n = 530). ** Of those who reported pregnancy (hEDS n = 1463; HSD n = 186). *** Of those who reported spontaneous abortion (hEDS n = 583; HSD n = 57).

**Table 22:** Prevalence of urinary disorders in hEDS, HSD, and All of Us.

Urinary Disorders	hEDS n (%)	HSD n (%)	hEDS vs. HSD RR (95% CI), *p*-Value †	All of Us n (%)	hEDS vs. All of Us RR (95% CI), *p*-Value †
Recurrent urinary tract infections (UTIs)	980 (29.2)	98 (17.9)	1.63 (1.35–1.96), <0.0001	740 (0.2)	139.68 (127.76–152.72), <0.0001
Urinary incontinence	711 (21.2)	61 (11.2)	1.89 (1.48–2.42), <0.0001	38,020 (10.7)	1.97 (1.85–2.11), <0.0001
Voiding dysfunction	500 (14.9)	43 (7.9)	1.89 (1.40–2.55), 0.0065	60 (0)	878.97 (673.93–1146.39), <0.0001
Kidney stone(s)	450 (13.4)	39 (7.1)	1.88 (1.37–2.57), 0.0240	25,060 (7.1)	1.89 (1.74–2.07), <0.0001
Overactive bladder syndrome	441 (13.1)	39 (7.1)	1.84 (1.34–2.52), 0.0439	8580 (2.4)	5.42 (4.96–5.93), <0.0001
Bladder pain syndrome	215 (6.4)	20 (3.7)	1.75 (1.11–2.74), ns	1520 (0.4)	14.92 (12.99–17.14), <0.0001
Urinary hesitancy	196 (5.8)	18 (3.3)	1.77 (1.10–2.84), ns	3500 (1)	5.91 (5.14–6.79), <0.0001
Kidney disease	85 (2.5)	9 (1.6)	1.53 (0.78–3.03), ns	87,560 (24.7)	0.10 (0.08–0.13), <0.0001
Vesicoureteral reflux	27 (0.8)	1 (0.2)	-	-	-
None of the above	1597 (47.5)	353 (64.7)	0.74 (0.68–0.79), <0.0001	-	-

hEDS n = 3360; HSD n = 546; All of Us n = 354,400; ns, not significant; UTIs, urinary tract infections. † *p* values were calculated using Chi-square tests, with Bonferroni correction applied to adjust for multiple comparisons. - indicates the response was excluded from analysis.

**Table 23:** Prevalence of dermatological disorders in hEDS, HSD, and All of Us.

Dermatological Disorders	hEDS n (%)	HSD n (%)	hEDS vs. HSD RR (95% CI), *p*-Value †	All of Us n (%)	hEDS vs. All of Us RR (95% CI), *p*-Value †
Atopic dermatitis (eczema)	1052 (31.3)	158 (28.9)	1.08 (0.94–1.25), ns	9540 (2.7)	11.63 (11.02–12.27), <0.0001
Hyperhidrosis	223 (6.6)	21 (3.8)	1.73 (1.11–2.67), ns	12,280 (3.5)	1.92 (1.69–2.18), <0.0001
Hidradenitis suppurativa	93 (2.8)	10 (1.8)	1.51 (0.79–2.88), ns	2640 (0.7)	3.72 (3.03–4.56), <0.0001
Hypohidrosis	36 (1.1)	3 (0.5)	-	-	-
Acrogeria	1 (0)	0 (0)	-	-	-
None of the above	1993 (59.3)	352 (64.5)	0.92 (0.86–0.99), ns	-	-

hEDS n = 3360; HSD n = 546; All of Us n = 354,400; ns, not significant. † *p* values were calculated using Chi-square tests, with Bonferroni correction applied to adjust for multiple comparisons. - indicates the response was excluded from analysis.

**Table 24:** Prevalence of dermatological symptoms in hEDS and HSD.

Dermatological Symptoms	hEDS n (%)	HSD n (%)	hEDS vs. HSD RR (95% CI), *p*-Value †
Soft and velvety skin	3059 (91)	332 (60.8)	1.50 (1.40–1.60), <0.0001
Abnormally stretchy skin	2602 (77.4)	178 (32.6)	2.38 (2.10–2.68), <0.0001
Unexplained stretch marks	2473 (73.6)	205 (37.5)	1.96 (1.76–2.19), <0.0001
Poor wound healing	2445 (72.8)	266 (48.7)	1.49 (1.37–1.63), <0.0001
Atrophic scarring	2114 (62.9)	149 (27.3)	2.31 (2.01–2.65), <0.0001
Keratosis pilaris	1820 (54.2)	250 (45.8)	1.18 (1.07–1.30), ns
Recurrent Hives (urticaria)	1636 (48.7)	182 (33.3)	1.46 (1.29–1.65), <0.0001
Hypertrophic scarring	1331 (39.6)	147 (26.9)	1.47 (1.27–1.70), <0.0001
Recurrent folliculitis or abscesses	1212 (36.1)	132 (24.2)	1.49 (1.28–1.74), <0.0001
Acne (if 30+ years old)	979 (29.1)	133 (24.4)	1.20 (1.02–1.40), ns
Keloid scarring	751 (22.4)	67 (12.3)	1.82 (1.44–2.30), <0.0001
None of the above	12 (0.4)	20 (3.7)	0.10 (0.05–0.20), <0.0001

hEDS n = 3360; HSD n = 546; ns, not significant. † *p* values were calculated using Chi-square tests, with Bonferroni correction applied to adjust for multiple comparisons.

**Table 25:** Prevalence of allergy and immune-related diagnoses in hEDS, HSD, and All of Us.

Allergy and Immune-Related Disorders	hEDS n (%)	HSD n (%)	hEDS vs. HSD RR (95% CI), *p*-Value †	All of Us n (%)	hEDS vs. All of Us RR (95% CI), *p*-Value †
Allergies	2707 (80.6)	379 (69.4)	1.16 (1.10–1.23), <0.0001	24,160 (6.8)	11.82 (11.58–12.06), <0.0001
Asthma	1415 (42.1)	163 (29.9)	1.41 (1.23–1.61), <0.0001	65,180 (18.4)	2.29 (2.20–2.38), <0.0001
Mast cell activation syndrome (MCAS)	1003 (29.9)	66 (12.1)	2.47 (1.96–3.11), <0.0001	100 (0)	1057.93 (863.81–1295.66), <0.0001
Chronic sinusitis	899 (26.8)	94 (17.2)	1.55 (1.28–1.88), 0.0011	40,320 (11.4)	2.35 (2.22–2.49), <0.0001
Chronic urticaria	493 (14.7)	26 (4.8)	3.08 (2.10–4.52), <0.0001	220 (0.1)	236.36 (202.38–276.06), <0.0001
Histamine intolerance	365 (10.9)	36 (6.6)	1.65 (1.18–2.29), ns	-	-
Immune deficiency	243 (7.2)	24 (4.4)	1.65 (1.09–2.48), ns	-	-
Nasal polyps	236 (7)	20 (3.7)	1.92 (1.23–3.00), ns	880 (0.2)	28.29 (24.60–32.52), <0.0001
Cold urticaria	170 (5.1)	10 (1.8)	2.76 (1.47–5.19), ns	160 (0)	112.07 (90.55–138.70), <0.0001
Systemic mastocytosis	19 (0.6)	1 (0.2)	-	-	-
None of the above	346 (10.3)	120 (22)	0.47 (0.39–0.56), <0.0001	-	-
Types of Allergies				-	-
Seasonal	1938 (57.7)	248 (45.4)	1.27 (1.15–1.40), <0.0001	-	-
Drug	1529 (45.5)	166 (30.4)	1.50 (1.31–1.71), <0.0001	-	-
Environmental	1435 (42.7)	174 (31.9)	1.34 (1.18–1.52), 0.0010	-	-
Food	1169 (34.8)	142 (26)	1.34 (1.15–1.55), 0.0285	-	-
Metal	510 (15.2)	42 (7.7)	1.97 (1.46–2.67), 0.0018	-	-
Latex	477 (14.2)	40 (7.3)	1.94 (1.42–2.64), 0.0064	-	-
Anaphylaxis Episodes	986 (29.3)	84 (15.4)	1.91 (1.56–2.34), <0.0001	-	-
Psoriasis	221 (6.6)	21 (3.8)	1.71 (1.10–2.65), ns	10,780 (3)	2.16 (1.90–2.46), <0.0001
Lyme disease	154 (4.6)	20 (3.7)	1.25 (0.79–1.98), ns	3120 (0.9)	5.21 (4.44–6.10), <0.0001
Cancer	143 (4.3)	16 (2.9)	1.45 (0.87–2.42), ns	65,980 (18.6)	0.23 (0.19–0.27), <0.0001
Rheumatoid arthritis (RA)	137 (4.1)	19 (3.5)	1.17 (0.73–1.88), ns	11,380 (3.2)	1.27 (1.08–1.50), ns
Sjögren syndrome	127 (3.8)	14 (2.6)	1.47 (0.86–2.54), ns	5600 (1.6)	2.39 (2.01–2.84), <0.0001
Ankylosing spondylitis	73 (2.2)	11 (2)	1.08 (0.58–2.02), ns	1360 (0.4)	5.66 (4.48–7.15), <0.0001
Systemic lupus erythematosus (SLE)	62 (1.8)	6 (1.1)	1.68 (0.73–3.86), ns	5020 (1.4)	1.30 (1.02–1.67), ns
Scleroderma	15 (0.4)	2 (0.4)	-	-	-
Other	168 (5)	25 (4.6)	1.09 (0.72–1.65), ns	-	-
None of the above	2608 (77.6)	452 (82.8)	0.94 (0.90–0.98), ns	-	-

hEDS n = 3360; HSD n = 546; All of Us n = 354,400; ns, not significant; MCAS, mast cell activation syndrome; RA, rheumatoid arthritis; SLE, systemic lupus erythematosus. † *p* values were calculated using Chi-square tests, with Bonferroni correction applied to adjust for multiple comparisons. - indicates the response was excluded from analysis.

**Table 26:** Prevalence of ocular diagnoses in hEDS, HSD, and All of Us.

Ocular Disorders	hEDS n (%)	HSD n (%)	hEDS vs. HSD RR (95% CI), *p*-Value †	All of Us n (%)	hEDS vs. All of Us RR (95% CI), *p*-Value †
Astigmatism	2139 (63.7)	280 (51.3)	1.24 (1.14–1.35), <0.0001	30,440 (8.6)	7.41 (7.21–7.62), <0.0001
Myopia	1904 (56.7)	276 (50.5)	1.12 (1.03–1.22), ns	29,380 (8.3)	6.84 (6.62–7.05), <0.0001
Hyperopia	669 (19.9)	75 (13.7)	1.45 (1.16–1.81), ns	14,840 (4.2)	4.75 (4.44–5.10), <0.0001
Macular degeneration	69 (2.1)	6 (1.1)	1.87 (0.82–4.28), ns	3160 (0.9)	2.30 (1.82–2.92), <0.0001
Keratoconus	42 (1.3)	5 (0.9)	1.37 (0.54–3.43), ns	760 (0.2)	5.83 (4.28–7.94), <0.0001
Retinal detachment	43 (1.3)	6 (1.1)	1.16 (0.50–2.72), ns	4900 (1.4)	0.93 (0.69–1.25), ns
Blue sclerae	554 (16.5)	19 (3.5)	-	-	-
Lens subluxation	21 (0.6)	1 (0.2)	-	-	-
Lens dislocation	5 (0.1)	0 (0)	-	-	-
None of the above	600 (17.9)	149 (27.3)	-	-	-

hEDS n = 3360; HSD n = 546; All of Us n = 354,400; ns, not significant. † *p* values were calculated using Chi-square tests, with Bonferroni correction applied to adjust for multiple comparisons. - indicates the response was excluded from analysis.

**Table 27:** Prevalence of ocular symptoms in hEDS and HSD.

Ocular Symptoms	hEDS n (%)	HSD n (%)	hEDS vs. HSD RR (95% CI), *p*-Value †
Light sensitivity	2971 (88.4)	434 (79.5)	1.11 (1.06–1.16), <0.0001
Visual disturbances	2802 (83.4)	400 (73.3)	1.14 (1.08–1.20), <0.0001
Dry eyes	2307 (68.7)	338 (61.9)	1.11 (1.03–1.19), ns
Double vision	1606 (47.8)	163 (29.9)	1.60 (1.40–1.83), <0.0001
Loss of peripheral vision	709 (21.1)	59 (10.8)	1.95 (1.52–2.51), <0.0001
None of the above	106 (3.2)	37 (6.8)	0.47 (0.32–0.67), 0.0208

hEDS n = 3360; HSD n = 546; ns, not significant. † *p* values were calculated using Chi-square tests, with Bonferroni correction applied to adjust for multiple comparisons.

**Table 28:** Prevalence of dental disorders in hEDS, HSD, and All of Us.

Dental Disorders	hEDS n (%)	HSD n (%)	hEDS vs. HSD RR (95% CI), *p*-Value †	All of Us n (%)	hEDS vs. All of Us RR (95% CI), *p*-Value †
Temporomandibular joint disorder (TMJ disorder)	1660 (49.4)	177 (32.4)	1.52 (1.34–1.73), <0.0001	11,120 (3.1)	15.75 (15.15–16.37), <0.0001
Enamel defects	661 (19.7)	68 (12.5)	1.58 (1.25–1.99), 0.0319	300 (0.1)	232.40 (203.63–265.23), <0.0001
Early onset periodontitis	580 (17.3)	64 (11.7)	1.47 (1.16–1.88), ns	340 (0.1)	179.93 (158.08–204.81), <0.0001
None of the above	1302 (38.8)	310 (56.8)	0.68 (0.63–0.74), <0.0001	-	-

hEDS n = 3360; HSD n = 546; All of Us n = 354,400; ns, not significant; TMJ, temporomandibular joint. † *p* values were calculated using Chi-square tests, with Bonferroni correction applied to adjust for multiple comparisons.

**Table 29:** Dental symptoms in hEDS and HSD.

Dental Symptoms	hEDS n (%)	HSD n (%)	hEDS vs. HSD RR (95% CI), *p*-Value †
Jaw pain	2753 (81.9)	375 (68.7)	1.19 (1.12–1.27), <0.0001
Dental crowding	2358 (70.2)	232 (42.5)	1.65 (1.49–1.83), <0.0001
Subluxation or dislocation of TMJ	2321 (69.1)	261 (47.8)	1.45 (1.32–1.58), <0.0001
Tooth sensitivity without other cause	2169 (64.6)	272 (49.8)	1.30 (1.19–1.41), <0.0001
High or narrow palate	2000 (59.5)	152 (27.8)	2.14 (1.86–2.45), <0.0001
Frequent cavities	1547 (46)	172 (31.5)	1.46 (1.28–1.66), <0.0001
None of the above	41 (1.2)	37 (6.8)	0.18 (0.12–0.28), <0.0001

hEDS n = 3360; HSD n = 546; TMJ, temporomandibular joint. † *p* values were calculated using Chi-square tests, with Bonferroni correction applied to adjust for multiple comparisons.

**Table 30:** Mental health and sleep disorders in hEDS, HSD, and All of Us.

Mental Health and Sleep Disorders	hEDS n (%)	HSD n (%)	hEDS vs. HSD RR (95% CI), *p*-Value †	All of Us n (%)	hEDS vs. All of Us RR (95% CI), *p*-Value †
Anxiety	2512 (74.8)	395 (72.3)	1.03 (0.98–1.09), ns	112,320 (31.7)	2.36 (2.31–2.41), <0.0001
Depression	2285 (68)	352 (64.5)	1.05 (0.99–1.13), ns	112,440 (31.7)	2.14 (2.09–2.19), <0.0001
Post-traumatic stress disorder (PTSD)	1388 (41.3)	150 (27.5)	1.50 (1.30–1.73), <0.0001	21,540 (6.1)	6.80 (6.51–7.09), <0.0001
Insomnia	1281 (38.1)	148 (27.1)	1.41 (1.22–1.62), 0.0004	56,320 (15.9)	2.40 (2.30–2.51), <0.0001
Panic disorder	602 (17.9)	74 (13.6)	1.32 (1.06–1.65), ns	14,260 (4)	4.45 (4.13–4.80), <0.0001
Eating disorder	592 (17.6)	77 (14.1)	1.25 (1.00–1.56), ns	4820 (1.4)	12.95 (11.98–14.01), <0.0001
Restless leg syndrome	553 (16.5)	56 (10.3)	1.60 (1.24–2.08), ns	10,860 (3.1)	5.37 (4.97–5.81), <0.0001
Obstructive sleep apnea	427 (12.7)	51 (9.3)	1.36 (1.03–1.79), ns	60,340 (17)	0.75 (0.68–0.82), <0.0001
Bipolar disorder	230 (6.8)	13 (2.4)	2.88 (1.66–4.99), 0.0386	20,540 (5.8)	1.18 (1.04–1.34), ns
Substance use disorder	96 (2.9)	15 (2.7)	1.04 (0.61–1.78), ns	57,920 (16.3)	0.17 (0.14–0.21), <0.0001
None of the above	409 (12.2)	95 (17.4)	0.70 (0.57–0.86), ns	-	-

hEDS n = 3360; HSD n = 546; All of Us n = 354,400; PTSD, post-traumatic stress disorder. † *p* values were calculated using Chi-square tests, with Bonferroni correction applied to adjust for multiple comparisons. - indicates the response was excluded from analysis; ns, not significant.

**Table 31:** Neurodiversity-related diagnoses in hEDS, HSD, and All of Us.

Neurodiversity-Related Disorders	hEDS n (%)	HSD n (%)	hEDS vs. HSD RR (95% CI), *p*-Value †	All of Us n (%)	hEDS vs. All of Us RR (95% CI), *p*-Value †
Attention deficit hyperactivity disorder (ADHD/ADD)	1172 (34.9)	156 (28.6)	1.22 (1.06–1.40), ns	13,740 (3.9)	9.00 (8.57–9.45), <0.0001
Autism spectrum disorder (ASD)	466 (13.9)	58 (10.6)	1.31 (1.01–1.69), ns	1060 (0.3)	46.37 (41.81–51.43), <0.0001
Obsessive–compulsive disorder (OCD)	466 (13.9)	45 (8.2)	1.68 (1.26–2.25), ns	2860 (0.8)	17.19 (15.68–18.84), <0.0001
Sensory processing disorder	306 (9.1)	34 (6.2)	-	-	-
Learning disorder	299 (8.9)	37 (6.8)	1.31 (0.94–1.83), ns	880 (0.2)	35.84 (31.57–40.68), <0.0001
Tourette’s syndrome	38 (1.1)	4 (0.7)	-	-	-
None of the above	1710 (50.9)	329 (60.3)	0.84 (0.78–0.91), 0.0247	-	-

hEDS n = 3360; HSD n = 546; All of Us n = 354,400; ADHD, attention deficit hyperactivity disorder; ADD, attention deficit disorder; ASD, autism spectrum disorder; OCD, obsessive–compulsive disorder. † *p* values were calculated using Chi-square tests, with Bonferroni correction applied to adjust for multiple comparisons. - indicates the response was excluded from analysis; ns, not significant.

## Data Availability

The original contributions presented in this study are included in the article/supplementary material. Further inquiries can be directed to the corresponding author(s).

## Supplementary Materials

The following supporting information can be downloaded at: https://www.mdpi.com/article/10.3390/jcm14165636/s1, Figure S1: Survey questionnaire; Figure S2: List of conditions; Table S1: Patient demographics; Table S2: Sexual orientation; Table S3: US state residency of patient cohort; Table S4: Country of residence of patient cohort; Table S5: Diagnostic process; Table S6: Symptom severity; Table S7: Pain medication use; Table S8: Cardiopulmonary disorders by age.
