# Supplementary material for: Defining the Chronic Complexities of hEDS and HSD: A Global Survey of Diagnostic Challenges, Life-Long Comorbidities, and Unmet Needs
Source: J Clin Med. 2025 Aug 9;14(16):5636. doi: 10.3390/jcm14165636 (PMC12386360; doi:10.3390/jcm14165636)
Supplement: Supplementary file 1 [file jcm-14-05636-s001.zip › jcm-3767402-supplementary.pdf]

Demographics

What sex were you assigned at birth?

◯ Male
◯ Female
◯ Intersex

What is the gender with which you identify?

◯ Male
◯ Female
◯ Non-binary
◯ Other

If selected "Other", please specify the gender(s) you identify with:

____

What sexual orientation do you identify as? (check all that apply)

☐ Asexual
☐ Bisexual
☐ Fluid
☐ Gay
☐ Lesbian
☐ Pansexual
☐ Queer
☐ Questioning or unsure
☐ Straight (heterosexual)
☐ Other
☐ Prefer not to answer

If selected "Other", please specify the sexuality you identify with:

____

Are you of Hispanic or Latino/a origin?

◯ Yes
◯ No
◯ Prefer not to answer

What is your race? (check all that apply)

☐ White
☐ American Indian/Alaska Native
☐ Asian
☐ Native Hawaiian or other Pacific Islander
☐ Black or African American
☐ Unknown/Other/Unreported
☐ Prefer not to answer

What year were you born? (provide four digit year, for example 1995)

____

What country do you currently reside in?

- ◯ Afghanistan
- ◯ Albania
- ◯ Algeria
- ◯ Andorra
- ◯ Angola
- ◯ Antigua and Barbuda
- ◯ Argentina
- ◯ Armenia
- ◯ Australia
- ◯ Austria
- ◯ Azerbaijan
- ◯ The Bahamas
- ◯ Bahrain
- ◯ Bangladesh
- ◯ Barbados
- ◯ Belarus
- ◯ Belgium
- ◯ Belize
- ◯ Benin
- ◯ Bhutan
- ◯ Bolivia
- ◯ Bosnia and Herzegovina
- ◯ Botswana
- ◯ Brazil
- ◯ Brunei
- ◯ Bulgaria
- ◯ Burkina Faso
- ◯ Burundi
- ◯ Cabo Verde
- ◯ Cambodia
- ◯ Cameroon
- ◯ Canada
- ◯ Central African Republic
- ◯ Chad
- ◯ Chile
- ◯ China
- ◯ Colombia
- ◯ Comoros
- ◯ Congo, Democratic Republic of the
- ◯ Congo, Republic of the
- ◯ Costa Rica
- ◯ Côte d'Ivoire
- ◯ Croatia
- ◯ Cuba
- ◯ Cyprus
- ◯ Czech Republic
- ◯ Denmark
- ◯ Djibouti
- ◯ Dominica
- ◯ Dominican Republic
- ◯ East Timor (Timor-Leste)
- ◯ Ecuador
- ◯ Egypt
- ◯ El Salvador
- ◯ Equatorial Guinea
- ◯ Eritrea
- ◯ Estonia
- ◯ Eswatini
- ◯ Ethiopia
- ◯ Fiji
- ◯ Finland
- ◯ France
- ◯ Gabon
- ◯ The Gambia
- ◯ Georgia
- ◯ Germany
- ◯ Ghana

◯ Greece
◯ Grenada
◯ Guatemala
◯ Guinea
◯ Guinea-Bissau
◯ Guyana
◯ Haiti
◯ Honduras
◯ Hungary
◯ Iceland
◯ India
◯ Indonesia
◯ Iran
◯ Iraq
◯ Ireland
◯ Israel
◯ Italy
◯ Jamaica
◯ Japan
◯ Jordan
◯ Kazakhstan
◯ Kenya
◯ Kiribati
◯ Korea, North
◯ Korea, South
◯ Kosovo
◯ Kuwait
◯ Kyrgyzstan
◯ Laos
◯ Latvia
◯ Lebanon
◯ Lesotho
◯ Liberia
◯ Libya
◯ Liechtenstein
◯ Lithuania
◯ Luxembourg
◯ Madagascar
◯ Malawi
◯ Malaysia
◯ Maldives
◯ Mali
◯ Malta
◯ Marshall Islands
◯ Mauritania
◯ Mauritius
◯ Mexico
◯ Micronesia, Federated States of
◯ Moldova
◯ Monaco
◯ Mongolia
◯ Montenegro
◯ Morocco
◯ Mozambique
◯ Myanmar (Burma)
◯ Namibia
◯ Nauru
◯ Nepal
◯ Netherlands
◯ New Zealand
◯ Nicaragua
◯ Niger
◯ Nigeria
◯ North Macedonia
◯ Norway
◯ Oman
◯ Pakistan
◯ Palau
◯ Panama
◯ Papua New Guinea
◯ Paraguay

◯ Peru
◯ Philippines
◯ Poland
◯ Portugal
◯ Qatar
◯ Romania
◯ Russia
◯ Rwanda
◯ Saint Kitts and Nevis
◯ Saint Lucia
◯ Saint Vincent and the Grenadines
◯ Samoa
◯ San Marino
◯ Sao Tome and Principe
◯ Saudi Arabia
◯ Senegal
◯ Serbia
◯ Seychelles
◯ Sierra Leone
◯ Singapore
◯ Slovakia
◯ Slovenia
◯ Solomon Islands
◯ Somalia
◯ South Africa
◯ Spain
◯ Sri Lanka
◯ Sudan
◯ Sudan, South
◯ Suriname
◯ Sweden
◯ Switzerland
◯ Syria
◯ Taiwan
◯ Tajikistan
◯ Tanzania
◯ Thailand
◯ Togo
◯ Tonga
◯ Trinidad and Tobago
◯ Tunisia
◯ Turkey
◯ Turkmenistan
◯ Tuvalu
◯ Uganda
◯ Ukraine
◯ United Arab Emirates
◯ United Kingdom
◯ United States
◯ Uruguay
◯ Uzbekistan
◯ Vanuatu
◯ Vatican City
◯ Venezuela
◯ Vietnam
◯ Yemen
◯ Zambia
◯ Zimbabwe

Did you receive the majority of your medical care in the country you currently reside in?

◯ Yes
◯ No
◯ Unsure

If not, in which country did you receive the majority of your medical care?

- ◯ Afghanistan
- ◯ Albania
- ◯ Algeria
- ◯ Andorra
- ◯ Angola
- ◯ Antigua and Barbuda
- ◯ Argentina
- ◯ Armenia
- ◯ Australia
- ◯ Austria
- ◯ Azerbaijan
- ◯ The Bahamas
- ◯ Bahrain
- ◯ Bangladesh
- ◯ Barbados
- ◯ Belarus
- ◯ Belgium
- ◯ Belize
- ◯ Benin
- ◯ Bhutan
- ◯ Bolivia
- ◯ Bosnia and Herzegovina
- ◯ Botswana
- ◯ Brazil
- ◯ Brunei
- ◯ Bulgaria
- ◯ Burkina Faso
- ◯ Burundi
- ◯ Cabo Verde
- ◯ Cambodia
- ◯ Cameroon
- ◯ Canada
- ◯ Central African Republic
- ◯ Chad
- ◯ Chile
- ◯ China
- ◯ Colombia
- ◯ Comoros
- ◯ Congo, Democratic Republic of the
- ◯ Congo, Republic of the
- ◯ Costa Rica
- ◯ Côte d'Ivoire
- ◯ Croatia
- ◯ Cuba
- ◯ Cyprus
- ◯ Czech Republic
- ◯ Denmark
- ◯ Djibouti
- ◯ Dominica
- ◯ Dominican Republic
- ◯ East Timor (Timor-Leste)
- ◯ Ecuador
- ◯ Egypt
- ◯ El Salvador
- ◯ Equatorial Guinea
- ◯ Eritrea
- ◯ Estonia
- ◯ Eswatini
- ◯ Ethiopia
- ◯ Fiji
- ◯ Finland
- ◯ France
- ◯ Gabon
- ◯ The Gambia
- ◯ Georgia
- ◯ Germany
- ◯ Ghana

◯ Greece
◯ Grenada
◯ Guatemala
◯ Guinea
◯ Guinea-Bissau
◯ Guyana
◯ Haiti
◯ Honduras
◯ Hungary
◯ Iceland
◯ India
◯ Indonesia
◯ Iran
◯ Iraq
◯ Ireland
◯ Israel
◯ Italy
◯ Jamaica
◯ Japan
◯ Jordan
◯ Kazakhstan
◯ Kenya
◯ Kiribati
◯ Korea, North
◯ Korea, South
◯ Kosovo
◯ Kuwait
◯ Kyrgyzstan
◯ Laos
◯ Latvia
◯ Lebanon
◯ Lesotho
◯ Liberia
◯ Libya
◯ Liechtenstein
◯ Lithuania
◯ Luxembourg
◯ Madagascar
◯ Malawi
◯ Malaysia
◯ Maldives
◯ Mali
◯ Malta
◯ Marshall Islands
◯ Mauritania
◯ Mauritius
◯ Mexico
◯ Micronesia, Federated States of
◯ Moldova
◯ Monaco
◯ Mongolia
◯ Montenegro
◯ Morocco
◯ Mozambique
◯ Myanmar (Burma)
◯ Namibia
◯ Nauru
◯ Nepal
◯ Netherlands
◯ New Zealand
◯ Nicaragua
◯ Niger
◯ Nigeria
◯ North Macedonia
◯ Norway
◯ Oman
◯ Pakistan
◯ Palau
◯ Panama
◯ Papua New Guinea
◯ Paraguay

◯ Peru
◯ Philippines
◯ Poland
◯ Portugal
◯ Qatar
◯ Romania
◯ Russia
◯ Rwanda
◯ Saint Kitts and Nevis
◯ Saint Lucia
◯ Saint Vincent and the Grenadines
◯ Samoa
◯ San Marino
◯ Sao Tome and Principe
◯ Saudi Arabia
◯ Senegal
◯ Serbia
◯ Seychelles
◯ Sierra Leone
◯ Singapore
◯ Slovakia
◯ Slovenia
◯ Solomon Islands
◯ Somalia
◯ South Africa
◯ Spain
◯ Sri Lanka
◯ Sudan
◯ Sudan, South
◯ Suriname
◯ Sweden
◯ Switzerland
◯ Syria
◯ Taiwan
◯ Tajikistan
◯ Tanzania
◯ Thailand
◯ Togo
◯ Tonga
◯ Trinidad and Tobago
◯ Tunisia
◯ Turkey
◯ Turkmenistan
◯ Tuvalu
◯ Uganda
◯ Ukraine
◯ United Arab Emirates
◯ United Kingdom
◯ United States
◯ Uruguay
◯ Uzbekistan
◯ Vanuatu
◯ Vatican City
◯ Venezuela
◯ Vietnam
◯ Yemen
◯ Zambia
◯ Zimbabwe

What U.S. state do you reside in currently?

◯ Alabama
◯ Alaska
◯ Arizona
◯ Arkansas
◯ California
◯ Colorado
◯ Connecticut
◯ Delaware
◯ Florida
◯ Georgia
◯ Hawaii
◯ Idaho
◯ Illinois
◯ Indiana
◯ Iowa
◯ Kansas
◯ Kentucky
◯ Louisiana
◯ Maine
◯ Maryland
◯ Massachusetts
◯ Michigan
◯ Minnesota
◯ Mississippi
◯ Missouri
◯ Montana
◯ Nebraska
◯ Nevada
◯ New Hampshire
◯ New Jersey
◯ New Mexico
◯ New York
◯ North Carolina
◯ North Dakota
◯ Ohio
◯ Oklahoma
◯ Oregon
◯ Pennsylvania
◯ Rhode Island
◯ South Carolina
◯ South Dakota
◯ Tennessee
◯ Texas
◯ Utah
◯ Vermont
◯ Virginia
◯ Washington
◯ West Virginia
◯ Wisconsin
◯ Wyoming
◯ District of Columbia

Diagnostic History

Do you have an official diagnosis of Ehlers-Danlos syndrome (EDS) or hypermobility spectrum disorder (HSD)? (including all subtypes of EDS)

◯ Yes
◯ No
◯ Suspected

Which type of EDS/HSD have you been diagnosed with?

◯ Hypermobile EDS (hEDS)
◯ Hypermobility Spectrum Disorder (HSD)
◯ Classical EDS (cEDS)
◯ Classic-like EDS (clEDS)
◯ Cardiac-valvular EDS (cvEDS)
◯ Vascular EDS (vEDS)
◯ Arthrochalasia EDS (aEDS)
◯ Dermatosparaxis EDS (dEDS)
◯ Kyphoscoliotic EDS (kEDS)
◯ Spondylodysplastic EDS (spEDS)
◯ Musculocontractural EDS (mcEDS)
◯ Myopathic EDS (mEDS)
◯ Periodontal EDS (pEDS)
◯ AEBP1-related EDS (clEDS type 2)
◯ Brittle Cornea Syndrome (BCS)

In regards to the above selection of hEDS, were you diagnosed using the 2017 diagnostic criteria?

Click here to see the 2017 hEDS diagnostic criteria

◯ Yes
◯ No
◯ Unsure

How were you diagnosed with hEDS? (i.e. diagnosed prior to 2017)

____

How were you diagnosed with EDS?

◯ Clinical exam only
◯ Clinical genetic testing (i.e. through a doctor or genetic counselor)
◯ Genetic testing panel (i.e. genedx or invitae connective tissue panel)
◯ Other

If selected "Other", please specify:

____

In regards to your EDS diagnosis, what were the results?

◯ Known pathogenic variant
◯ Variant of unknown significance (VUS)
◯ Other

If selected "Other", please specify:

____

In regards to your EDS diagnosis, what were the results?

◯ Known pathogenic variant
◯ Variant of unknown significance (VUS)
◯ Other

If selected "Other", please specify:

____

Which type of medical specialty initially diagnosed you with EDS/HSD?

◯ Cardiology
◯ Dermatology
◯ Emergency medicine
◯ Endocrinology
◯ Family medicine
◯ Gastroenterology
◯ Genetics
◯ Geriatric
◯ Hepatology
◯ Immunology
◯ Internal medicine
◯ Nephrology
◯ Neurology
◯ Ophthalmology
◯ Otolaryngology
◯ Pediatrics
◯ Psychiatry
◯ Radiology
◯ Rheumatology
◯ Sports medicine
◯ Urology
◯ Other

If selected "Other", please specify:

____

Which type of medical provider first suspected or diagnosed you with EDS/HSD?

◯ Medical doctor (MD)
◯ Doctor of Osteopathy (DO)
◯ Physician's assistant (PA)
◯ Nurse practitioner (NP)
◯ Physical therapist (PT)
◯ Chiropractor
◯ Dentist
◯ Other

If selected "Other", please specify:

____

Do you have any other connective tissue diseases that have been diagnosed through genetic testing?

◯ Yes
◯ No

If yes, what connective tissue disorder?

____

What is your Beighton Score?
Beighton Score assessment available here

◯ 0
◯ 1
◯ 2
◯ 3
◯ 4
◯ 5
◯ 6
◯ 7
◯ 8
◯ 9
◯ unknown

At what age was this Beighton score recorded? (please provide a numerical value)

____

Do you have bilateral piezogenic papules (small, raised skin-colored bumps) on the heels of your feet?

For example:

◯ Yes
◯ No
◯ Unsure

Do you have a positive wrist sign (Walker sign) on both sides? To test, grip your wrist with his opposite hand. If the thumb and fifth finger of the hand overlap with each other, the sign is positive.

For example:

◯ Yes
◯ No
◯ Unsure

Do you have a positive thumb sign (Steinberg sign) on both sides? To test, fold your thumb into a closed fist. The sign is positive if the thumb tip extends beyond the palm of your hand.

For example:

◯ Yes
◯ No
◯ Unsure

Do you have an arm span-to-height ratio ≥1.05?

Click here for an arm span-to-height ratio calculator

◯ Yes
◯ No
◯ Unsure

At what age did your EDS/HSD symptoms begin? (please provide a numerical value)

____

What were your first EDS/HSD symptoms related to? (check the best option)

◯ Gastrointestinal
◯ Allergy/Immune
◯ Autonomic dysfunction
◯ Musculoskeletal
◯ Cardiac
◯ Reproductive
◯ Neurological
◯ Dermatological
◯ Hematological
◯ Urinary
◯ Endocrine
◯ Mental health
◯ Oral
◯ Optical
◯ Sleep
◯ Neurodiversity
◯ Other

If selected "Other", please specify:

____

Please elaborate, what were your first EDS/HSD symptoms?

____

At what age were you clinically/genetically diagnosed with EDS/HSD? (please provide a numerical value)

____

Do you feel/believe you were misdiagnosed at any point with another condition/disorder at any point during the diagnostic process? (i.e. fibromyalgia, autoimmune disorder, etc.)

◯ Yes
◯ No
◯ Unsure

What condition do you feel/believe was a misdiagnosis?

____

Do you believe an event initiated and/or heightened your EDS/HSD symptoms?

☐ No or unsure
☐ Physical accident (i.e. car wreck)
☐ Pregnancy
☐ Puberty
☐ Viral or bacterial infection (i.e. Epstein-Barr, COVID-19, etc.)
☐ Psychological/emotional event
☐ Other

If selected "Other", please specify:

____

Do you have a family history of EDS / HSD?

◯ Yes
◯ No
◯ Unknown
◯ Suspected

If yes, which type of EDS / HSD has the relative(s) been diagnosed with? (check all that apply)

☐ Hypermobile EDS (hEDS) by clinical exam
☐ Hypermobility Spectrum Disorder (HSD) by clinical exam
☐ Classical EDS (cEDS)
☐ Classic-like EDS (clEDS)
☐ Cardiac-valvular EDS (cvEDS)
☐ Vascular EDS (vEDS)
☐ Arthrochalasia EDS (aEDS)
☐ Dermatosparaxis EDS (dEDS)
☐ Kyphoscoliotic EDS (kEDS)
☐ Spondylodysplastic EDS (spEDS)
☐ Musculocontractural EDS (mcEDS)
☐ Myopathic EDS (mEDS)
☐ Periodontal EDS (pEDS)
☐ AEBP1-related EDS (clEDS type 2)
☐ Brittle Cornea Syndrome (BCS)

Pain

Do you have chronic pain? (Pain that lasts for longer than 3 months)

◯ Yes
◯ No

Where do you experience chronic pain (pain that lasts 3 months or longer)? (Check all that apply)

☐ Shoulder
☐ Ribs
☐ Lower back
☐ Upper back
☐ Neck
☐ Elbow
☐ Wrist
☐ Fingers
☐ Hip
☐ Knee
☐ Ankle
☐ Toes
☐ Jaw
☐ Head
☐ Abdominal
☐ Widespread
☐ Other (please specify)
☐ None of the above

If selected "Other", please specify:

____

Do you have any of the following symptoms? (check all that apply)

☐ Muscle spasm
☐ Weakness
☐ Numbness
☐ Tingling
☐ None of the above

Have you ever been diagnosed with complex regional pain syndrome (CRPS)?

◯ Yes
◯ No
◯ Suspected

Have you ever tried any of the following pain medications? Including daily use, post-surgical use, etc. (check all that apply)

- [ ] Acetaminophen (Tylenol, Actamin, Aurophen, Mapap, Pharbetol, Tycolene, Tactinal)
- [ ] Acetaminophen/Oxycodone (Xolox, Tylox, Magnacet, Endocet, Primlev, Roxicet, Percocet)
- [ ] Alfentanil (Alfenta)
- [ ] Amitriptyline
- [ ] Aspirin (Bayer, Bufferin, Ecotrin, Excedrin, Vanquish)
- [ ] Aspirin/Oxycodone (Endodan)
- [ ] Atropine/Difenoxin (Motofen)
- [ ] Baclofen (Lioresal)
- [ ] Benzodiazepine (Valium, Xanax, Halcion, Ativan, and Klonopin)
- [ ] Buprenorphine (Buprenex, Butrans)
- [ ] Butorphanol
- [ ] Carisoprodol (Soma, Vanadom)
- [ ] Celebrex
- [ ] Celecoxib
- [ ] Chlorpheniramine/Hydrocodone (TussiCaps)
- [ ] Chlorzoxazone (Lorzone, Parafon Forte DSC, Relax-DS, Remular S)
- [ ] Codeine
- [ ] Cyclobenzaprine (Fexmid, Flexeril)
- [ ] Dantrolene (Dantrium)
- [ ] Diazepam (Diazepam Intensol, Diastat)
- [ ] Diclofenac (Voltaren oral/gel)
- [ ] Etodolac
- [ ] Fentanyl (Sublimaze, Abstral, Subsys, Duragesic, Ionsys)
- [ ] Fluoxetine (Sarafem, Prozac)
- [ ] Gabapentin
- [ ] Hydrocodone (Hysingla, Vantrela, and Zohydro)
- [ ] Hydrocodone/Acetaminophen (Norco, Lortab, Hycet, Zolvit, Zydone, Lorcet, Maxidone, Co-gesic, Liquicet, Xodol, Vicodin, Stagesic, Zamicet)
- [ ] Hydrocodone/Ibuprofen (Vicoprofen, Ibudone, Reprexain)
- [ ] Hydrocodone/Pseudophedrine (Rezira)
- [ ] Hydromorphone (Dilaudid, Palladone, Exalgo)
- [ ] Hydroxyzine
- [ ] Ibuprofen (Advil, Motrin IB)
- [ ] Ibuprofen/Oxycodone (Combunox)
- [ ] Ketamine
- [ ] Ketorolac (Toradol)
- [ ] Low Dose Naltrexone
- [ ] Medical Marijuana
- [ ] Meloxicam
- [ ] Meperidine (Demerol)
- [ ] Metaxalone (Metaxall, Skelaxin)
- [ ] Methadone
- [ ] Methocarbamol (Robaxin)
- [ ] Morphine (Infumorph, Astramorph, Duramorph, DepoDur)
- [ ] Morphine/Naltrexone (Embeda)
- [ ] Naproxen (Aleve)
- [ ] Nortriptyline
- [ ] Orphenadrine (Norflex)
- [ ] Oxycodone (Xtampza ER, Roxicodone, Oxecta)
- [ ] Oxymorphone (Opana)
- [ ] Paroxetine (Paxil)
- [ ] Pentazocine (Talwin)
- [ ] Pregabalin (Lyrica)
- [ ] Remifentanil (Ultiva)
- [ ] Sufentanil (Sufenta)
- [ ] Tapentadol
- [ ] Tepentadol (Nucynta)
- [ ] Tizanidine (Zanaflex)
- [ ] Tramadol (Ryzolt, ConZip, Ultram)
- [ ] Other
- [ ] None of the above

If selected "Other", please specify:

____

Which of the following medications have been helpful for your pain? Please list all that you remember. (check all that apply)

- ☐ Acetaminophen (Tylenol, Actamin, Aurophen, Mapap, Pharbetol, Tycolene, Tactinal)
- ☐ Acetaminophen/Oxycodone (Xolox, Tylox, Magnacet, Endocet, Primlev, Roxicet, Percocet)
- ☐ Alfentanil (Alfenta)
- ☐ Amitriptyline
- ☐ Aspirin (Bayer, Bufferin, Ecotrin, Excedrin, Vanquish)
- ☐ Aspirin/Oxycodone (Endodan)
- ☐ Atropine/Difenoxin (Motofen)
- ☐ Baclofen (Lioresal)
- ☐ Benzodiazepine (Valium, Xanax, Halcion, Ativan, and Klonopin)
- ☐ Buprenorphine (Buprenex, Butrans)
- ☐ Butorphanol
- ☐ Carisoprodol (Soma, Vanadom)
- ☐ Celebrex
- ☐ Celecoxib
- ☐ Chlorpheniramine/Hydrocodone (TussiCaps)
- ☐ Chlorzoxazone (Lorzone, Parafon Forte DSC, Relax-DS, Remular S)
- ☐ Codeine
- ☐ Cyclobenzaprine (Fexmid, Flexeril)
- ☐ Dantrolene (Dantrium)
- ☐ Diazepam (Diazepam Intensol, Diastat)
- ☐ Diclofenac (Voltaren oral/gel)
- ☐ Etodolac
- ☐ Fentanyl (Sublimaze, Abstral, Subsys, Duragesic, Ionsys)
- ☐ Fluoxetine (Sarafem, Prozac)
- ☐ Gabapentin
- ☐ Hydrocodone (Hysingla, Vantrela, and Zohydro)
- ☐ Hydrocodone/Acetaminophen (Norco, Lortab, Hycet, Zolvit, Zydone, Lorcet, Maxidone, Co-gesic, Liquicet, Xodol, Vicodin, Stagesic, Zamicet)
- ☐ Hydrocodone/Ibuprofen (Vicoprofen, Ibudone, Reprexain)
- ☐ Hydrocodone/Pseudophedrine (Rezira)
- ☐ Hydromorphone (Dilaudid, Palladone, Exalgo)
- ☐ Hydroxyzine
- ☐ Ibuprofen (Advil, Motrin IB)
- ☐ Ibuprofen/Oxycodone (Combunox)
- ☐ Ketamine
- ☐ Ketorolac (Toradol)
- ☐ Low Dose Naltrexone
- ☐ Medical Marijuana
- ☐ Meloxicam
- ☐ Meperidine (Demerol)
- ☐ Metaxalone (Metaxall, Skelaxin)
- ☐ Methadone
- ☐ Methocarbamol (Robaxin)
- ☐ Morphine (Infumorph, Astramorph, Duramorph, DepoDur)
- ☐ Morphine/Naltrexone (Embeda)
- ☐ Naproxen (Aleve)
- ☐ Nortriptyline
- ☐ Orphenadrine (Norflex)
- ☐ Oxycodone (Xtampza ER, Roxicodone, Oxecta)
- ☐ Oxymorphone (Opana)
- ☐ Paroxetine (Paxil)
- ☐ Pentazocine (Talwin)
- ☐ Pregabalin (Lyrica)
- ☐ Remifentanil (Ultiva)
- ☐ Sufentanil (Sufenta)
- ☐ Tapentadol
- ☐ Tepentadol (Nucynta)
- ☐ Tizanidine (Zanaflex)
- ☐ Tramadol (Ryzolt, ConZip, Ultram)
- ☐ Other
- ☐ None of the above

If selected "Other", please specify:

____

Autonomic Dysfunction

Have you ever had a formal diagnosis of any of the following conditions related to dysautonomia? (please only check those that you have been diagnosed with by a medical provider)

- ☐ Postural orthostatic tachycardia syndrome (POTS)
- ☐ Orthostatic hypotension
- ☐ Orthostatic intolerance
- ☐ Hyperadrenergic POTS
- ☐ Hypovolemic POTS
- ☐ Autonomic neuropathy
- ☐ Pure autonomic failure
- ☐ General dysautonomia
- ☐ None of the above

Do you suspect that you may have any of the following conditions but are awaiting evaluation/formal diagnosis? (check all that apply)

- ☐ Postural orthostatic tachycardia syndrome (POTS)
- ☐ Orthostatic hypotension
- ☐ Orthostatic intolerance
- ☐ Hyperadrenergic POTS
- ☐ Hypovolemic POTS
- ☐ Autonomic neuropathy
- ☐ Pure autonomic failure
- ☐ General dysautonomia
- ☐ None of the above

Have you ever experienced any of the following? (check all that apply)

- ☐ Fainting (passing out)
- ☐ Dizziness
- ☐ Vertigo (feeling off balance and that the environment around you is spinning)
- ☐ Heart palpitations (feelings of having a fast-beating, fluttering, or pounding heart)
- ☐ Thermoregulatory dysfunction (excessive sweating or hot/cold intolerance)
- ☐ Fatigue (feeling overly tired and low energy)
- ☐ Brain fog (confusion, lack of mental clarity, or forgetfulness)
- ☐ Trouble swallowing
- ☐ None of the above

Neurological

Have you ever had a formal diagnosis of any of the following conditions? (please only check those that you have been diagnosed with by a medical provider)

- [ ] Migraine
- [ ] Trigeminal neuralgia
- [ ] Occipital neuralgia
- [ ] Thoracic outlet syndrome
- [ ] Other nerve entrapments (i.e. sciatica)
- [ ] Small fiber neuropathy (SFN)
- [ ] Tethered cord syndrome or occult tethered cord syndrome
- [ ] Chiari malformation
- [ ] Upper cervical instability (CCI/AAI)
- [ ] Spondylolisthesis/spinal instability
- [ ] Scoliosis
- [ ] Compartment syndrome
- [ ] Dystonia
- [ ] Intracranial hypotension
- [ ] Cerebrospinal fluid leak (CSF leak)
- [ ] Raynaud's phenomenon
- [ ] Myasthenia gravis
- [ ] Multiple sclerosis
- [ ] Tinnitus
- [ ] Herniated disk(s)
- [ ] Syringomyelia (syrinx)
- [ ] Spinal stenosis
- [ ] Kyphoscoliosis
- [ ] Transverse sinus stenosis
- [ ] None of the above

Do you suspect that you may have any of the following conditions but are awaiting evaluation/formal diagnosis?

- [ ] Migraine
- [ ] Trigeminal neuralgia
- [ ] Occipital neuralgia
- [ ] Thoracic outlet syndrome
- [ ] Other nerve entrapments (i.e. sciatica)
- [ ] Small fiber neuropathy (SFN)
- [ ] Tethered cord syndrome or occult tethered cord syndrome
- [ ] Chiari malformation
- [ ] Upper cervical instability (CCI/AAI)
- [ ] Spondylolisthesis/spinal instability
- [ ] Scoliosis
- [ ] Compartment syndrome
- [ ] Dystonia
- [ ] Intracranial hypotension
- [ ] Cerebrospinal fluid leak (CSF leak)
- [ ] Raynaud's phenomenon
- [ ] Myasthenia gravis
- [ ] Multiple sclerosis
- [ ] Tinnitus
- [ ] Herniated disk(s)
- [ ] Syringomyelia (syrinx)
- [ ] Spinal stenosis
- [ ] Kyphoscoliosis
- [ ] Transverse sinus stenosis
- [ ] None of the above

Do you have any neurological problems not listed above?

◯ Yes
◯ No

If yes, please elaborate:

____

Gastrointestinal

Aside from infection or foodborne illness, have you ever experienced any of the following? (check all that apply)

- [ ] Abdominal pain
- [ ] Bloating
- [ ] Nausea
- [ ] Constipation
- [ ] Diarrhea
- [ ] Vomiting
- [ ] Reflux
- [ ] Rectal bleeding
- [ ] Heartburn
- [ ] None of the above

Have you ever had a formal diagnosis of any of the following conditions? (please only check those that you have been diagnosed with by a medical provider)

- [ ] Gastroesophageal reflux disease (GERD)
- [ ] Gastritis
- [ ] Gastric ulcer
- [ ] Duodenal ulcer
- [ ] Gastroparesis/gastric dysmotility
- [ ] Functional dyspepsia
- [ ] Esophageal dysmotility
- [ ] Eosinophilic esophagitis (EoE)
- [ ] Small bowel dysmotility
- [ ] Colon dysmotility
- [ ] Constipation
- [ ] Abdominal hernia
- [ ] Diverticulosis/diverticulitis
- [ ] Celiac disease
- [ ] Non-celiac gluten sensitivity
- [ ] Irritable bowel syndrome-diarrhea
- [ ] Irritable bowel syndrome-constipation
- [ ] Irritable bowel syndrome-mixed
- [ ] Irritable bowel syndrome-undifferentiated
- [ ] Hiatal hernia
- [ ] Cholelithiasis
- [ ] Ulcerative colitis
- [ ] Crohn's disease
- [ ] Superior mesenteric artery syndrome (SMAS)
- [ ] Median arcuate ligament syndrome (MALS)
- [ ] Feeding intolerance
- [ ] Intestinal malrotation
- [ ] Small intestine bacterial overgrowth (SIBO)
- [ ] Gastrointestinal organ rupture
- [ ] Colorectal polyp(s)
- [ ] Visceroptosis
- [ ] None of the above

Do you suspect that you may have any of the following conditions but are awaiting evaluation/formal diagnosis?

☐ Gastroesophageal reflux disease (GERD)
☐ Gastritis
☐ Gastric ulcer
☐ Duodenal ulcer
☐ Gastroparesis/gastric dysmotility
☐ Functional dyspepsia
☐ Esophageal dysmotility
☐ Eosinophilic esophagitis (EoE)
☐ Small bowel dysmotility
☐ Colon dysmotility
☐ Constipation
☐ Abdominal hernia
☐ Diverticulosis/diverticulitis
☐ Celiac disease
☐ Non-celiac gluten sensitivity
☐ Irritable bowel syndrome-diarrhea
☐ Irritable bowel syndrome-constipation
☐ Irritable bowel syndrome-mixed
☐ Irritable bowel syndrome-undifferentiated
☐ Hiatal hernia
☐ Cholelithiasis
☐ Ulcerative colitis
☐ Crohn's disease
☐ Superior mesenteric artery syndrome (SMAS)
☐ Median arcuate ligament syndrome (MALS)
☐ Feeding intolerance
☐ Intestinal malrotation
☐ Small intestine bacterial overgrowth (SIBO)
☐ Gastrointestinal organ rupture
☐ Colorectal polyp(s)
☐ Visceroptosis
☐ None of the above

Have you ever had a feeding tube?

○ Yes
○ No

What kind of feeding tube(s) have you had? (check all that apply)

☐ Peripheral parenteral nutrition (PPN)
☐ Total parenteral nutrition (TPN)
☐ Nasogastric (NG) tube
☐ Nasojejunal (NJ) tube
☐ Nasoduodenal (ND) tube
☐ Orogastric (OG) tube
☐ Oroenteric tube
☐ Gastric (G) tube
☐ Gastrojejunostomy (GJ) tube
☐ Jejunostomy (JEJ, PEJ, or RIJ) tube

Have you ever had any of the following ostomies? (check all that apply)

☐ Colostomy
☐ Ileostomy
☐ None of the above

Do you have any gastrointestinal problems not listed above?

◯ Yes
◯ No

If yes, please elaborate:

____

Cardiovascular

Have you had an echocardiogram to evaluate heart function?

◯ Yes
◯ No

Have you ever had a formal diagnosis of any of the following conditions? (please only check those that you have been diagnosed with by a medical provider)

☐ Aortic valve defect
☐ Pulmonary valve defect
☐ Tricuspid valve defect
☐ Mitral valve defect
☐ Rheumatic heart disease
☐ Ebstein's anomaly
☐ Patent foramen ovale (PFO)
☐ Atrial septal defect (ASD) (not PFO)
☐ Supraventricular tachycardia (SVT)
☐ Spontaneous Coronary Artery Dissection (SCAD)
☐ Heart failure
☐ Coronary artery disease
☐ Myocardial infarction
☐ Atrial fibrillation
☐ Hypertrophic cardiomyopathy
☐ May-Thurner syndrome
☐ Nutcracker syndrome
☐ Stroke (including a transient ischemic attack (TIA))
☐ Other arrhythmia
☐ Aortic aneurysm
☐ None of the above

If you selected stroke, please elaborate:

____

Do you suspect that you may have any of the following conditions but are awaiting evaluation/formal diagnosis?

☐ Aortic valve defect
☐ Pulmonary valve defect
☐ Tricuspid valve defect
☐ Mitral valve defect
☐ Rheumatic heart disease
☐ Ebstein's anomaly
☐ Patent foramen ovale (PFO)
☐ Atrial septal defect (ASD) (not PFO)
☐ Supraventricular tachycardia (SVT)
☐ Spontaneous Coronary Artery Dissection (SCAD)
☐ Heart failure
☐ Coronary artery disease
☐ Myocardial infarction
☐ Atrial fibrillation
☐ Hypertrophic cardiomyopathy
☐ May-Thurner syndrome
☐ Nutcracker syndrome
☐ Stroke (including a transient ischemic attack (TIA))
☐ Other arrhythmia
☐ Aortic aneurysm
☐ None of the above

If you selected stroke, please elaborate:

____

Do you have any other cardiovascular problems not listed above?

◯ Yes
◯ No

If yes, please elaborate:

____

Orthopedic

Have you ever experienced any of the following? (check all that apply)

- ☐ Joint dislocations (separation of two bones where they meet at a joint)
- ☐ Joint subluxations (minor or incomplete dislocation)
- ☐ Unexplained fractures (broken bones with no apparent blunt-force trauma)
- ☐ None of the above

Based on your above selections, which joints have been affected? (check all that apply)

- ☐ Spine
- ☐ Ribs
- ☐ Shoulder
- ☐ Elbow
- ☐ Wrist
- ☐ Fingers
- ☐ Hip
- ☐ Knee
- ☐ Ankle
- ☐ Toes
- ☐ Other (please specify)

If selected "other", please specify:

____

Have you ever had a formal diagnosis of any of the following? (check all that apply)

- ☐ Congenital hip dislocation
- ☐ Congenital muscle hypotonia
- ☐ Slipping rib syndrome
- ☐ Bowing of legs
- ☐ Joint contracture
- ☐ Club foot
- ☐ Osgood-Schlatter disease
- ☐ Pectus excavatum ("funnel chest")
- ☐ Tendon rupture (partial or complete tear of your tendon)
- ☐ Ligament tear (i.e. anterior cruciate ligament/ACL, rotator cuff, etc.)
- ☐ Tendonitis (i.e. "tennis elbow")
- ☐ Bursitis (often occurs in the hip, knee, etc.)
- ☐ None of the above

Do you suspect you might have any of the following? (check all that apply)

- ☐ Congenital hip dislocation
- ☐ Congenital muscle hypotonia
- ☐ Slipping rib syndrome
- ☐ Bowing of legs
- ☐ Joint contracture
- ☐ Club foot
- ☐ Osgood-Schlatter disease
- ☐ Pectus excavatum ("funnel chest")
- ☐ Tendon rupture (partial or complete tear of your tendon)
- ☐ Ligament tear (i.e. anterior cruciate ligament/ACL, rotator cuff, etc.)
- ☐ Tendonitis (i.e. "tennis elbow")
- ☐ Bursitis (often occurs in the hip, knee, etc.)
- ☐ None of the above

Do you have any other orthopedic problems not listed above?

◯ Yes
◯ No

If yes, please elaborate:

____

Hematological

Have you ever had a formal diagnosis of any of the following? (check all that apply)

☐ Von Willebrand disease
☐ Thrombocytosis
☐ Hemophilia
☐ Deep vein thrombosis (DVT)
☐ Pulmonary embolism (PE)
☐ Arterial thrombosis
☐ Antiphospholipid antibody syndrome (APLS)
☐ Pernicious anemia
☐ None of the above

Do you suspect you might have any of the following? (check all that apply)

☐ Von Willebrand disease
☐ Thrombocytosis
☐ Hemophilia
☐ Deep vein thrombosis (DVT)
☐ Pulmonary embolism (PE)
☐ Arterial thrombosis
☐ Antiphospholipid antibody syndrome (APLS)
☐ Pernicious anemia
☐ None of the above

Do you have easy or severe bruising that is not due to blood thinning medication?

◯ Yes
◯ No
◯ Unsure

Do you have any hematological problems not listed above?

◯ Yes
◯ No

If yes, please elaborate:

____

Endocrinology

Have you ever had a formal diagnosis of any of the following conditions? (please only check those that you have been diagnosed with by a medical provider)

- ☐ Addison's disease
- ☐ Adrenal insufficiency (non-autoimmune)
- ☐ Pituitary tumor/adenoma
- ☐ Pineal cyst
- ☐ Hyperthyroidism (non-autoimmune)
- ☐ Hypothyroidism (non-autoimmune)
- ☐ Hashimoto's disease
- ☐ Cushing disease
- ☐ Grave's disease
- ☐ Diabetes, type 1
- ☐ Diabetes, type 2
- ☐ Diabetes, gestational
- ☐ Hyperparathyroidism
- ☐ Congenital adrenal hyperplasia
- ☐ Multiple endocrine neoplasia
- ☐ None of the above

Do you suspect that you may have any of the following conditions but are awaiting evaluation/formal diagnosis?

- ☐ Addison's disease
- ☐ Adrenal insufficiency (non-autoimmune)
- ☐ Pituitary tumor/adenoma
- ☐ Pineal cyst
- ☐ Hyperthyroidism (non-autoimmune)
- ☐ Hypothyroidism (non-autoimmune)
- ☐ Hashimoto's disease
- ☐ Cushing disease
- ☐ Grave's disease
- ☐ Diabetes, type 1
- ☐ Diabetes, type 2
- ☐ Diabetes, gestational
- ☐ Hyperparathyroidism
- ☐ Congenital adrenal hyperplasia
- ☐ Multiple endocrine neoplasia
- ☐ None of the above

Have you ever used any of the following hormone-based therapies? (check all that apply)

- ☐ Birth control - non-hormonal
- ☐ Birth control - pill
- ☐ Birth control - patch
- ☐ Birth control - hormone-based intrauterine device (IUD)
- ☐ Birth control - arm implant
- ☐ Birth control - vaginal ring
- ☐ Birth control - shot
- ☐ Birth control - other (hormone-based)
- ☐ Gender affirming therapy - hormone replacement
- ☐ Gender affirming hormone therapy - puberty blockers
- ☐ Hormone replacement therapy - menopause
- ☐ None of the above

Did you notice a change in symptoms after starting hormone-based therapy?

- ◯ Yes
- ◯ No
- ◯ Unsure

If yes, please elaborate on your experience:

If yes, which hormone based therapy do you suspect caused the change in symptoms? (check all that apply)

☐ Birth control - non-hormonal
☐ Birth control - pill
☐ Birth control - patch
☐ Birth control - hormone-based intrauterine device (IUD)
☐ Birth control - arm implant
☐ Birth control - vaginal ring
☐ Birth control - shot
☐ Birth control - other (hormone-based)
☐ Gender affirming therapy - hormone replacement
☐ Gender affirming hormone therapy - puberty blockers
☐ Hormone replacement therapy - menopause
☐ None of the above

Based on your above selection(s), which hormones were you treated with that caused a change in symptoms?

☐ Estrogen
☐ Progestin
☐ Mixed estrogen and progestin
☐ Testosterone
☐ Unsure
☐ Other

If selected "Other", please specify:

____

Do you have any other endocrine problems not listed above?

◯ Yes
◯ No

If yes, please elaborate:

____

Reproductive Health

At what age was your first menstrual period? (please provide a numerical value or "N/A" if not applicable)

____

Have you ever been pregnant?

◯ Yes
◯ No
◯ Not applicable to me

If yes, how many times? (please provide a numerical value)

____

Have you experienced any of the following? (check all that apply)

☐ Preterm labor (baby is birthed between 20 to 37 weeks of pregnancy)
☐ Spontaneous abortion (loss of pregnancy naturally before 20 weeks of pregnancy)
☐ Stillbirth (death or loss of a baby anytime after 20 weeks of pregnancy or during delivery)
☐ Prelabor membrane rupture (leakage of amniotic fluid before onset of labor)
☐ Failure to progress in labor (baby isn't born after 20 or more hours of contractions)
☐ None of the above

How many spontaneous abortions (miscarriages before 20 weeks) have you had?

____

Have you ever had a formal diagnosis of any of the following conditions? (please only check those that you have been diagnosed with by a medical provider)

☐ Pelvic organ prolapse
☐ Pelvic congestion syndrome
☐ Infertility
☐ Endometriosis
☐ Polycystic ovary syndrome (PCOS)
☐ Vulvodynia (chronic pain of the vulva)
☐ Vaginismus (involuntary vaginal muscle spasms)
☐ Enlarged prostate gland
☐ Premature ejaculation
☐ Peyronie's disease
☐ Cryptorchidism (issue with testicles descending)
☐ Hypogonadism (not producing enough testosterone and/or sperm)
☐ Erectile dysfunction
☐ Testicular torsion
☐ Penile fracture
☐ None of the above

Do you suspect that you may have any of the following conditions but are awaiting evaluation/formal diagnosis? (check all that apply)

☐ Pelvic organ prolapse
☐ Pelvic congestion syndrome
☐ Infertility
☐ Endometriosis
☐ Polycystic ovary syndrome (PCOS)
☐ Vulvodynia (chronic pain of the vulva)
☐ Vaginismus (involuntary vaginal muscle spasms)
☐ Enlarged prostate gland
☐ Premature ejaculation
☐ Peyronie's disease
☐ Cryptorchidism (issue with testicles descending)
☐ Hypogonadism (not producing enough testosterone and/or sperm)
☐ Erectile dysfunction
☐ Testicular torsion
☐ Penile fracture
☐ None of the above

Have you ever experienced any of the following? (check all that apply)

☐ Pelvic pain
☐ Bleeding during sex
☐ Irregular periods
☐ Genital overstimulation
☐ Pain during sex
☐ Tight foreskin
☐ None of the above

Do you have any reproductive health problems not listed above?

◯ Yes
◯ No

If yes, please elaborate:

____

Urinary

Have you ever had a formal diagnosis of any of the following conditions? (please only check those that you have been diagnosed with by a medical provider)

- ☐ Overactive bladder syndrome
- ☐ Voiding dysfunction (difficulty fully emptying bladder)
- ☐ Bladder pain syndrome
- ☐ Recurrent urinary tract infections (UTIs)
- ☐ Urinary incontinence (leaking urine by accident)
- ☐ Urinary hesitancy
- ☐ Vesicoureteral reflux
- ☐ Kidney stone(s)
- ☐ None of the above

Do you suspect that you may have any of the following conditions but are awaiting evaluation/formal diagnosis?

- ☐ Overactive bladder syndrome
- ☐ Voiding dysfunction (difficulty fully emptying bladder)
- ☐ Bladder pain syndrome
- ☐ Recurrent urinary tract infections (UTIs)
- ☐ Urinary incontinence (leaking urine by accident)
- ☐ Urinary hesitancy
- ☐ Vesicoureteral reflux
- ☐ Kidney stone(s)
- ☐ None of the above

Do you have any urinary problems not listed above?

- ◯ Yes
- ◯ No

If yes, please elaborate:

____

Dermatological

Do you have or have you ever experienced any of the following? (check all that apply)

☐ Poor wound healing
☐ Abnormally stretchy skin (i.e. if the skin on the backside of your wrist stretches at least 1.5 cm)
☐ Atrophic scarring (sunken scarring)
☐ Hypertrophic scarring (firm, raised scarring)
☐ Keloid scarring (firm, raised scarring that extends beyond the original injury)
☐ Soft & velvety skin (i.e. others commenting that you have soft skin)
☐ Keratosis pilaris (i.e. tiny bumps on upper arms)
☐ Unexplained stretch marks (stretch marks without cause)
☐ Recurrent folliculitis or abscesses (persistent infected hair follicles)
☐ Recurrent Hives or Urticaria
☐ Acne (if 30+ years old)
☐ None of the above

Have you ever had a formal diagnosis of any of the following conditions? (please only check those that you have been diagnosed with by a medical provider)

☐ Hyperhidrosis
☐ Hypohidrosis
☐ Psoriasis
☐ Acrogeria
☐ Atopic dermatitis (eczema)
☐ Hidradenitis suppurativa
☐ None of the above

Do you suspect that you may have any of the following conditions but are awaiting evaluation/formal diagnosis?

☐ Hyperhidrosis
☐ Hypohidrosis
☐ Psoriasis
☐ Acrogeria
☐ Atopic dermatitis (eczema)
☐ Hidradenitis suppurativa
☐ None of the above

Do you have any skin conditions not listed above?

◯ Yes
◯ No

If yes, what skin condition(s)?

____

Allergy and Immunology

Have you ever had a formal diagnosis of any of the following conditions? (please only check those that you have been diagnosed with by a medical provider)

☐ Mast cell activation syndrome (MCAS)
☐ Systemic mastocytosis
☐ Immune deficiency
☐ Asthma
☐ Histamine intolerance
☐ Chronic urticaria
☐ Food allergies
☐ Drug allergies
☐ Environmental allergies
☐ Seasonal allergies
☐ Metal allergies
☐ latex allergy
☐ cold urticaria
☐ chronic sinusitis
☐ nasal polyps
☐ None of the above

What kind of immune deficiency?

____

Please specify which foods:

____

Please specify which drugs:

____

Please specify which environmental substances:

____

Please specify which metals:

____

Do you suspect that you may have any of the following conditions but are awaiting evaluation/formal diagnosis?

☐ Mast cell activation syndrome (MCAS)
☐ Systemic mastocytosis
☐ Immune deficiency
☐ Asthma
☐ Histamine intolerance
☐ Chronic urticaria
☐ Food allergies
☐ Drug allergies
☐ Environmental allergies
☐ Seasonal allergies
☐ Metal allergies
☐ latex allergy
☐ cold urticaria
☐ chronic sinusitis
☐ nasal polyps
☐ None of the above

What kind of immune deficiency?

____

Please specify which foods:

____

Please specify which drugs:

____

Please specify which environmental substances:

____

Please specify which metals:

____

Have you ever had a formal diagnosis of any of the following autoimmune conditions? (please only check those that you have been diagnosed with by a medical provider)

☐ Scleroderma
☐ Sjögren syndrome
☐ Systemic lupus erythematosus
☐ Psoriasis
☐ Rheumatoid arthritis
☐ Lyme disease
☐ Ankylosing spondylitis
☐ Other
☐ None of the above

If selected "Other", please specify:

____

Do you suspect that you may have an autoimmune disease(s) but are awaiting evaluation/formal diagnosis?

- ☐ Scleroderma
- ☐ Sjögren syndrome
- ☐ Systemic lupus erythematosus
- ☐ Psoriasis
- ☐ Rheumatoid arthritis
- ☐ Lyme disease
- ☐ Ankylosing spondylitis
- ☐ Other
- ☐ None of the above

If selected "Other", please specify:

____

Have you ever had an episode of anaphylaxis?

- ◯ Yes
- ◯ No
- ◯ Unknown

If yes, which allergy/allergies caused the anaphylaxis episode(s)?

____

Do you have any allergy or immunology problems not listed above?

- ◯ Yes
- ◯ No

If yes, please elaborate:

____

Ocular

Have you ever had a formal diagnosis of any of the following conditions? (please only check those that you have been diagnosed with by a medical provider)

☐ Lens subluxation
☐ Lens dislocation
☐ Retinal detachment
☐ Keratoconus
☐ Blue sclerae (i.e. white part of your eyes has a blue tint)
☐ Myopia (nearsightedness)
☐ Hyperopia (farsightedness)
☐ Astigmatism
☐ Macular degeneration
☐ None of the above

Do you suspect that you may have any of the following conditions but are awaiting evaluation/formal diagnosis?

☐ Lens subluxation
☐ Lens dislocation
☐ Retinal detachment
☐ Keratoconus
☐ Blue sclerae (i.e. white part of your eyes has a blue tint)
☐ Myopia (nearsightedness)
☐ Hyperopia (farsightedness)
☐ Astigmatism
☐ Macular degeneration
☐ None of the above

Have you experienced any of the following? (check all that apply)

☐ Light sensitivity
☐ Visual disturbances (floaters, flashes of light, tunnel vision, etc.)
☐ Double vision
☐ Dry eyes
☐ Loss of peripheral vision
☐ None of the above

Do you wear prescription glasses and/or contact lenses?

◯ Yes
◯ No

Do you have any eye problems not listed above?

◯ Yes
◯ No

If yes, please elaborate:

____

Dental

Have you ever experienced any of the following? (check all that apply)

- ☐ Dental crowding
- ☐ High or narrow palate
- ☐ Jaw pain
- ☐ Subluxation or dislocation of temporomandibular joint (jaw)
- ☐ Frequent cavities
- ☐ Tooth sensitivity without other cause
- ☐ None of the above

Have you ever had dental braces?

- ◯ Yes
- ◯ No

Have you ever had a formal diagnosis of any of the following conditions? (please only check those that you have been diagnosed with by a medical provider)

- ☐ Early onset periodontitis (gum disease occurring before the age of 35)
- ☐ Temporomandibular joint disorder (TMJ disorder)
- ☐ Enamel defects
- ☐ None of the above

Do you suspect that you may have any of the following conditions but are awaiting evaluation/formal diagnosis?

- ☐ Early onset periodontitis (gum disease occurring before the age of 35)
- ☐ Temporomandibular joint disorder (TMJ disorder)
- ☐ Enamel defects
- ☐ None of the above

Do you have any dental problems not listed above?

- ◯ Yes
- ◯ No

If yes, please elaborate:

____

Neurodiversity

Have you ever had a formal diagnosis of any of the following conditions? (please only check those that you have been diagnosed with by a medical provider)

☐ Autism spectrum disorder (ASD)
☐ Attention deficit hyperactivity disorder (ADHD/ADD)
☐ Obsessive compulsive disorder (OCD)
☐ Tourette's syndrome
☐ Learning disorder
☐ Sensory processing disorder
☐ None of the above

Do you suspect that you may have any of the following conditions but are awaiting evaluation/formal diagnosis?

☐ Autism spectrum disorder (ASD)
☐ Attention deficit hyperactivity disorder (ADHD/ADD)
☐ Obsessive compulsive disorder (OCD)
☐ Tourette's syndrome
☐ Learning disorder
☐ Sensory processing disorder
☐ None of the above

Do you have an additional neurodivergent disorder that is not listed above?

◯ Yes
◯ No

If yes, please elaborate:

____

Mental Health and Sleep

Have you ever had a formal diagnosis of any of the following conditions? (please only check those that you have been diagnosed with by a medical provider)

☐ Anxiety
☐ Depression
☐ Post-traumatic stress disorder (PTSD)
☐ Bipolar disorder
☐ Eating disorder
☐ Substance use disorder
☐ Panic disorder
☐ Insomnia
☐ Obstructive sleep apnea
☐ Restless leg syndrome
☐ None of the above

Do you suspect that you may have any of the following conditions but are awaiting evaluation/formal diagnosis?

☐ Anxiety
☐ Depression
☐ Post-traumatic stress disorder (PTSD)
☐ Bipolar disorder
☐ Eating disorder
☐ Substance use disorder
☐ Panic disorder
☐ Insomnia
☐ Obstructive sleep apnea
☐ Restless leg syndrome
☐ None of the above

Do you have any mental health or sleep problems not listed above?

◯ Yes
◯ No

If yes, please elaborate:

____

Additional Medical Questions

Have you ever had a formal diagnosis of any of the following conditions? (please only check those that you have been diagnosed with by a medical provider)

☐ Alpha-1 antitrypsin deficiency
☐ Porphyria
☐ Osteoporosis
☐ Cancer
☐ Osteopenia
☐ Kidney disease
☐ Lung disease
☐ Liver disease
☐ Parkinson's disease
☐ Myalgic encephalomyelitis/chronic fatigue syndrome (ME/CFS)
☐ Obesity
☐ Lipedema
☐ Lymphedema
☐ Hearing impairment
☐ Appendicitis
☐ Pneumothorax
☐ Voice disorder
☐ None of the above

What type of cancer?

____

Please specify what type of voice disorder:

____

Do you suspect that you may have any of the following conditions but are awaiting evaluation/formal diagnosis?

☐ Alpha-1 antitrypsin deficiency
☐ Porphyria
☐ Osteoporosis
☐ Cancer
☐ Osteopenia
☐ Kidney disease
☐ Lung disease
☐ Liver disease
☐ Parkinson's disease
☐ Myalgic encephalomyelitis/chronic fatigue syndrome (ME/CFS)
☐ Obesity
☐ Lipedema
☐ Lymphedema
☐ Hearing impairment
☐ Appendicitis
☐ Pneumothorax
☐ Voice disorder
☐ None of the above

What type of cancer?

____

Please specify what type of voice disorder:

____

Do you have any verified anatomical abnormalities? (i.e. cervical rib, rotated heart, retroverted uterus, etc.)

◯ Yes
◯ No

If yes, please specify any anatomical abnormalities:

____

Have you ever received a formal diagnosis of Long COVID?

◯ Yes
◯ No
◯ Suspected

If yes, when were you diagnosed with Long COVID?

◯ I was diagnosed with Long COVID prior to my EDS/HSD diagnosis.
◯ I was diagnosed with EDS/HSD prior to my Long COVID diagnosis.

How has Long COVID impacted your hypermobility, diagnosis, or symptoms? (option to write "N/A" or "no impact")

____

Have you ever had complications with anesthesia?

◯ Yes
◯ No
◯ Unsure

If yes, check all that apply:

☐ General anesthesia (i.e. during surgery)
☐ Local anesthesia (i.e. Lidocaine)
☐ Other

If selected "Other", please specify:

____

Which kind of anesthesia complication have you experienced? (check all that apply)

☐ Insufficient pain control
☐ Shortened effect (i.e. woke up early)
☐ Intubation complication
☐ Anaphylaxis or allergic reaction
☐ Other

How many medical specialists have you seen within the past year? (please provide a numerical value)

____

What medical specialists have you seen within the past year? (check all that apply)

- ☐ Cardiology
- ☐ Dermatology
- ☐ Emergency medicine
- ☐ Endocrinology
- ☐ Gastroenterology
- ☐ Family medicine
- ☐ Geriatric
- ☐ Hepatology
- ☐ Immunology
- ☐ Internal medicine
- ☐ Nephrology
- ☐ Neurology
- ☐ Ophthalmology
- ☐ Otolaryngology
- ☐ Psychiatry
- ☐ Radiology
- ☐ Rheumatology
- ☐ Sports medicine
- ☐ Urology
- ☐ Other
- ☐ None of the above

If selected "Other", please specify:

____

In your experience, which issue has been the most severe?

- ○ Chronic pain
- ○ Gastrointestinal Symptoms
- ○ Allergic Symptoms
- ○ Autonomic dysfunction
- ○ Joint Manifestations
- ○ Gynecological Symptoms
- ○ Neurological Symptoms
- ○ Dermatological Symptoms
- ○ Urinary Symptoms
- ○ Endocrine Dysfunction
- ○ Mental health
- ○ Dental Manifestations
- ○ Vision Dysfunction
- ○ Sleep Issues
- ○ Neurodiversity
- ○ Other

If selected "Other", please specify:

____

In your experience, which issue has been the 2nd most severe?

◯ Chronic pain
◯ Gastrointestinal Symptoms
◯ Allergic Symptoms
◯ Autonomic dysfunction
◯ Joint Manifestations
◯ Gynecological Symptoms
◯ Neurological Symptoms
◯ Dermatological Symptoms
◯ Urinary Symptoms
◯ Endocrine Dysfunction
◯ Mental health
◯ Dental Manifestations
◯ Vision Dysfunction
◯ Sleep Issues
◯ Neurodiversity
◯ Other

If selected "Other", please specify:

____

In your experience, which issue has been the 3rd most severe?

◯ Chronic pain
◯ Gastrointestinal Symptoms
◯ Allergic Symptoms
◯ Autonomic dysfunction
◯ Joint Manifestations
◯ Gynecological Symptoms
◯ Neurological Symptoms
◯ Dermatological Symptoms
◯ Urinary Symptoms
◯ Endocrine Dysfunction
◯ Mental health
◯ Dental Manifestations
◯ Vision Dysfunction
◯ Sleep Issues
◯ Neurodiversity
◯ Other

If selected "Other", please specify:

____

Have you ever been admitted as an "inpatient" to the hospital for an EDS/HSD or a related condition? (including overnight surgery stays)

◯ Yes
◯ No

How many times have you been hospitalized as an "inpatient" for an EDS/HSD or a related condition in the last year?

◯ 0
◯ 1
◯ 2
◯ 3+

Have you ever tried using non-traditional practices (i.e. chiropractic care, massage, meditation, acupuncture, etc.) in the treatment of your EDS/HSD?

◯ Yes
◯ No

If yes, check all that apply:

☐ Acupuncture
☐ Massage
☐ Chiropractic
☐ Meditation
☐ Ayurveda
☐ Homeopathy
☐ Naturopathy
☐ Chinese medicine
☐ Hypnosis
☐ Functional medicine
☐ Other

If selected "Other", please specify:

____

What is the furthest distance you have traveled to receive medical care pertaining to EDS treatment? (approximately)

◯ Less than 300 miles (Less than 499 km)
◯ 300 to 599 miles (500-999 km)
◯ 600 to 999 miles (1000-1599 km)
◯ 1000 to 2000 miles (1600-3200 km)
◯ More than 2000 miles (More than 3200 km)

Who coordinates your medical care between specialists?

☐ Self
☐ Family member
☐ Medical provider
☐ Other

Have you ever had to pay out-of-pocket for medical care from a specialist not covered by insurance?

◯ Yes
◯ No

On average, how many hours per week do you spend coordinating and receiving medical care, including working with insurance, coordinating with medical providers, and attending appointments?

◯ Less than 5 hours
◯ 5 to 10 hours
◯ 11 to 15 hours
◯ 16 to 20 hours
◯ More than 20 hours

Is there anything else you would like us to know about your health-related to EDS/HSD or related conditions?

What is/are your primary source(s) of information on EDS/HSD? (check all that apply)

☐ Social media
☐ Research journal articles
☐ Websites
☐ Podcasts
☐ Books
☐ Word of mouth
☐ Physicians
☐ Prefer not to answer

Supplementary Figure S2

List of Conditions

Abdominal hernia, Acrogeria, Addisons disease, Adrenal insufficiency (non-autoimmune), Allergies (food, drug, environmental, seasonal, metal, and latex), Alpha-1 antitrypsin deficiency, Ankylosing spondylitis, Antiphospholipid syndrome, Anxiety, Aortic aneurysm, Aortic valve defect, Appendicitis, Arterial thrombosis, Asthma, Astigmatism, Atopic dermatitis, Atrial fibrillation, Atrial septal defect (not PFO), Attention-deficit/hyperactivity disorder/ADD, Autism spectrum disorder, autonomic dysfunction (general dysautonomia, pure autonomic failure, orthostatic hypotension, and orthostatic intolerance), Autonomic neuropathy, Bipolar disorder, Bladder pain syndrome, Bursitis, Cancer, Celiac disease, Cerebrospinal fluid leak, Chiari malformation, Cholelithiasis, Chronic sinusitis, Chronic urticaria, Club foot, Cold urticaria, Colorectal polyp(s), Complex regional pain syndrome, Congenital adrenal hyperplasia, Congenital hip dislocation, Congenital muscle hypotonia, Constipation, Coronary artery disease, Cranio-cervical instability/atlantoaxial instability, Crohn's disease, Cryptorchidism, Cushing disease, Deep vein thrombosis, Depression, Diabetes (gestational), Diabetes (type 1), Diabetes (type 2), Diverticulosis/diverticulitis, Dystonia, Early onset periodontitis, Eating disorder, Ebstein anomaly, Endometriosis, Enlarged prostate gland, Eosinophilic esophagitis, Erectile dysfunction, Functional dyspepsia, Gastritis, Gastroesophageal reflux disease, gastrointestinal dysmotility (colon, esophageal, gastric, and small bowel), Gastrointestinal organ rupture, Graves' disease, Hashimoto's disease, Hearing impairment, Heart failure, Hemophilia, Herniated disk(s), Hiatal hernia, Hidradenitis suppurativa, Hyperhidrosis, Hyperopia, Hyperparathyroidism, Hyperthyroidism (non-autoimmune), Hypertrophic cardiomyopathy, Hypogonadism, Hypohidrosis, Hypothyroidism (non-autoimmune), Immune deficiency, Infertility, Insomnia, Intestinal malrotation, Intracranial hypotension, Irritable bowel syndrome (diarrhea, constipation, mixed, and undifferentiated), Joint contracture, Keratoconus, Kidney disease, Kidney stone(s), Kyphoscoliosis, Learning disorder, Lens dislocation, Lens subluxation, Ligament tear, Lipedema, Liver disease, Lung disease, Lyme disease, Lymphedema, Macular degeneration, Mast cell activation syndrome, May-Thurner syndrome, Median arcuate ligament syndrome, Migraine, Mitral valve defect, Multiple endocrine neoplasia, Multiple sclerosis, Myalgic Encephalomyelitis/chronic fatigue syndrome, Myasthenia gravis, Myocardial infarction, Myopia, Nasal polyp(s), Non-celiac gluten sensitivity, Nutcracker syndrome, Obesity, Obsessive-compulsive disorder, Obstructive sleep apnea, Occipital neuralgia, Osgood-Schlatter disease, Osteopenia, Osteoporosis, Other arrhythmia, Other nerve entrapments, Overactive bladder syndrome, Panic disorder, Parkinsons disease, Patent foramen ovale, Pectus excavatum, Pelvic congestion syndrome, Pelvic organ prolapse, Penile fracture, peptic ulcers (duodenal and gastric), Pernicious anemia, Peyronies disease, Pineal cyst, Pituitary tumor/adenoma, Pneumothorax, Polycystic ovary syndrome, Porphyria, Post-traumatic stress disorder, POTS (hyperadrenergic POTS, hypovolemic POTS, POTS), Premature ejaculation, Psoriasis, Pulmonary embolism, Pulmonary valve defect, Raynaud's phenomenon, Recurrent urinary tract infections, Restless leg syndrome, Retinal detachment, Rheumatic heart disease, Rheumatoid arthritis, Scleroderma, Scoliosis, Sensory processing disorder, Sjögren syndrome, Slipping rib syndrome, Small fiber neuropathy, Small intestinal bacterial overgrowth, Spinal stenosis, Spondylolisthesis/spinal instability, Spontaneous coronary artery dissection, Stroke/transient ischemic attack, Substance use disorder, Superior mesenteric artery syndrome, Supraventricular tachycardia, Syringomyelia (syrinx), Systemic lupus erythematosus, Systemic mastocytosis, Temporomandibular Joint disorder, Tendon rupture, Tendonitis, Testicular torsion, Tethered cord syndrome, Thoracic outlet syndrome, Thrombocytosis, Tinnitus, Tourette's syndrome, Transverse sinus stenosis, Tricuspid valve defect, Trigeminal neuralgia, Ulcerative colitis, Urinary hesitancy, Urinary incontinence, Vaginismus, Vesicoureteral reflux, Visceroptosis, Voice disorder, Voiding dysfunction, Von Willebrand disease, Vulvodynia

Supplementary Table S1

Demographics	hEDS n (%)	HSD n (%)
Sex		
Female	3282 (97.7)	530 (97.1)
Male	78 (2.3)	16 (2.9)
Gender Identity		
Female	2890 (86)	475 (87)
Non-binary	320 (9.5)	43 (7.9)
Male	124 (3.7)	21 (3.8)
Other	26 (0.8)	7 (1.3)
Sex and Gender Differences		
Sex assigned female, identify as male	56 (1.7%)	9 (1.6%)
Sex assigned female, identify as non-binary	315 (9.4%)	42 (7.7%)
Sex assigned male, identify as female	5 (0.1%)	3 (0.5%)
Sex assigned male, identify as non-binary	5 (0.1%)	1 (0.2%)
Sexual Orientation*		
Straight (heterosexual)	1861 (55.4)	333 (61)
LGBTQIA+	1491 (44.4)	213 (39)
Prefer not to answer	81 (2.4)	14 (2.6)
LGBTQIA+ identification*		
Bisexual	670 (19.9)	96 (17.6)
Queer	396 (11.8)	68 (12.5)
Pansexual	342 (10.2)	38 (7)
Asexual	267 (7.9)	37 (6.8)
Lesbian	163 (4.9)	21 (3.8)
Questioning or unsure	97 (2.9)	25 (4.6)
Gay	53 (1.6)	7 (1.3)
Fluid	50 (1.5)	3 (0.5)
Other	41 (1.2)	5 (0.9)
Ethnicity		
Hispanic or Latinx	188 (5.6)	31 (5.7)
Not Hispanic or Latinx	3162 (94.1)	511 (93.6)
Prefer not to answer	10 (0.3)	4 (0.7)
Race*		
White	3219 (95.8)	517 (94.7)
American Indian or Alaska Native	93 (2.8)	7 (1.3)
Asian	72 (2.1)	24 (4.4)
Black or African American	42 (1.3)	3 (0.5)
Native Hawaiian or other Pacific Islander	7 (0.2)	0 (0)
Unknown/Other/Unreported	119 (3.5)	13 (2.4)
Prefer not to answer	23 (0.7)	5 (0.9)
Age Range		
18-25	668 (19.9)	95 (17.4)
26-44	1840 (54.8)	317 (58.1)
45-59	686 (20.4)	113 (20.7)
60+	166 (4.9)	21 (3.8)

Supplementary Table S2

Sexual Orientation by Age Range	hEDS n (%)				HSD n (%)			
	18-25	26-44	45-59	60+	18-25	26-44	45-59	60+
Sexual Orientation								
Straight (heterosexual	262 (7.8)	946 (28.2)	511 (15.2)	143 (4.3)	28 (5.1)	199 (36.4)	89 (16.3)	18 (3.3)
LGBTQIA+	402 (12)	891 (26.5)	180 (5.4)	18 (0.5)	67 (12.3)	126 (23.1)	20 (3.7)	1 (0.2)
Prefer not to answer	16 (0.5)	47 (1.4)	11 (0.3)	7 (0.2)	3 (0.5)	5 (0.9)	4 (0.7)	2 (0.4)
LGBTQIA+ identification								
Bisexual	160 (4.8)	425 (12.6)	76 (2.3)	9 (0.3)	29 (5.3)	57 (10.4)	11 (2)	0 (0)
Queer	113 (3.4)	251 (7.5)	31 (0.9)	1 (0)	21 (3.8)	42 (7.7)	5 (0.9)	0 (0)
Pansexual	76 (2.3)	224 (6.7)	41 (1.2)	1 (0)	9 (1.6)	27 (4.9)	2 (0.4)	0 (0)
Asexual	78 (2.3)	148 (4.4)	35 (1)	6 (0.2)	17 (3.1)	18 (3.3)	1 (0.2)	1 (0.2)
Lesbian	67 (2)	77 (2.3)	18 (0.5)	1 (0)	8 (1.5)	11 (2)	2 (0.4)	0 (0)
Questioning or unsure	30 (0.9)	56 (1.7)	11 (0.3)	0 (0)	8 (1.5)	16 (2.9)	1 (0.2)	0 (0)
Gay	16 (0.5)	33 (1)	4 (0.1)	0 (0)	2 (0.4)	3 (0.5)	2 (0.4)	0 (0)
Fluid	9 (0.3)	31 (0.9)	9 (0.3)	1 (0)	0 (0)	2 (0.4)	1 (0.2)	0 (0)
Other	7 (0.2)	29 (0.9)	5 (0.1)	0 (0)	2 (0.4)	3 (0.5)	0 (0)	0 (0)

Supplementary Table S3

U.S. State Residence	hEDS n (%)	HSD n (%)
California	174 (5.2)	23 (4.2)
South Carolina	124 (3.7)	14 (2.6)
Florida	120 (3.6)	15 (2.7)
Texas	119 (3.5)	8 (1.5)
North Carolina	108 (3.2)	14 (2.6)
Virginia	99 (2.9)	17 (3.1)
Illinois	97 (2.9)	13 (2.4)
Washington	95 (2.8)	13 (2.4)
Pennsylvania	95 (2.8)	7 (1.3)
New York	94 (2.8)	21 (3.8)
Ohio	86 (2.6)	7 (1.3)
Massachusetts	81 (2.4)	12 (2.2)
Georgia	78 (2.3)	6 (1.1)
Maryland	69 (2.1)	16 (2.9)
Colorado	66 (2)	11 (2)
Oregon	59 (1.8)	7 (1.3)
Arizona	58 (1.7)	14 (2.6)
Tennessee	56 (1.7)	4 (0.7)
Michigan	55 (1.6)	3 (0.5)
Wisconsin	48 (1.4)	3 (0.5)

Minnesota	47 (1.4)	7 (1.3)
Missouri	47 (1.4)	5 (0.9)
New Jersey	46 (1.4)	3 (0.5)
Indiana	36 (1.1)	5 (0.9)
Iowa	32 (1)	5 (0.9)
Kentucky	28 (0.8)	0 (0)
Alabama	27 (0.8)	5 (0.9)
Utah	26 (0.8)	3 (0.5)
Connecticut	23 (0.7)	3 (0.5)
Louisiana	21 (0.6)	3 (0.5)
Arkansas	21 (0.6)	1 (0.2)
New Hampshire	20 (0.6)	2 (0.4)
Oklahoma	18 (0.5)	0 (0)
Nebraska	17 (0.5)	2 (0.4)
Maine	15 (0.4)	5 (0.9)
Idaho	15 (0.4)	1 (0.2)
Kansas	15 (0.4)	0 (0)
West Virginia	14 (0.4)	1 (0.2)
Nevada	13 (0.4)	2 (0.4)
Rhode Island	13 (0.4)	1 (0.2)
Montana	12 (0.4)	2 (0.4)
Vermont	10 (0.3)	2 (0.4)
Alaska	10 (0.3)	1 (0.2)
New Mexico	9 (0.3)	1 (0.2)
Delaware	7 (0.2)	0 (0)
District of Columbia	6 (0.2)	1 (0.2)
Mississippi	4 (0.1)	1 (0.2)
South Dakota	4 (0.1)	0 (0)
Wyoming	2 (0.1)	1 (0.2)
Hawaii	2 (0.1)	1 (0.2)
North Dakota	2 (0.1)	0 (0)

Supplementary Table S4

**Country of Residence**	**hEDS n (%)**	**HSD n (%)**
United States	2343 (69.7)	292 (53.5)
United Kingdom	400 (11.9)	107 (19.6)
Canada	142 (4.2)	29 (5.3)
Australia	130 (3.9)	43 (7.9)
New Zealand	59 (1.8)	3 (0.5)
Netherlands	44 (1.3)	15 (2.7)
Germany	42 (1.3)	9 (1.6)
Sweden	39 (1.2)	10 (1.8)
Switzerland	15 (0.4)	2 (0.4)
Ireland	14 (0.4)	3 (0.5)
Belgium	13 (0.4)	8 (1.5)
France	13 (0.4)	4 (0.7)

Italy	12 (0.4)	1 (0.2)
Spain	10 (0.3)	4 (0.7)
South Africa	9 (0.3)	4 (0.7)
Brazil	9 (0.3)	2 (0.4)
Mexico	7 (0.2)	2 (0.4)
Austria	7 (0.2)	0 (0)
Poland	5 (0.1)	1 (0.2)
Israel	5 (0.1)	0 (0)
Czech Republic	4 (0.1)	0 (0)
Finland	4 (0.1)	0 (0)
Norway	4 (0.1)	0 (0)
Portugal	4 (0.1)	0 (0)
Japan	3 (0.1)	1 (0.2)
Denmark	3 (0.1)	0 (0)
Iceland	3 (0.1)	0 (0)
Chile	2 (0.1)	0 (0)
Colombia	2 (0.1)	0 (0)
Korea, South	2 (0.1)	0 (0)
Turkey	2 (0.1)	0 (0)
Argentina	1 (0)	2 (0.4)
Uruguay	1 (0)	1 (0.2)
Angola	1 (0)	0 (0)
Egypt	1 (0)	0 (0)
Estonia	1 (0)	0 (0)
Luxembourg	1 (0)	0 (0)
Romania	1 (0)	0 (0)
Slovenia	1 (0)	0 (0)
Thailand	1 (0)	0 (0)
Central African Republic	0 (0)	1 (0.2)
India	0 (0)	1 (0.2)
Iran	0 (0)	1 (0.2)

Supplementary Table S5

Diagnostic Process	hEDS n (%)	HSD n (%)	hEDS vs. HSD RR (95% CI), p-Value†
Average age of first symptoms	8.5 (±5.6)	11.2 (±6.5)	(2.021-3.208), <0.0001
First symptoms related to hEDS/HSD			
Musculoskeletal	2114 (62.9)	402 (73.6)	0.85 (0.81–0.90), 0.0007
Gastrointestinal	447 (13.3)	39 (7.1)	1.86 (1.36–2.55), 0.0294
Autonomic dysfunction	190 (5.7)	26 (4.8)	1.19 (0.80–1.77), ns
Allergy/Immune	163 (4.9)	17 (3.1)	1.56 (0.95–2.55), ns
Neurological	94 (2.8)	11 (2)	1.39 (0.75–2.58), ns
Cardiac	51 (1.5)	7 (1.3)	1.18 (0.54–2.60), ns
Neurodiversity	47 (1.4)	10 (1.8)	0.76 (0.39–1.50), ns
Urinary	45 (1.3)	5 (0.9)	1.46 (0.58–3.67), ns
Sleep	32 (1)	4 (0.7)	-
Oral	25 (0.7)	2 (0.4)	-
Dermatological	18 (0.5)	0 (0)	-
Reproductive	15 (0.4)	3 (0.5)	-
Optical	13 (0.4)	2 (0.4)	-

Hematological	13 (0.4)	0 (0)	-
Mental health	11 (0.3)	6 (1.1)	0.30 (0.11–0.80), ns
Endocrine	5 (0.1)	2 (0.4)	-
Other	77 (2.3)	10 (1.8)	1.25 (0.65–2.40), ns
First specialist to suspect hEDS/HSD			
Medical doctor (MD)	1985 (59.1)	302 (55.3)	1.07 (0.99–1.16), ns
Physical therapist (PT)	454 (13.5)	104 (19)	0.71 (0.58–0.86), ns
Doctor of Osteopathy (DO)	146 (4.3)	23 (4.2)	1.03 (0.67–1.59), ns
Nurse practitioner (NP)	137 (4.1)	14 (2.6)	1.59 (0.92–2.74), ns
Chiropractor	67 (2)	17 (3.1)	0.64 (0.38–1.08), ns
Physician's assistant (PA)	67 (2)	10 (1.8)	1.09 (0.56–2.10), ns
Dentist	14 (0.4)	1 (0.2)	-
Other	490 (14.6)	75 (13.7)	1.06 (0.85–1.33), ns
Beighton score			
0	0 (0)	0 (0)	-
1	0 (0)	6 (1.1)	-
2	0 (0)	12 (2.2)	-
3	0 (0)	18 (3.3)	-
4	51 (1.5)	37 (6.8)	0.22 (0.15–0.34), <0.0001
5	240 (7.1)	58 (10.6)	0.67 (0.51–0.88), ns
6	346 (10.3)	61 (11.2)	0.92 (0.71–1.19), ns
7	956 (28.5)	116 (21.2)	1.34 (1.13–1.59), ns
8	712 (21.2)	57 (10.4)	2.03 (1.57–2.62), <0.0001
9	1055 (31.4)	57 (10.4)	3.01 (2.34–3.86), <0.0001
unknown	0 (0)	124 (22.7)	-
Average age of Beighton score	31.5 (±11.7)	30.9 (±10.8)	(-1.75-0.52), ns
Believe misdiagnosed in diagnostic process	2018 (60.1)	282 (51.6)	-
Specialist to diagnose hEDS/HSD			
Genetics	1232 (36.7)	81 (14.8)	2.47 (2.01–3.04), <0.0001
Rheumatology	891 (26.5)	215 (39.4)	0.67 (0.60–0.76), <0.0001
Family medicine	296 (8.8)	44 (8.1)	1.09 (0.81–1.48), ns
Sports medicine	137 (4.1)	51 (9.3)	0.44 (0.32–0.59), <0.0001
Internal medicine	117 (3.5)	20 (3.7)	0.95 (0.60–1.51), ns
Cardiology	112 (3.3)	13 (2.4)	1.40 (0.79–2.47), ns
Neurology	110 (3.3)	22 (4)	0.81 (0.52–1.27), ns
Pediatrics	27 (0.8)	11 (2)	0.40 (0.20–0.80), ns
Gastroenterology	23 (0.7)	0 (0)	-
Immunology	13 (0.4)	3 (0.5)	-
Dermatology	10 (0.3)	0 (0)	-
Endocrinology	10 (0.3)	0 (0)	-
Psychiatry	7 (0.2)	1 (0.2)	-
Geriatric	7 (0.2)	0 (0)	-
Emergency medicine	4 (0.1)	2 (0.4)	-
Hepatology	1 (0)	0 (0)	-
Radiology	1 (0)	0 (0)	-
Other	362 (10.8)	83 (15.2)	0.71 (0.57–0.88), ns
Average age when diagnosed with hEDS/HSD	31.4 (±11.7)	31.4 (±11.5)	(-1.07-1.02), ns

Supplementary Table S6

Symptom Severity	hEDS<br>n (%)	HSD<br>n (%)	hEDS vs. HSD<br>RR (95% CI), p-Value†
Most Severe Symptom			
Chronic pain	1604 (47.7)	273 (50)	0.95 (0.87–1.05), ns
Autonomic dysfunction	398 (11.8)	50 (9.2)	1.29 (0.98–1.71), ns
Gastrointestinal symptoms	374 (11.1)	41 (7.5)	1.48 (1.09–2.02), ns
Joint manifestations	351 (10.4)	74 (13.6)	0.77 (0.61–0.97), ns
Neurological symptoms	220 (6.5)	35 (6.4)	1.02 (0.72–1.44), ns
Other	126 (3.8)	17 (3.1)	1.20 (0.73–1.98), ns
Allergic symptoms	85 (2.5)	11 (2)	1.26 (0.67–2.34), ns
Mental health	61 (1.8)	17 (3.1)	0.58 (0.34–0.99), ns
Sleep issues	43 (1.3)	9 (1.6)	0.78 (0.38–1.58), ns
Gynecological symptoms	33 (1)	4 (0.7)	1.34 (0.48–3.77), ns
Urinary symptoms	22 (0.7)	3 (0.5)	1.19 (0.36–3.97), ns
Neurodiversity	12 (0.4)	1 (0.2)	1.95 (0.25–14.97), ns
Endocrine dysfunction	9 (0.3)	7 (1.3)	0.21 (0.08–0.56), ns
Dermatological symptoms	8 (0.2)	1 (0.2)	1.30 (0.16–10.37), ns
Vision dysfunction	8 (0.2)	2 (0.4)	0.65 (0.14–3.05), ns
Dental manifestations	6 (0.2)	1 (0.2)	0.98 (0.12–8.08), ns
Second Most Severe Symptom			
Chronic pain	732 (21.8)	114 (20.9)	1.04 (0.88–1.24), ns
Gastrointestinal symptoms	549 (16.3)	73 (13.4)	1.22 (0.97–1.53), ns
Joint manifestations	508 (15.1)	92 (16.8)	0.90 (0.73–1.10), ns
Autonomic dysfunction	491 (14.6)	60 (11)	1.33 (1.03–1.71), ns
Neurological symptoms	269 (8)	41 (7.5)	1.07 (0.78–1.46), ns
Mental health	187 (5.6)	39 (7.1)	0.78 (0.56–1.09), ns
Allergic symptoms	163 (4.9)	23 (4.2)	1.15 (0.75–1.77), ns
Sleep issues	148 (4.4)	34 (6.2)	0.71 (0.49–1.02), ns
Other	79 (2.4)	17 (3.1)	0.76 (0.45–1.27), ns
Gynecological symptoms	66 (2)	13 (2.4)	0.83 (0.46–1.49), ns
Neurodiversity	43 (1.3)	17 (3.1)	0.41 (0.24–0.72), ns
Urinary symptoms	36 (1.1)	8 (1.5)	0.73 (0.34–1.56), ns
Dental manifestations	32 (1)	7 (1.3)	0.74 (0.33–1.67), ns
Endocrine dysfunction	24 (0.7)	1 (0.2)	3.90 (0.53–28.77), ns
Vision dysfunction	22 (0.7)	4 (0.7)	0.89 (0.31–2.58), ns
Third Most Severe Symptom			
Gastrointestinal symptoms	531 (15.8)	83 (15.2)	1.04 (0.84–1.29), ns
Autonomic dysfunction	501 (14.9)	45 (8.2)	1.81 (1.35–2.42), 0.0172
Joint manifestations	413 (12.3)	58 (10.6)	1.16 (0.89–1.50), ns
Chronic pain	387 (11.5)	57 (10.4)	1.10 (0.85–1.44), ns
Mental health	288 (8.6)	73 (13.4)	0.64 (0.50–0.82), ns
Neurological symptoms	261 (7.8)	36 (6.6)	1.18 (0.84–1.65), ns
Sleep issues	252 (7.5)	46 (8.4)	0.89 (0.66–1.20), ns
Allergic symptoms	237 (7.1)	35 (6.4)	1.10 (0.78–1.55), ns
Gynecological symptoms	122 (3.6)	22 (4)	0.90 (0.58–1.41), ns
Neurodiversity	106 (3.2)	34 (6.2)	0.51 (0.35–0.74), ns
Urinary symptoms	69 (2.1)	6 (1.1)	1.87 (0.82–4.28), ns
Dental manifestations	52 (1.5)	12 (2.2)	0.70 (0.38–1.31), ns
Other	40 (1.2)	14 (2.6)	0.46 (0.25–0.85), ns
Vision dysfunction	35 (1)	8 (1.5)	0.71 (0.33–1.52), ns

Dermatological symptoms	35 (1)	7 (1.3)	0.81 (0.36–1.82), ns
Endocrine dysfunction	31 (0.9)	10 (1.8)	0.50 (0.25–1.02), ns

Supplementary Table S7

**Medication for Pain**	**hEDS N=3360 n (%)**	**hEDS N=Tried n (%)**
**Medication Class**	**Tried**	**Considered Effective**
NSAIDs	3232 (96.2)	1843 (54.9)
Acetaminophen	2871 (85.4)	850 (25.3)
Opioids	2612 (77.7)	1679 (50)
Anticonvulsants	1872 (55.7)	607 (18.1)
Muscle relaxant	1596 (47.5)	804 (23.9)
Benzodiazepines or barbiturates	1586 (47.2)	449 (13.4)
TCAs	1377 (41)	275 (8.2)
SSRIs/SNRIs	1149 (34.2)	101 (3)
Cannabis	1148 (34.2)	781 (23.2)
Opioid antagonist	819 (24.4)	391 (11.6)
Antihistamine	594 (17.7)	80 (2.4)
NMDA antagonist	325 (9.7)	187 (5.6)
Topicals	55 (1.6)	33 (1)
Triptans	26 (0.8)	10 (0.3)
Anticholingeric or antimuscarinic	25 (0.7)	2 (0.1)
DMARD or biologics	21 (0.6)	10 (0.3)
Supplements	21 (0.6)	10 (0.3)
CGRP antagonist	20 (0.6)	16 (0.5)
Steroids	19 (0.6)	11 (0.3)
Atypical antidepressant	12 (0.4)	2 (0.1)
Benzoxazocine	11 (0.3)	7 (0.2)
Beta blocker	7 (0.2)	3 (0.1)
Alpha agonist	5 (0.1)	2 (0.1)
Calcium channel blocker	3 (0.1)	2 (0.1)
Antipyschotics	3 (0.1)	0 (0)
Non-benzodiazepines	3 (0.1)	0 (0)
Dopamine agonist	2 (0.1)	1 (0)
None of the above	280 (8.3)	-

Supplementary Table S8

Cardiopulmonary Disorders by Age Range	hEDS n (%)				HSD n (%)			
	**18-25**	**26-44**	**45-59**	**60+**	**18-25**	**26-44**	**45-59**	**60+**
Mitral valve defect	70 (2.1)	285 (8.5)	139 (4.1)	53 (1.6)	2 (0.4)	11 (2)	13 (2.4)	4 (0.7)
Other arrhythmia	77 (2.3)	263 (7.8)	113 (3.4)	32 (1)	5 (0.9)	32 (5.9)	14 (2.6)	2 (0.4)
Supraventricular tachycardia (SVT)	43 (1.3)	146 (4.3)	55 (1.6)	19 (0.6)	2 (0.4)	13 (2.4)	5 (0.9)	0 (0)
Tricuspid valve defect	26 (0.8)	113 (3.4)	38 (1.1)	24 (0.7)	1 (0.2)	9 (1.6)	3 (0.5)	0 (0)
Aortic valve defect	16 (0.5)	61 (1.8)	22 (0.7)	12 (0.4)	0 (0)	1 (0.2)	2 (0.4)	0 (0)
Lung disease	24 (7.1)	37 (1.1)	32 (1.0)	6 (0.2)	8 (1.5)	18 (3.3)	14 (2.6)	2 (0.4)
Atrial fibrillation	14 (0.4)	41 (1.2)	23 (0.7)	21 (0.6)	1 (0.2)	3 (0.5)	2 (0.4)	1 (0.2)
Stroke	5 (0.1)	41 (1.2)	23 (0.7)	11 (0.3)	1 (0.2)	4 (0.7)	0 (0)	0 (0)
May-Thurner syndrome	19 (0.6)	28 (0.8)	13 (0.4)	2 (0.1)	1 (0.2)	4 (0.7)	1 (0.2)	0 (0)
Nutcracker syndrome	22 (0.7)	30 (0.9)	5 (0.1)	4 (0.1)	0 (0)	1 (0.2)	3 (0.5)	0 (0)
Pulmonary valve defect	9 (0.3)	31 (0.9)	10 (0.3)	10 (0.3)	1 (0.2)	0 (0)	2 (0.4)	0 (0)
Patent foramen ovale (PFO)	8 (0.2)	29 (0.9)	9 (0.3)	5 (0.1)	0 (0)	4 (0.7)	0 (0)	0 (0)
Heart failure	2 (0.1)	14 (0.4)	18 (0.5)	10 (0.3)	0 (0)	1 (0.2)	0 (0)	1 (0.2)
Coronary artery disease	0 (0)	4 (0.1)	20 (0.6)	12 (0.4)	0 (0)	1 (0.2)	1 (0.2)	0 (0)
Aortic aneurysm	1 (0)	9 (0.3)	16 (0.5)	6 (0.2)	0 (0)	0 (0)	0 (0)	0 (0)
Atrial septal defect (ASD (not PFO)	6 (0.2)	9 (0.3)	7 (0.2)	2 (0.1)	1 (0.2)	0 (0)	0 (0)	0 (0)
Myocardial infarction	0 (0)	8 (0.2)	6 (0.2)	3 (0.1)	0 (0)	0 (0)	1 (0.2)	1 (0.2)
Hypertrophic cardiomyopathy	3 (0.1)	2 (0.1)	4 (0.1)	6 (0.2)	0 (0)	0 (0)	0 (0)	2 (0.4)
Rheumatic heart disease	2 (0.1)	1 (0)	1 (0)	1 (0)	0 (0)	0 (0)	0 (0)	0 (0)
Spontaneous Coronary Artery Dissection (SCAD)	0 (0)	2 (0.1)	2 (0.1)	0 (0)	0 (0)	0 (0)	0 (0)	0 (0)
Ebsteins anomaly	0 (0)	1 (0)	1 (0)	0 (0)	0 (0)	1 (0.2)	0 (0)	0 (0)
None of the above	464 (13.8)	1164 (34.6)	387 (11.5)	69 (2.1)	85 (15.6)	254 (46.5)	80 (14.7)	14 (2.6)
